# Palladium(II)/Brønsted Acid-Catalyzed Enantioselective Oxidative Carbocyclization–Borylation of Enallenes**

**DOI:** 10.1002/anie.201501048

**Published:** 2015-03-24

**Authors:** Tuo Jiang, Teresa Bartholomeyzik, Javier Mazuela, Jochen Willersinn, Jan-E Bäckvall

**Affiliations:** Department of Organic Chemistry, Arrhenius Laboratory, Stockholm UniversityStockholm SE-10691 (Sweden)

**Keywords:** carbocyclization, chiral anion, chiral phosphoric acid, oxidation, palladium

## Abstract

An enantioselective oxidative carbocyclization–borylation of enallenes that is catalyzed by palladium(II) and a Brønsted acid was developed. Biphenol-type chiral phosphoric acids were superior co-catalysts for inducing the enantioselective cyclization. A number of chiral borylated carbocycles were synthesized in high enantiomeric excess.

Chiral anion-induced enantioselective transformations have emerged as challenging reactions in asymmetric catalysis.[1] In particular, the combination of chiral anions with transition metal catalysis has introduced new perspectives to the field and offers opportunities for the discovery of new enantioselective reactions.[2–7] Ever since the development of the first enantioselective hydrocarboxylation of styrenes catalyzed by Pd^II^ and a chiral phosphoric acid,[8] a number of palladium-catalyzed asymmetric protocols employing such a chiral anion strategy have been developed.[9–12] These advances have mainly focused on the α-allylation of aldehydes and ketones,[9,10] the Overman rearrangement,[11] and the Diels–Alder reactions[12] under non-oxidative conditions. On the other hand, for Pd-catalyzed oxidative transformations, especially those involving the stereoselective formation of carbon–carbon bonds, the catalytic system based on Pd^II^ and a chiral Brønsted acid has been less successful.[13–15]

Pd^II^-catalyzed oxidative carbocyclization reactions have recently emerged as an important class of chemical transformations, as they allow the rapid assembly of a variety of complex cyclic structures from substrates incorporating fewer prefunctionalizations.[16,17] In the past decade, our research group has been involved in the development of novel oxidative carbocyclization reactions using allenes as the carbon nucleophiles. Several unsaturated hydrocarbon systems have been studied extensively, such as dienallenes,[18] enallenes,[19] and allenynes.[20] Among those, the carbocyclization of enallenes, proposed to involve a vinylpalladium(II) intermediate **A**, occurred through a *syn*-carbopalladation leading to the formation of cyclic structures, which contain a new stereogenic center (Scheme 1). In order to develop an asymmetric protocol, we envisioned that by exchanging the acetate with a chiral anion, migratory insertion of the alkene into the Pd–C bond may occur in an enantioselective manner. By this means, an enantioselective carbocyclization could be accomplished under oxidative conditions. Herein, we report a Pd^II^/Brønsted acid-catalyzed enantioselective oxidative carbocyclization–borylation of enallenes.

**Scheme 1:**
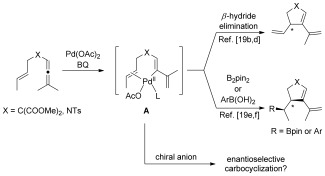
Palladium(II)-catalyzed oxidative carbocyclization of enallenes.

We chose chiral phosphates as anions of interest, owing to their stability under oxidative conditions.[21] In the initial experiment, enallene **1 a** was treated with 2.5 mol % of Pd(OAc)_2_, 5 mol % of (*R*)-BINOL phosphoric acid **3 a**, 1.0 equiv of bis(pinacolato)diboron (B_2_pin_2_) and 1.2 equiv of benzoquinone (BQ) in toluene at room temperature for 24 h. Gratifyingly, the desired borylated carbocycle **2 a** was obtained in nearly quantitative yield and the enantiomeric excess of the borylated carbocycle was determined as 30 % (Table 1, entry 1).[22] Although the enantioselectivity was low, this result clearly indicates that the anticipated chiral anion strategy was feasible for this oxidative carbocyclization reaction. We further screened a series of 3,3′-disubstituted (*R*)-BINOL phosphoric acids.[23] However, only minor improvement of the enantioselectivity was observed. 2-Naphthyl-substituted phosphoric acid **3 c** was the most effective among its analogues, but the reaction delivered the desired product in only 45 % *ee* (entry 3). Phosphoric acid **3 i** (TRIP), which was the optimal acid in previous studies,[9, 10b, 11–14] showed poor enantioselectivity in our carbocyclization reaction (entry 9).

**Table 1:** Screening of reaction conditions.[a] 
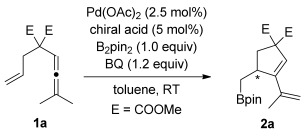

Entry	Chiral acid	Time [h]	Yield [%][b]	*ee* [%][d]
1	**3 a**	24	>95	30
2	**3 b**	24	>95	8
3	**3 c**	24	>95	45
4	**3 d**	24	67	6
5	**3 e**	24	>95	42
6	**3 f**	24	>95	10
7	**3 h**	24	>95	26
8	**3 h**	24	>95	20
9	**3 i**	16	67	24
10	**4 a**	24	>95	77
11	**4 b**	16	67	76
12	**4 c**	16	>95	40
13	**4 d**	16	>95	48
14	**4 e**	48	<5	–[e]
15[f]	**4 a**	84	>95	80
16[f,g]	**4 a**	84	>95	83
17[f,h]	**4 a**	36	>95	83
18[f,h]	**4 b**	36	>95	80
19[f,^h^i]	**4 a**	42	97 (93[c])	84

[a] Reactions were run on a 0.1 mmol scale in 1.0 mL of toluene.

[b] Yields were determined by ^1^H NMR analysis of crude reaction mixtures using anisole as the internal standard.

[c] Yield of isolated product.

[d] Determined by HPLC on a chiral stationary phase.

[e] Not determined.

[f] Using 5 mol % of Pd(OAc)_2_, 10 mol % of chiral acid in anhydrous solvent under argon at 13 °C.

[g] In 1.0 mL of *m*-xylene.

[h] In 0.5 mL of *m*-xylene.

[i] On 0.2 mmol scale with 1.5 equiv of BQ in 1.0 mL of *m*-xylene. 
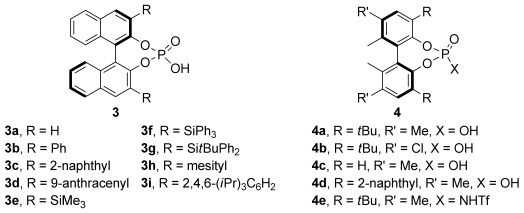

We further examined phosphoric acids with different chiral backbones. To our delight, biphenol-type phosphoric acid **4 a** with 3,3′-di-*tert*-butyl substitution was superior for rendering a highly enantioselective carbocyclization and the borylated carbocycle **2 a** was obtained in 77 % *ee* (Table 1, entry 10).[24] An analogous phosphoric acid **4 b** with electron-withdrawing chlorides attached to the backbone gave a similar outcome with regard to the enantioselectivity of the reaction (entry 11). Modification of the substituents at the 3,3′ positions resulted in a diminished enantioselectivity (entries 12 and 13). With phosphoramide **4 e**, however, the reaction hardly proceeded, probably because of the steric hindrance of the anion (entry 14). A careful screening of the solvent showed that noncoordinating arene-type solvents were effective, and *m*-xylene gave the best results. Furthermore, higher catalyst loading (5 mol %), lower temperature (13 °C), and an excess of BQ (1.5 equiv) improved both the yield (93 %) and enantioselectivity (84 % *ee*) of the reaction (entry 19).[23]

With the optimized conditions in hand, we investigated the substrate scope of the reaction. The diethyl malonate enallene **1 b** gave the cyclization product in similar yield and enantioselectivity as **1 a** (Table 2, entry 2). A significant increase in enantioselectivity (93 % *ee*) was observed with enallene **1 c**, which incorporated a cyclohexylidene allene moiety (entry 3). The ring size of the cycloalkylideneallenes had a minor influence on the enantioselectivity, as the cyclopentylidene and cycloheptylidene enallenes **1 d** and **1 e** both gave the corresponding borylated products in 92 % *ee* (entries 4 and 5). However, the borylated prodcut from cycloheptylidene substrate **1 e** was obtained in a lower yield. The reaction of the cyclooctylidene substrate **1 f** led to **2 f** in a higher yield, but the enantioselectivity was diminished slightly (88 % *ee*), probably because of a less favored coordination of the chiral catalyst to the substrate (entry 6). The borylation of unsymmetrical monomethyl allene **1 g** led to the product in low yield (34 %) and moderate enantiomeric excess (51 % *ee*) after 96 h (entry 7).[25] Evidently, this outcome indicates that the terminal disubstitution of the allene is crucial for both its reactivity and the enantioselectivity of the reaction. Better results with regard to the yield (75 %) and enantioselectivity (71 % *ee*) were obtained with methyl- and phenyl-substituted **1 h** compared to **1 g** (entry 8 versus entry 7). Modifications on the alkene moiety were unfavorable for the enantioselective carbocyclization. An internal methyl substituent on the alkene was tolerated, but the product was obtained in only 75 % *ee* (entry 9). Cinnamyl substrate **1 j**, which reacted well in the racemic reaction,[19e] completely failed under the enantioselective reaction conditions and no cyclized product was formed (entry 10).[26] The *cis*-phenyl isomer **1 k** could only be converted to the desired product to a small extent after 96 h with moderate enantioselectivity (68 % *ee*, entry 11).[27] Aza-enallene **1 l** was incompatible with the reaction conditions and decomposition of the starting material was observed, probably because of its sensitivity to the acidic environment (entry 12).[28]

**Table 2:** Scope of the enantioselective carbocyclization–borylation reaction.[a]

Entry	Enallene	Carbocycle	Time [h]	Yield [%][b]	*ee* [%][c]
1	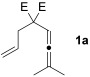	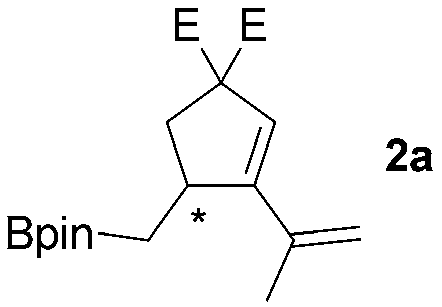	42	93	84
2	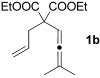	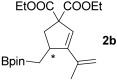	42	88	84
3	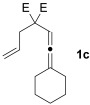	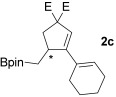	84	79	93
4	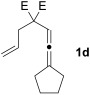	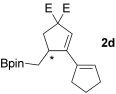	72	83	92
5	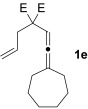	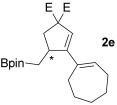	72	68	92
6	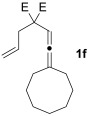	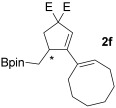	96	86	88
7	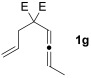	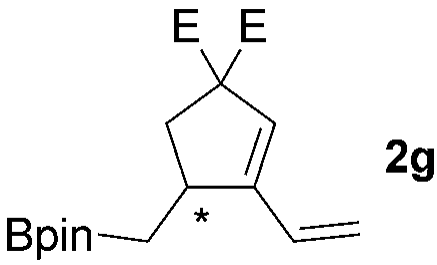	96	34	51
8	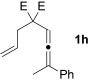	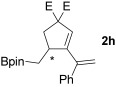	48	75	71
9	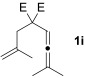	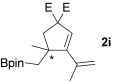	96	79	75
10	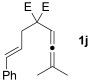	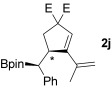	48	0	–[d]
11	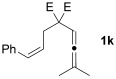	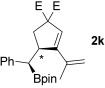	86	10	68
12	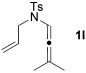	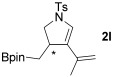	48	0	–[d]
13	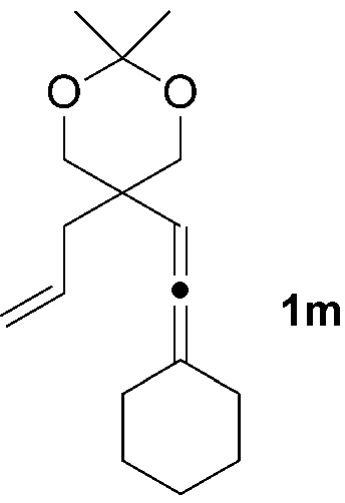	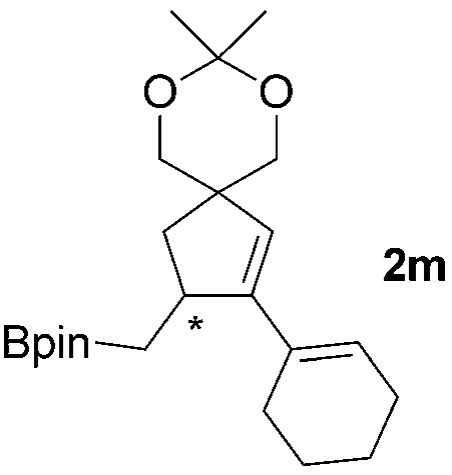	108	76	93

[a] Reaction conditions: Enallene (0.2 mmol), Pd(OAc)_2_ (5 mol %), **4 a** (10 mol %), B_2_pin_2_ (1.0 equiv), and BQ (1.5 equiv) in anhydrous *m*-xylene (1.0 mL) under argon at 13 °C.

[b] Yields of isolated products after column chromatography.

[c] Determined by HPLC on a chiral stationary phase.

[d] Not determined. E=CO_2_Me, Ts=*para*-toluenesulfonyl.

Previous studies suggest a possible coordination of the tethered group (e.g. carbonyl moiety of the malonate) to the Pd^II^ center, which may influence the outcome of the reaction.[29] A control experiment employing enallene **1 m** with a ketal tether, which cannot coordinate to Pd in the C–C bond-forming step, showed that the enantioselectivity was unaffected compared to the reaction of **1 c**, where the tether has two carbomethoxy groups. Borylated carbocycles **2 m** and **2 c** were obtained with the same enantioselectivity (93 % *ee*) and in a similar yield, though a slightly longer reaction time was required for the former (entry 13 versus entry 3).

In conclusion, we have developed a Pd^II^/Brønsted acid-catalyzed enantioselective oxidative carbocyclization–borylation of enallenes. Biphenol-type chiral phosphoric acids served as novel anionic co-catalysts for this enantioselective carbocyclization. With this protocol, a number of borylated carbocycles were formed with good to high enantioselectivity. More importantly, this work has successfully tackled the enantioselective oxidative carbocyclization reactions from a new angle.[17] We believe that this chiral anion strategy can be further applied for the development of different enantioselective oxidative carbon–carbon bond-forming reactions.
