# Supplementary material for: Palladium(II)/Brønsted Acid-Catalyzed Enantioselective Oxidative Carbocyclization–Borylation of Enallenes
Source: Angew Chem Int Ed Engl. 2015 Mar 24;54(20):6024–7. doi: 10.1002/anie.201501048 (PMC4471548; doi:10.1002/anie.201501048)
Supplement: Supplementary file 1 [file anie0054-6024-sd1.pdf]

Angewandte Chemie
Eine Zeitschrift der Gesellschaft Deutscher Chemiker

Supporting Information

German Edition: DOI:

Palladium(II)/Brønsted Acid-Catalyzed Enantioselective Oxidative Carbocyclization–Borylation of Enallenes**

*Tuo Jiang, Teresa Bartholomeyzik, Javier Mazuela, Jochen Willersinn, and Jan-E. Bäckvall**

anie_201501048_sm_miscellaneous_information.pdf

General remarks

Unless otherwise noted, all the reactions were run under argon with anhydrous conditions. All reagents were used as received from commercial suppliers. 1,4-Benzoquinone (BQ) was recrystallized from *n*-hexane before use. Phosphorus(V) oxychloride ($POCl_3$) was distilled over Na under argon and stored in a desiccator in the fridge. Arene-type solvents were dried over activated 4Å molecular sieves under argon. Room temperature is 23 ± 1 ^{o}C. Reactions were monitored using E. Merck silica gel 60 F254 plates (TLC analysis). TLC plates were visualized with UV light (254 nm), iodine, $KMnO_4$ stain or CAM stain (Cerium Ammonium Molybdate). Flash chromatography was carried out with 60Å (particle size 35-70 μm) normal flash silica gel. NMR spectra were recorded at 400 MHz (H) and at 100 MHz (C), respectively. Chemical shifts (δ) are reported in ppm, using the residual solvent peak in $CDCl_3$ (H: δ = 7.26 ppm and C: δ = 77.16 ppm) as internal standard, and coupling constants (J) are given in Hz. HRMS were recorded using ESI-TOF techniques. The enantiomeric excess of compounds was determined by chiral HPLC using the corresponding racemic compounds as the reference.

Experimental section

Starting materials and chiral catalysts

Allenyl bromides were prepared based on the reported protocols.[1] Due to the instability, all allenyl bromides were used immediately after the preparation.

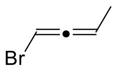

^{1}H NMR ($CDCl_3$, 400 MHz): δ 5.91 (dq, J = 5.7, 2.4 Hz, 1H), 5.34 (dq, J = 7.3, 5.7 Hz, 1H), 1.82 (dd, J = 7.3, 2.4 Hz, 3H).

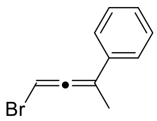

^{1}H NMR ($CDCl_3$, 400 MHz): δ 7.46 – 7.44 (m, 2H), 7.39 – 7.35 (m, 2H), 7.31 – 7.27 (m, 1H), 6.25 (q, J = 2.1 Hz, 1H), 2.25 (d, J = 2.1 Hz, 3H).

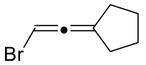

^{1}H NMR ($CDCl_3$, 400 MHz): δ 5.93 (quintet, J = 3.0 Hz, 1H), 2.58 – 2.45 (m, 4H), 1.75 – 1.69 (m, 4H).

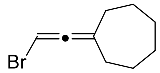

^{1}**H NMR** ($CDCl_3$, 400 MHz): δ 5.81 (quintet, J = 1.8 Hz, 1H), 2.41 – 2.37 (m, 4H), 1.68 – 1.64 (m, 4H), 1.59 – 1.53 (m, 4H).

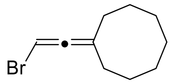

^{1}**H NMR** ($CDCl_3$, 400 MHz): δ 5.85 (quintet, J = 1.6 Hz, 1H), 2.31 – 2.26 (m, 4H), 1.71 – 1.66 (m, 4H), 1.56 – 1.50 (m, 6H).

Enallenes were prepared according to our previous reports.[2,3] A general procedure is shown below.

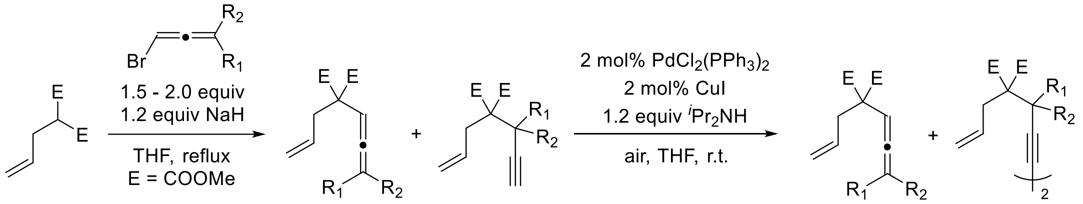

New enallene substrates are listed below.

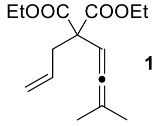

1b

From diethyl allylmalonate (15 mmol), enallene **1b** was obtained as a light yellow oil (1.40 g, 35% for two steps).

^{1}**H NMR** ($CDCl_3$, 400 MHz): δ 5.71 (ddt, J = 17.1, 10.0, 7.1 Hz, 1H), 5.51 (quintet, J = 2.9 Hz, 1H), 5.10 – 5.03 (m, 2H), 4.22 – 4.14 (m, 4H), 2.73 (dt, J = 7.1, 1.1 Hz, 2H), 1.69 (d, J = 2.9 Hz, 6H), 1.25 (t, J = 7.1 Hz, 6H).

^{13}**C NMR** ($CDCl_3$, 100 MHz): δ 201.9, 170.4 (2C), 133.1, 118.6, 99.9, 88.5, 61.6 (2C), 58.1, 38.9, 20.2 (2C), 14.2 (2C).

HRMS (ESI) m/z for $C_{15}H_{22}O_4Na$ $[M+Na]^+$ calcd 289.1410, found 289.1399.

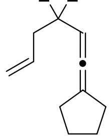

1d

From dimethyl allylmalonate (10 mmol), enallene **1d** was obtained as a light yellow oil (1.27 g, 48% for two steps).

^{1}**H NMR** ($CDCl_3$, 400 MHz): δ 5.71 (ddt, J = 17.1, 10.1, 7.2 Hz, 1H), 5.57 (quintet, J = 4.0 Hz, 1H), 5.10 – 5.04 (m, 2H), 3.72 (s, 6H), 2.74 (dt, J = 7.2, 1.2 Hz, 2H), 2.39 – 2.34 (m, 4H), 1.69 – 1.65 (m, 4H).

^{13}**C NMR** ($CDCl_3$, 100 MHz): δ 197.5, 170.8 (2C), 133.2, 118.6, 108.7, 90.7, 58.7, 52.7 (2C), 38.9, 31.1 (2C), 27.1 (2C).

HRMS (ESI) m/z for $C_{15}H_{20}O_4Na$ $[M+Na]^+$ calcd 287.1254, found 287.1256.

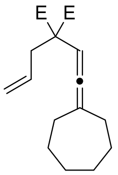

1e

From dimethyl allylmalonate (8.8 mmol), enallene **1e** was obtained as a light yellow oil (1.35 g, 53% for two steps).

^{1}H NMR ($CDCl_3$, 400 MHz): δ 5.73 (ddt, J = 17.1, 10.1, 7.2 Hz, 1H), 5.50 (quintet, J = 5.50 Hz, 1H), 5.11 – 5.04 (m, 2H), 3.72 (s, 6H), 2.75 (dt, J = 7.2, 1.2 Hz, 2H), 2.27 – 2.23 (m, 4H), 1.64 – 1.60 (m, 4H), 1.55 – 1.52 (m, 4H).

^{13}C NMR ($CDCl_3$, 100 MHz): δ 201.8, 170.9 (2C), 133.2, 118.6, 109.4, 88.1, 58.5, 52.7 (2C), 39.2, 32.2 (2C), 29.5 (2C), 28.5 (2C).

HRMS (ESI) m/z for $C_{17}H_{24}O_4Na$ $[M+Na]^+$ calcd 315.1567, found 315.1571.

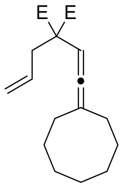

1f

From dimethyl allylmalonate (2.6 mmol), enallene **1f** was obtained as a light yellow oil (455 mg, 57% after two steps).

^{1}H NMR ($CDCl_3$, 400 MHz): δ 5.74 (ddt, J = 17.1, 10.1, 7.2 Hz, 1H), 5.55 (quintet, J = 2.2 Hz, 1H), 5.11 – 5.04 (m, 2H), 3.72 (s, 6H), 2.75 (dt, J = 7.2, 1.1 Hz, 2H), 2.20 – 2.15 (m, 4H), 1.65 – 1.51 (m, 10 H).

^{13}C NMR ($CDCl_3$, 100 MHz): δ 202.0, 170.9 (2C), 133.2, 118.6, 109.6, 88.6, 58.4, 52.7 (2C), 39.4, 31.3 (2C), 27.0 (2C), 26.8 (2C), 26.0.

HRMS (ESI) m/z for $C_{18}H_{26}O_4Na$ $[M+Na]^+$ calcd 329.1723, found 329.1740.

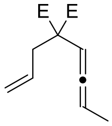

1g

From dimethyl allylmalonate (8 mmol), enallene **1g** was obtained as a light yellow oil (415 mg, 23% for two steps). **Note:** After the homo-coupling reaction, enallene **1g** was further purified by Kugelrohr distillation.

^{1}H NMR ($CDCl_3$, 400 MHz): δ 5.69 (ddt, J = 17.1, 10.0, 7.2 Hz, 1H), 5.59 (dq, J = 6.4, 3.2 Hz, 1H), 5.31 (dq, J = 7.1, 6.4 Hz, 1H), 5.10 – 5.05 (m, 2H), 3.72 (s, 3H), 3.71 (s, 3H), 2.74 (dt, J = 7.2, 1.1 Hz, 2H), 1.66 (dd, J = 7.2, 3.2 Hz, 3H).

^{13}C NMR ($CDCl_3$, 100 MHz): δ 204.5, 170.6, 170.5, 132.9, 118.9, 90.3, 89.8, 58.0, 52.8, 52.8, 38.7, 13.9.

HRMS (ESI) m/z for $C_{12}H_{16}O_4Na$ $[M+Na]^+$ calcd 247.0941, found 247.0953.

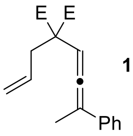

1h

From dimethyl allylmalonate (10 mmol), enallene **1h** was obtained as a light orange oil (1.49 g, 50% for two steps).

^{1}H NMR ($CDCl_3$, 400 MHz): δ 7.39 – 7.37 (m, 2H), 7.33 – 7.30 (m, 2H), 7.22 (m, 1H), 5.97 (q, J = 2.9 Hz, 1H), 5.74 (ddt, J = 17.0, 10.1, 7.2 Hz, 1H), 5.11 – 5.05 (m, 2H), 3.75 (s, 3H), 3.70 (s, 3H), 2.80 (dt, J = 7.2, 1.1 Hz, 1H), 2.11 (d, J = 2.9 Hz, 3H).

^{13}C NMR ($CDCl_3$, 100 MHz): δ 204.4, 170.5, 170.5, 136.1, 132.8, 128.5 (2C), 127.3, 126.0 (2C), 119.1, 105.2, 92.6, 58.8, 52.9, 52.8, 39.3, 16.8.

HRMS (ESI) m/z for $C_{18}H_{20}O_4Na$ $[M+Na]^+$ calcd 323.1254, found 323.1237.

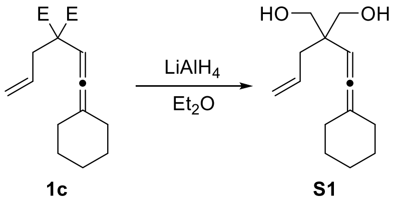

To a suspension of $LiAlH_4$ (314 mg, 8.4 mmol) in Et_2O (10 mL) was added a solution of enallene **1a** (769 mg, 2.8 mmol) in Et_2O (10 mL) dropwise followed by vigorous stirring for 2 h at room temperature. The reaction mixture was diluted with Et_2O and quenched carefully with $Na_2SO_4{\cdot}10H_2O$/celite (1:1 mixture). Filtration and concentration gave a residue, which was purified by flash column chromatography (petroleum ether/EtOAc 4/1) to give **S1** as a colorless oil (454 mg, 73%).

^{1}H NMR ($CDCl_3$, 400 MHz): δ 5.85 (ddt, J = 17.1, 10.0, 7.4 Hz, 1H), 5.14 – 5.07 (m, 2H), 4.85 (quintet, J = 2.1 Hz, 1H), 3.64 (dd, J = 11.1, 5.7 Hz, 2H), 3.59 (dd, J = 11.1, 6.8 Hz, 2H), 2.20 (dt, J = 7.4, 1.1 Hz, 2H), 2.14 – 2.10 (m, 4H), 2.02 (d, J = 5.7 Hz, 1H), 2.00 (d, J = 6.8 Hz, 1H), 1.68 – 1.60 (m, 2H), 1.56 – 1.47 (m, 4H).

^{13}C NMR ($CDCl_3$, 100 MHz): δ 198.3, 134.4, 117.9, 105.2, 90.7, 68.1 (2C), 44.9, 38.0, 31.9 (2C), 27.5 (2C), 26.1.

HRMS (ESI) m/z for $C_{14}H_{22}O_2Na$ $[M+Na]^+$ calcd 245.1512, found 245.1508.

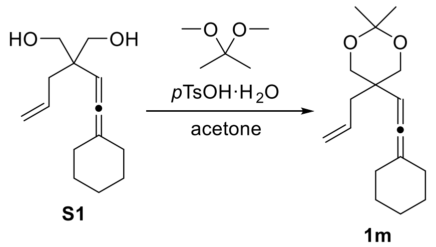

To a solution of **S1** (505 mg, 2.27 mmol) in acetone (11 mL) was added 2,2-dimethoxypropane (0.7 mL, 5.7 mmol) and pTsOH·H_2O (9 mg, 0.05 mmol). The reaction mixture was stirred at room temperature for 3 h and

quenched with sat. $NaHCO_3$ (aq.). Acetone was evaporated and the residue was extracted with Et_2O, washed with brine, dried over $MgSO_4$. Concentration gave pure **1m** as a colorless oil (588 mg, 99%).

^{1}H NMR ($CDCl_3$, 400 MHz): δ 5.81 (ddt, J = 17.0, 10.0, 7.4 Hz, 1H), 5.14 – 5.08 (m, 2H), 4.83 (quintet, J = 2.0 Hz, 1H), 3.69 (d, J = 11.8 Hz, 2H), 3.57 (d, J = 11.8 Hz, 2H), 2.36 (dt, J = 7.4, 1.1 Hz, 2H), 2.18 – 2.05 (m, 4H), 1.68 – 1.61 (m, 2H), 1.59 – 1.46 (m, 4H), 1.42 (s, 3H), 1.42 (s, 3H).

^{13}C NMR ($CDCl_3$, 100 MHz): δ 198.3, 134.3, 118.1, 105.2, 97.9, 90.8, 67.8 (2C), 38.3, 37.1, 31.8 (2C), 27.6 (2C), 26.8, 26.2, 21.1.

HRMS (ESI) m/z for $C_{17}H_{26}O_2Na$ $[M+Na]^+$ calcd 285.1825, found 285.1814.

C_2-symmetric chiral phosphoric acids were prepared according to the literature.[4] Biphenol-type chiral phosphoric acids were prepared based on the reported protocol, with further modification.[5,6]

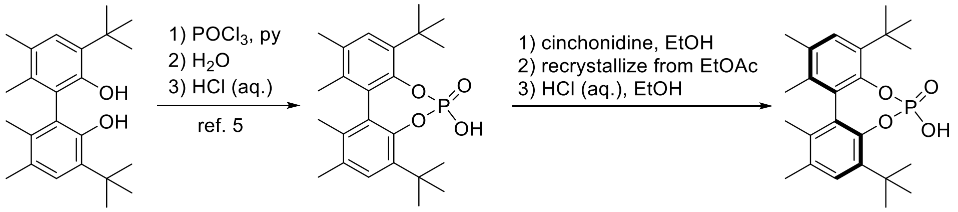

At 80 °C, racemic biphenol phosphoric acid (6.08 g, 14.6 mmol) was dissolved in absolute EtOH (150 mL). (–)-cinchonidine (4.48 g, 96%, 14.6 mmol) was added in one-portion and the mixture was stirred at room temperature for 2 h. Concentration of the reaction mixture gave the crude diastereomers (1:1 ratio), which was used directly for the next step.

The crude diastereomers was dissolved in minimum amount of boiling EtOAc and heated at 80 °C until white solid precipitated. The precipitate was filtered off when hot to give the pure complex of (*S*)-phosphoric acid **4a** & (–)-cinchonidine. The complex was dissolved in absolute EtOH followed by the addition of 50 mL 6N HCl (aq.). The biphasic mixture was stirred vigorously at room temperature for 1 h and white precipitation was filtered off and washed with deionized water. The solid was collected and dried under vacuum to give enantiopure (*S*)-phosphoric acid **4a** as a white powder (2.05 g, 34% based on the racemic biphenol phosphoric acid, theoretical yield = 50%).

^{1}H NMR ($CDCl_3$, 400 MHz): δ 7.20 (s, 2H), 2.25 (s, 6H), 1.83 (s, 6H), 1.46 (s, 18H).

^{13}C NMR ($CDCl_3$, 100 MHz): δ 144.7 (d, J = 7.0 Hz, 2C), 137.9 (d, J = 4.2 Hz, 2C), 135.2 (d, J = 1.4 Hz, 2C), 133.7 (d, J = 1.8 Hz, 2C), 129.2 (2C), 128.7 (d, J = 1.8 Hz, 2C), 35.0 (2C), 31.6 (6C), 20.5 (2C), 16.8 (2C).

^{31}P NMR ($CDCl_3$, 162 MHz): δ -0.56.

$[\alpha]_D^{23}$ = + 122.9 (c = 1.0, $CHCl_3$).

HRMS (ESI) m/z for $C_{24}H_{32}O_4P$ $[M-H]^-$ calcd 415.2044, found 415.2059.

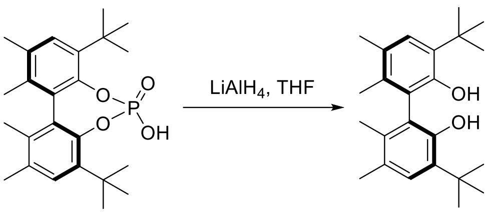

4a **S2**

At 0 °C, to a solution of (*S*)-phosphoric acid **4a** (2.05 g, 4.9 mmol) in THF (49 mL) was added $LiAlH_4$ (651 mg, 17.2 mmol) portionwise followed by reflux for 18 h. The reaction mixture was diluted with THF and quenched carefully with $Na_2SO_4 \cdot 10H_2O$/celite (1:1 mixture). Filtration and concentration gave pure (*S*)-biphenol **S2** as a white solid (1.26 g, 73%). Analytical data was in accordance with the literature.[5]

The debutylated biphenol **S3** was prepared from **S2** according to the literature.[7]

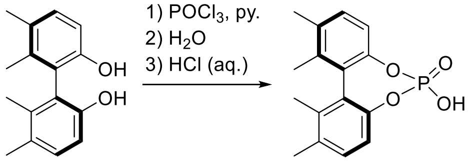

S3 **4c**

To a solution of (*S*)-biphenol **S3** (469 mg, 1.94 mmol) in pyridine (20 mL) was added $POCl_3$ (0.36 mL, 3.88 mmol) dropwise. The reaction mixture was heated at reflux for 3 h and cooled to room temperature. Deionized water (5 mL) was added dropwise to the reaction mixture followed by reflux for additional 4 h. Pyridine was removed by rotary evaporation and the residue was dissolved in DCM and acidified with 4N HCl (aq.). The aqueous phase was extracted thoroughly with DCM and the combined organic phase was washed with 4N HCl (aq.), dried over Na_2SO_4, and concentrated. The residue was purified by flash column chromatography (DCM/MeOH 5/1 to 2/1) to give a white solid. The white solid was dissolved in DCM, acidified with 4N HCl (aq.) and dried over Na_2SO_4. Removal of the solvent gave pure (*S*)-phosphoric acid **4c** as a white powder (412 mg, 70%).

^{1}H NMR ($CDCl_3$, 400 MHz): δ 7.20 (d, J = 8.1 Hz, 2H), 7.09 (d, J = 8.1 Hz, 2H), 2.31 (s, 6H), 2.04 (s, 6H).

^{13}C NMR ($CDCl_3$, 100 MHz): δ 146.7 (d, J = 8.6 Hz, 2C), 137.6 (2C), 135.1 (d, J = 2.0 Hz, 2C), 130.6 (2C), 127.1 (2C), 118.3 (d, J = 3.7 Hz, 2C), 20.4 (2C), 17.7 (2C).

^{31}P NMR ($CDCl_3$, 162 MHz): δ -3.34.

$[\alpha]_D^{23}$ = + 157.0 (c = 0.1, $CHCl_3$).

HRMS (ESI) m/z for $C_{16}H_{16}O_4P$ $[M-H]^-$ calcd 303.0792, found 303.0795.

The brominated (*S*)-biphenol **S4** was prepared according to the literature.[7]

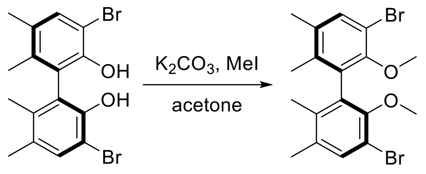

To a solution of **S4** (166 mg, 0.41 mmol) in acetone (5 mL) was added K_2CO_3 (198 mg, 1.44 mmol) and MeI (0.15 mL, 2.46 mmol) and the mixture was heated at reflux for 18 h. Acetone was removed by rotary evaporation and the residue was dissolved with DCM and washed with deionized water. The aqueous phase was extracted thoroughly with DCM and the combined organic phase was washed with brine, dried over Na_2SO_4, and concentrated. The residue was obtained as a white solid (177 mg, 99%) and used directly for the next step. Analytical data of **S5** was in accordance with the literature.

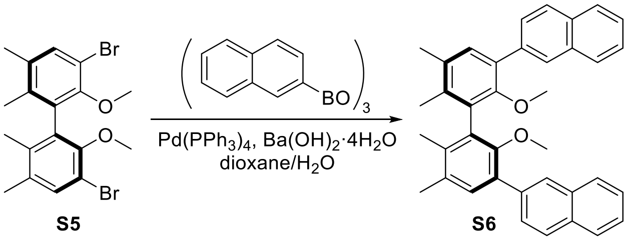

To a mixture of **S5** (177 mg, 0.41 mmol), 2-naphthylboroxine (663 mg, 1.44 mmol), $Pd(PPh_3)_4$ (47 mg, 0.04 mmol) and $Ba(OH)_2{\cdot}4H_2O$ (453 mg, 1.44 mmol) was added freshly degassed dioxane/H_2O (4 mL, 3/1). The reaction mixture was heated at 90 °C for 18 h. Dioxane was removed by rotary evaporation and the residue was dissolved in DCM and washed with 2 N HCl (aq.). The aqueous phase was extracted thoroughly with DCM and the combined organic phase was washed brine, dried over Na_2SO_4, and concentrated. The residue was purified by flash column chromatography (pentane/DCM 5/1) to give **S6** as a white solid (209 mg, 98 %).

^{1}H NMR ($CDCl_3$, 400 MHz): δ 8.18 (br. s, 2H), 7.97 – 7.89 (m, 8H), 7.56 – 7.53 (m, 4H), 7.41 (s, 2H), 3.32 (s, 6H), 2.46 (s, 6H), 2.13 (s, 6H).

^{13}C NMR ($CDCl_3$, 100 MHz): δ 153.7, 137.1, 135.9, 133.7, 132.6, 132.5, 132.3, 131.7, 131.5, 128.2, 127.9, 127.7, 127.7, 127.6, 126.0, 125.8, 60.4, 20.3, 17.0.

$[\alpha]_D^{23}$ = + 220.0 (c = 2.0, $CHCl_3$).

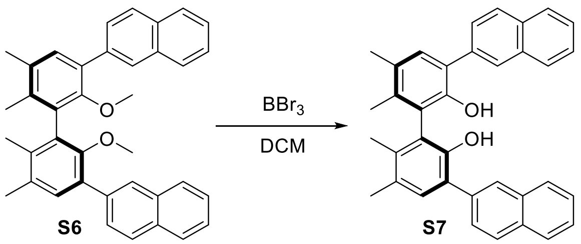

At 0 °C, to a solution of **S6** (209 mg, 0.40 mmol) in DCM (4 mL) was added BBr_3 (0.84 mL, 1.0 M in hexane, 0.84 mmol) dropwise. The reaction was maintained at 0 °C for 3 h and quenched by slow addition of deionized water. The aqueous phase was extracted thoroughly with DCM and the combined organic phase was washed brine, dried over Na_2SO_4 and passed through a short silica column. Removal of the solvent gave pure **S7** as a white solid (193 mg, 98%).

^{1}H NMR ($CDCl_3$, 400 MHz): δ 8.07 (br. s, 2H), 7.91 (d, J = 8.6 Hz, 2H), 7.89 – 7.85 (m, 4H), 7.76 (dd, J = 8.6, 1.8 Hz, 2H), 7.50 – 7.48 (m, 4H), 7.36 (s, 2H), 5.00 (s, 2H), 2.37 (s, 6H), 2.05 (s, 6H).

^{13}C NMR ($CDCl_3$, 100 MHz): δ 148.8, 136.7, 135.6, 133.7, 132.6, 132.5, 129.6, 128.2, 128.0, 128.0, 127.8 (2C), 126.2, 126.0, 125.7, 121.8, 20.0, 16.7.

$[\alpha]_D^{23}$ = + 252.6 (c = 0.8, $CHCl_3$).

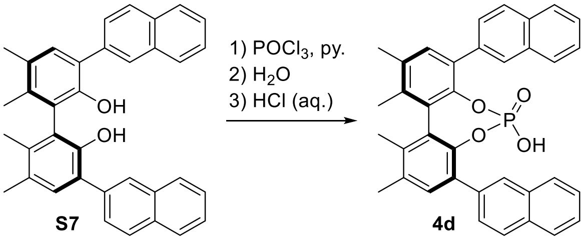

To a solution of **S7** (347 mg, 0.7 mmol) in pyridine (7 mL) was added $POCl_3$ (0.13 mL, 0.14 mmol) dropwise. The reaction mixture was heated at 90 °C for 18 h and cooled to room temperature. Deionized water (5 mL) was added dropwise to the reaction mixture followed by heating at 90 °C for additional 8 h. Pyridine was removed by rotary evaporation and the residue was dissolved in DCM and acidified with 4N HCl (aq.). The aqueous phase was extracted thoroughly with DCM and the combined organic phase was washed with deionized water, dried over Na_2SO_4, and concentrated. The residue was purified by flash column chromatography (DCM/MeOH 20/1) to give a white solid. The white solid was dissolved in DCM, acidified with 4N HCl (aq.) and dried over Na_2SO_4. Removal of the solvent gave pure (*S*)-phosphoric acid **4d** as a white powder (367 mg, 94%).

^{1}H NMR ($CDCl_3$, 400 MHz): δ 7.86 (br. s, 2H), 7.70 – 7.67 (m, 2H), 7.57 – 7.54 (m, 2H), 7.53 – 7.51 (m, 2H), 7.50 – 7.46 (m, 2H), 7.31 (br. s, 2H), 7.26 – 7.24 (m, 4H), 2.40 (s, 6H), 2.14 (s, 6H).

^{13}C NMR ($CDCl_3$, 100 MHz): δ 143.5 (d, J = 9.2 Hz, 2C), 136.9 (2C), 134.7 (2C), 134.5 (2C), 133.3 (2C), 132.6 (2C), 132.2 (2C), 131.6 (d, J = 9.2 Hz, 2C), 128.4 (2C), 128.4 (2C), 128.1 (2C), 127.6 (4C), 127.5 (2C), 125.7 (2C), 125.7 (2C), 20.5 (2C), 17.7 (2C).

^{31}P NMR ($CDCl_3$, 162 MHz): δ 1.17.

$[\alpha]_D^{23}$ = + 319.7 (c = 1.77, $CHCl_3$).

HRMS (ESI) m/z for $C_{36}H_{28}O_4P$ $[M-H]^-$ calcd 555.1731, found 555.1705.

(*S*)-Phosphoramide **4e** was prepared based on the literature protocol.[8]

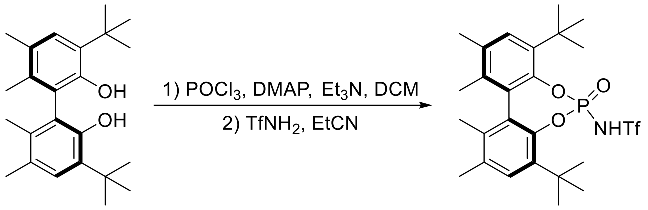

S2 **4e**

At 0 °C, to a solution of **S2** (213 mg, 0.6 mmol) in DCM (3 mL) were added Et_3N (0.59 mL, 4.2 mmol), $POCl_3$ (67 µL, 0.72 mmol) and DMAP (147 mg, 1.2 mmol) at 0 °C. After stirring at room temperature for 1 h, EtCN (3 mL) was added followed by the addition of $TfNH_2$ (179 mg, 1.2 mmol). Then the reaction was heated 100 °C for 16 h. The reaction was quenched with deionized water, and extracted with Et_2O. The combined organic phase was washed with sat. $NaHCO_3$ (aq.), 4N HCl (aq.), dried over Na_2SO_4 and concentrated. The residue was purified by flash column chromatography (100% Et_2O) to give a pale white solid. The solid was dissolved in DCM, acidified with 4N HCl (aq.) and dried over Na_2SO_4. Removal of the solvent gave (*S*)-phosphoramide **4e** as a white powder (276 mg, 84%).

^{1}H NMR ($CDCl_3$, 400 MHz): *δ* 7.23 (s, 1H), 7.22 (s, 1H), 2.26 (s, 6H), 1.84 (s, 3H), 1.79 (s, 3H), 1.47 (s, 9H), 1.44 (s, 9H).

^{13}C NMR ($CDCl_3$, 100 MHz): *δ* 144.0 (d, *J* = 10.5 Hz, 1C), 143.6 (d, *J* = 9.1 Hz, 1C), 138.3 (d, *J* = 4.3 Hz, 1C), 137.3 (d, *J* = 4.9 Hz, 1C), 135.8, 135.7, 134.6 (d, *J* = 1.5 Hz, 1C), 134.4 (d, *J* = 1.5 Hz, 1C), 129.4 (2C), 128.5, 128.3, 119.3 (q, *J* = 321.2 Hz, 1C), 119.3 (q, *J* = 321.2 Hz, 1C), 35.1, 34.8, 31.8 (3C), 31.3 (3C), 20.6, 20.5, 16.8, 16.7.

^{31}P NMR ($CDCl_3$, 162 MHz): *δ* -9.05.

$[\alpha]_D^{23}$ = + 122.9 (c = 1.0, $CHCl_3$).

HRMS (ESI) m/z for $C_{25}H_{32}O_5F_3NPS$ $[M-H]^-$ calcd 546.1696, found 546.1721.

Optimization of the reaction parameters.

Initial screening of solvents.[a]

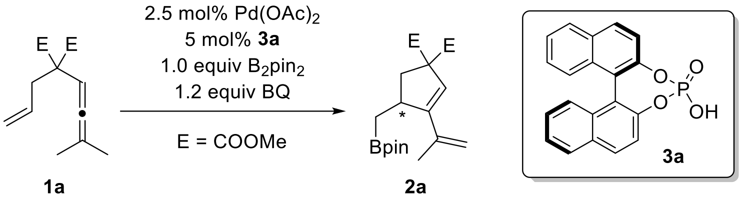

Entry	Solvent[b]	Time (h)	Yield (%)[c]	*ee* (%)[d]
1	toluene	24	> 95	30
2	acetone	70	75	8
3	DCE	70	76	8
4	DCM	70	70	8
5	EtOAc	70	85	10
6	THF	70	34	6
7	MeCN	70	0	–[e]
8	DMF	70	6	–[e]

(a) Reaction scale: Enallene **1a** (0.1 mmol) in the indicated solvent (1.0 mL) at room temperature. (b) Regular solvent. (c) Determined by ^{1}H NMR analysis of the crude mixture with anisole as the internal standard. (d) Determined by chiral HPLC. (e) Not determined.

Screening of different (*R*)-BINOL phosphoric acids.[a]

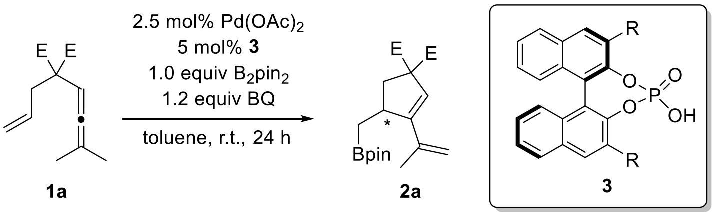

Entry	R	Yield (%)[b]	*ee* (%)[c]
1	H, **3a**	> 95	30
2	Ph, **3b**	> 95	8
3	2-naphthyl, **3c**	> 95	45
4	9-anthracenyl, **3d**	67	6
5	TMS, **3e**	> 95	42
6	$SiPh_3$, **3f**	> 95	10
7	Si^tBuPh_2, **3g**	> 95	26
8	mesityl, **3h**	> 95	20
9[d]	2,4,6-tri-^{i}Pr-C_6H_2, **3i**	67	24
10	Me, **3j**	> 95	18
11	allyl, **3k**	> 95	45
12	CO^tBu, **3l**	> 95	24
13	3,5-di-CF_3-C_6H_3, **3m**	> 95	2
14	3,5-di-^{t}Bu-C_6H_3, **3n**	> 95	8

(a) Reaction scale: Enallene **1a** (0.1 mmol) in regular toluene (1.0 mL) at room temperature. (b) Determined by ^{1}H NMR analysis of the crude mixture with anisole as the internal standard. (c) Determined by chiral HPLC. (d) Reaction time is 16 h.

Screening of phosphoric acids with different backbones.[a]

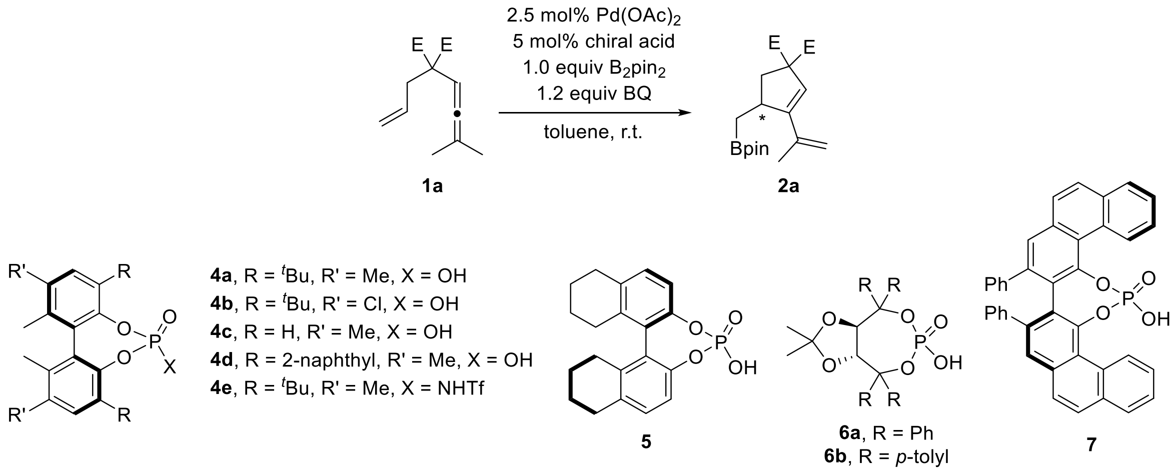

Entry	Chiral phosphoric acid	Time (h)	Yield (%)[b]	*ee* (%)[c]
1	**4a**	24	> 95	77
2	**4b**	16	67	76
3	**4c**	16	> 95	40
4	**4d**	16	> 95	48
5	**4e**	48	< 5	–[d]
6	**5**	16	> 95	30
7	**6a**	40	68	20
8	**6b**	94	22	19
9[d]	**7**	60	70	40

(a) Reaction scale: Enallene **1a** (0.1 mmol) in regular toluene (1.0 mL) at room temperature. (b) Determined by ^{1}H NMR analysis of the crude mixture with anisole as the internal standard. (c) Determined by chiral HPLC. (d) Not determined.

Screening of different palladium salts.[a]

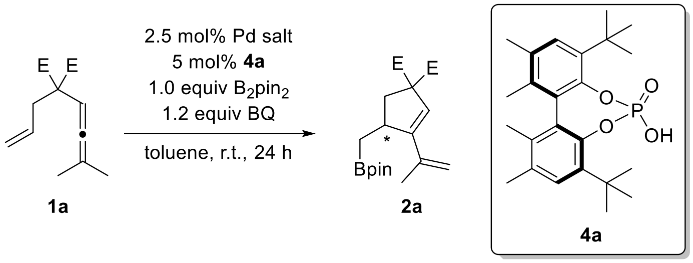

Entry	Pd salt	Yield (%)[b]	*ee* (%)[c]
1[d]	Pd_2dba_3	14	70
2[d]	$Pd_2dba_3 \cdot CHCl_3$	23	70
3	$Pd(PPh_3)_4$	48	47

4	Pd/C	0	–[e]
5	$PdCl_2$	0	–[e]
6	$PdCl_2(PPh_3)_2$	0	–[e]
7	$PdCl_2(PhCN)_2$	0	–[e]
8	$PdCl_2(DMSO)_2$	0	–[e]
9	$PdBr_2$	0	–[e]
10	$Pd(OAc)_2$	> 95	77
11[f]	$Pd(OAc)_2$	> 95	79
12[f]	$Pd(OOCCF_3)_2$	> 95	44

(a) Reaction scale: Enallene **1a** (0.1 mmol) in regular toluene (1.0 mL) at room temperature. (b) Determined by ^{1}H NMR analysis of the crude mixture with anisole as the internal standard. (c) Determined by chiral HPLC. (d) With 1.25 mol% of the Pd salt. (e) Not determined. (f) In anhydrous toluene under argon for 48 h.

Screening of arene-type solvents under inert & anhydrous conditions.[a,b]

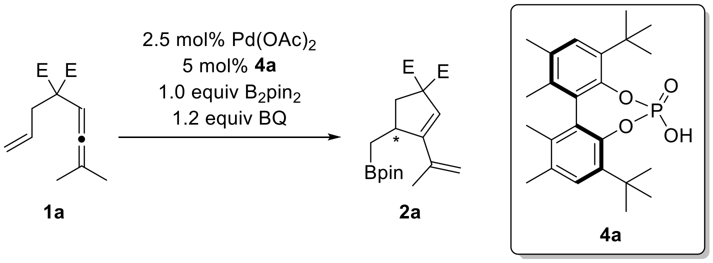

Entry	Solvent	Temp (°C)	Time (h)	Yield (%)[c,d]	*ee* (%)[e]
1	toluene	r.t.	48	> 95	79
2	toluene	13	84	71	80
3	benzene	13	84	85	73
4	$PhCF_3$	13	84	> 95	80
5	PhCl	13	60	> 95	73
6	*o*-xylene	13	84	> 95	79
7	*m*-xylene	13	84	85	80
8	*p*-xylene	13	84	85	77
9[f]	toluene	13	84	> 95	80
10[f]	$PhCF_3$	13	60	> 95	81
11[f]	*m*-xylene	13	84	> 95	83
12[g]	*m*-xylene	13	60	> 95	83
13[f,h]	*m*-xylene	13	36	> 95	83
14[f,h]	*m*-xylene	3	66	> 95	83
15[g,h]	*m*-xylene	3	48	> 95	83

(a) Reaction scale: Enallene **1a** (0.1 mmol) in the indicated solvent (1.0 mL). (b) In anhydrous solvent under argon. (c) Determined by ^{1}H NMR analysis of the crude mixture with anisole as the internal standard. (d) Isolated yield in parenthesis. (e) Determined by chiral HPLC. (f) Using $Pd(OAc)_2$ (5 mol%) and **4a** (10 mol%). (g) Using $Pd(OAc)_2$ (10 mol%) and **4a** (20 mol%). (h) In 0.5 mL of *m*-xylene.

Screening of benzoquinone-type oxidants.[a]

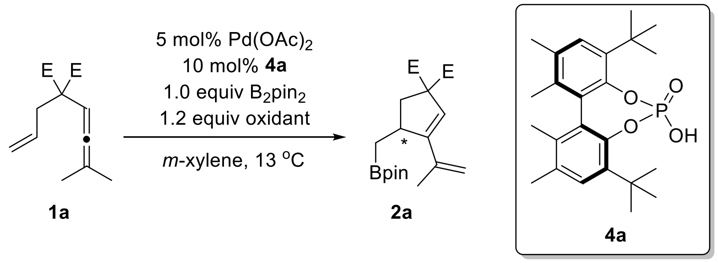

Entry	Oxidant	Time (h)	Yield (%)[b,c]	ee (%)[d]
1	BQ	36	> 95	83
2	tetra-F-BQ	60	40	58
3	2,6-di-Me-BQ	36	> 95	76
4	2,6-di-OMe-BQ	60	> 95	78
5	2-Cl-BQ	60	80	34
6	2,6-di-^{t}Bu-BQ	60	47	80
7[e]	BQ	36	> 95	81
8[f]	BQ	36	> 95 (93)	83
8[f,g]	BQ	42	97 (93)	84
9[f,h]	BQ	48	94 (88)	84

(a) Reaction scale: Enallene **1a** (0.1 mmol) in anhydrous *m*-xylene solvent (0.5 mL) under argon. (b) Determined by ^{1}H NMR analysis of the crude mixture with anisole as the internal standard. (c) Isolated yield in parenthesis. (d) Determined by chiral HPLC. (e) Using BQ (1.0 equiv.). (f) With enallene **1a** (0.2 mmol) in anhydrous *m*-xylene solvent (1.0 mL) under argon. (g) Using BQ (1.5 equiv.). (h) Using BQ (1.8 equiv.).

General procedure for the Pd(II)/Brønsted acid-catalyzed enantioselective oxidative carbocyclization-borylation of enallenes

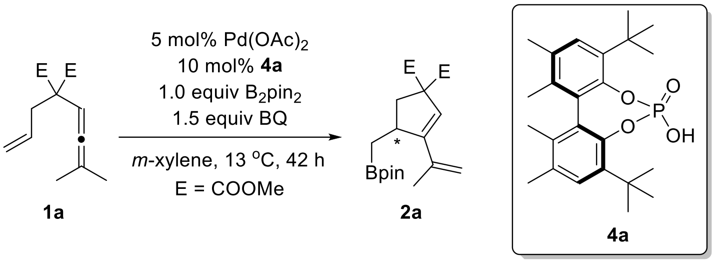

In a microwave vial was charged enallene **1a** (47.6 mg, 0.2 mmol), $Pd(OAc)_2$ (2.2 mg, 5 mol%), (*S*)-phosphoric acid **4a** (8.3 mg, 10 mol%), B_2pin_2 (51.8 mg, 98%, 0.2 mmol), BQ (32.4 mg, 0.3 mmol) and *m*-xylene (1 mL). The vial was sealed with a rubber cap and maintained at 13 °C for 42 h. The reaction was quenched with sat. $Na_2S_2O_3$ (aq.) and extracted with Et_2O. The combined organic phase was washed with brine, dried over Na_2SO_4 and concentrated. The residue was purified by flash column chromatography (pentane/Et_2O, 10/1) to afford **2a** (68 mg, 93%). *ee* = 84%.

Spectral data was in accordance with the literature.[9]

NOTE: The carbon adjacent to boron may not have a visible peak in ^{13}C spectrum. However, HSQC experiments can clearly indicate the signals of those carbons.

Retention time: 31.5 & 37.4 min, CHIRALCEL®-ID, *iso*-hexane/*iso*-propanol = 99/1, 25 °C, 0.3 mL/min.

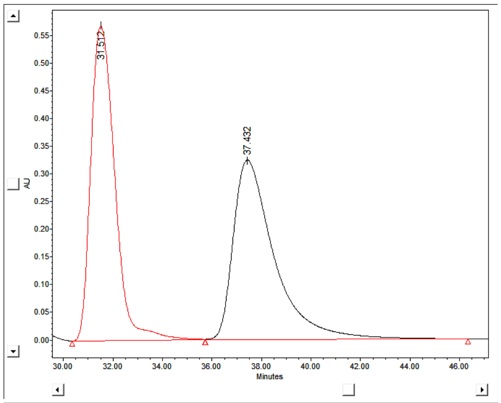

E	Name	Retention Time (min)	Area (μV*sec)	% Area	Height (μV)	Int Type	Amount	Units	Peak Type	Peak Codes
1		31.512	38024406	50.60	568109	BB			Unknown	
2		37.432	37128740	49.40	323350	Bb			Unknown	

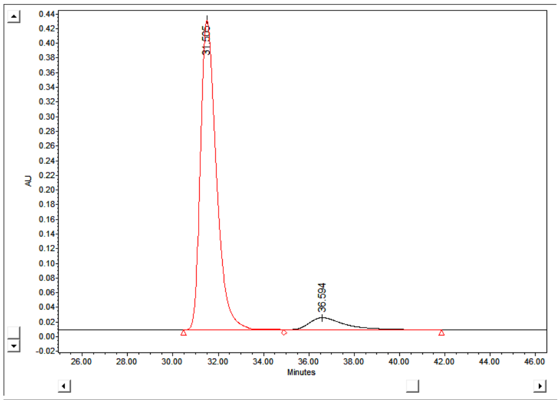

E	Name	Retention Time (min)	Area (μV*sec)	% Area	Height (μV)	Int Type	Amount	Units	Peak Type	Peak Codes
1		31.505	20507784	91.88	422411	bv			Unknown	
2		36.594	1811500	8.12	16619	vb			Unknown	

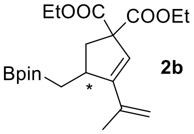

2b

Reaction time: 42 h. Isolated yield: 69 mg (88%). *ee* = 84%.

^{1}H NMR ($CDCl_3$, 400 MHz): δ 5.66 (s, 1H), 5.02 (br. s, 1H), 5.00 (br. s, 1H), 4.22 – 4.12 (m, 4H), 3.17 (m, 1H), 2.70 (dd, J = 13.8, 8.7 Hz, 1H), 2.25 (dd, J = 13.8, 3.0 Hz, 1H), 1.90 (br. s, 3H), 1.24 – 1.21 (m, 1H), 1.23 (t, J = 7.1 Hz, 6H), 1.23 (br. s, 6H), 1.22 (br. s, 6H), 0.75 (dd, J = 16.1, 11.6 Hz, 1H).

^{13}C NMR ($CDCl_3$, 100 MHz): δ 171.9, 171.6, 153.7, 138.0, 123.2, 115.3, 83.2 (2C), 65.7, 61.6, 61.5, 40.0, 39.6, 25.0 (2C), 24.8 (2C), 21.4, 18.1 (C–B), 14.1 (2C).

HRMS (ESI) m/z for $C_{21}H_{33}O_6BNa$ $[M+Na]^+$ calcd 415.2266, found 415.2246.

Retention time: 27.2 & 31.5 min, CHIRALCEL®-ID, *iso*-hexane/*iso*-propanol = 99/1, 25 ^{o}C, 0.3 mL/min.

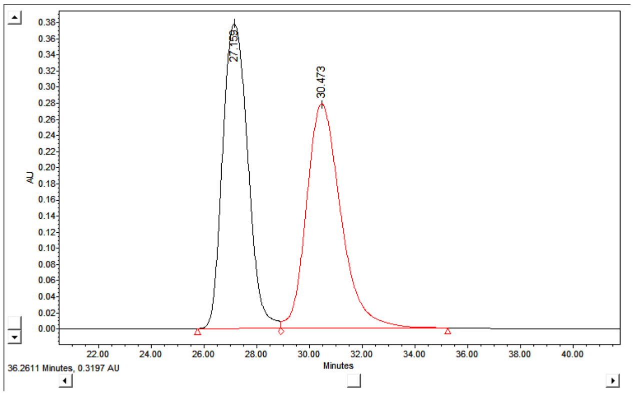

Name	Retention Time (min)	Area (µV*sec)	% Area	Height (µV)	Int Type	Amount	Units	Peak Type	Peak Codes
1	27.159	25111228	49.92	377802	BV			Unknown	
2	30.473	25195766	50.08	278013	Vb			Unknown	

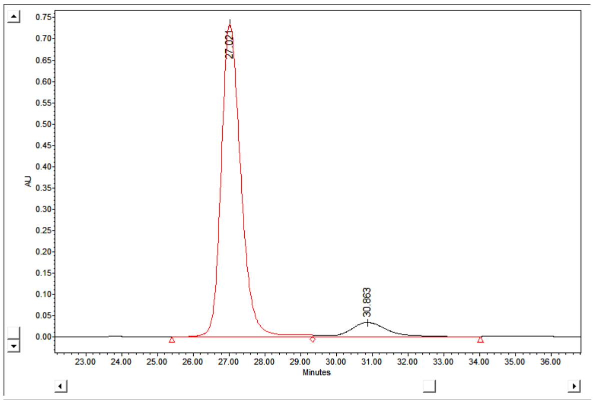

Name	Retention Time (min)	Area (µV*sec)	% Area	Height (µV)	Int Type	Amount	Units	Peak Type	Peak Codes
1	27.021	27841039	92.18	735264	bv			Unknown	
2	30.863	2363420	7.82	33107	vb			Unknown	

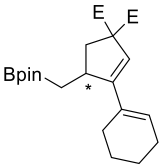

2c

Reaction time: 84 h. Isolated yield: 64 mg (79%). *ee* = 93%**.**

^{1}H NMR ($CDCl_3$, 400 MHz): δ 5.82 (m, 1H), 5.55 (s, 1H), 3.71 (s, 3H), 3.70 (s, 3H), 3.17 (m, 1H), 2.68 (dd, J = 13.8, 6.9 Hz, 1H), 2.27 (dd, J = 13.8, 2.9 Hz, 2H), 2.12 (m, 3H), 1.66 (m, 3H), 1.57 (m, 2H), 1.24 (s, 6H), 1.24 (s, 6H), 0.72 (dd, J = 16.1, 11.8 Hz, 1H).

^{13}C NMR ($CDCl_3$, 100 MHz): δ 172.5, 172.3, 154.4, 131.6, 127.7, 119.4, 83.2 (2C), 65.4, 52.8 (2C), 39.7, 39.6, 26.6, 25.9, 25.1 (2C), 24.8 (2C), 22.8, 22.3.

Retention time: 13.4 & 15.3 min, CHIRALCEL®-IC, *iso*-hexane/*iso*-propanol = 93/7, 30 ^{o}C, 0.5 mL/min.

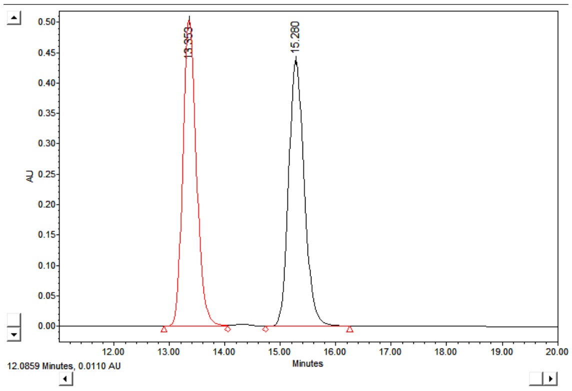

Name	Retention Time (min)	Area (µV*sec)	% Area	Height (µV)	Int Type	Amount	Units	Peak Type	Peak Codes
1	13.353	8542489	49.87	503269	BV			Unknown	
2	15.280	8588064	50.13	438445	VB			Unknown	

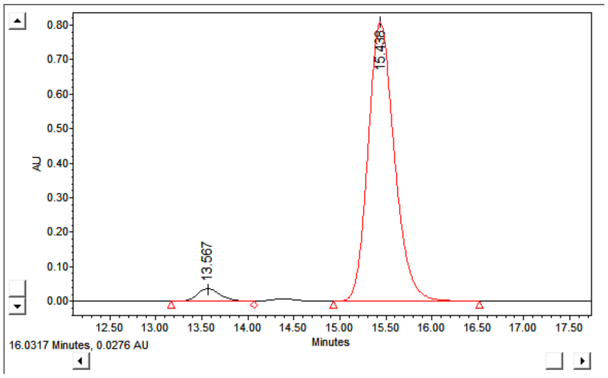

Name	Retention Time (min)	Area (µV*sec)	% Area	Height (µV)	Int Type	Amount	Units	Peak Type	Peak Codes
1	13.567	629701	3.74	36391	BV			Unknown	
2	15.438	16193850	96.26	807764	BB			Unknown	

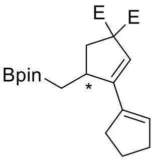

2d

Reaction time: 72 h. Isolated yield: 65 mg (83%). *ee* = 92%.

^{1}**H NMR** ($CDCl_3$, 400 MHz): δ 5.80 (m, 1H), 5.52 (s, 1H), 3.72 (s, 3H), 3.70 (s, 3H), 3.16 (m, 1H), 2.73 (dd, J = 13.8, 8.7 Hz, 1H), 2.55 – 2.35 (m, 4H), 2.26 (dd, J = 13.8, 3.5 Hz, 1H), 1.95 – 1.80 (m, 2H), 1.29 (dd, J = 16.0, 3.2 Hz, 1H), 1.24 (s, 6H), 1.24 (s, 6H), 0.77 (dd, J = 16.0, 11.6 Hz, 1H).

^{13}**C NMR** ($CDCl_3$, 100 MHz): δ 172.3, 172.2, 149.8, 138.0, 130.1, 122.1, 83.2 (2C), 65.5, 52.8 (2C), 40.8, 40.0, 33.5, 33.5, 25.1 (2C), 24.8 (2C), 23.1, 18.3 (C–B).

HRMS (ESI) m/z for $C_{21}H_{31}O_6BNa$ $[M+Na]^+$ calcd 413.2110, found 413.2130.

Retention time: 16.0 & 17.5 min, CHIRALCEL®-IC, *iso*-hexane/*iso*-propanol = 96/4, 30 °C, 0.5 mL/min.

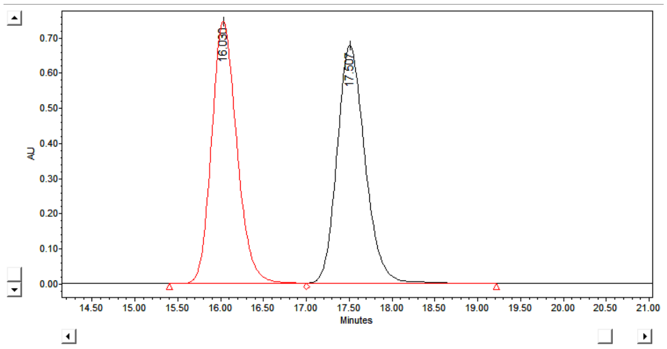

E	Name	Retention Time (min)	Area (μV*sec)	% Area	Height (μV)	Int Type	Amount	Units	Peak Type	Peak Codes
1		16.030	15249445	49.82	745704	bv			Unknown	
2		17.507	15361733	50.18	677266	vb			Unknown	

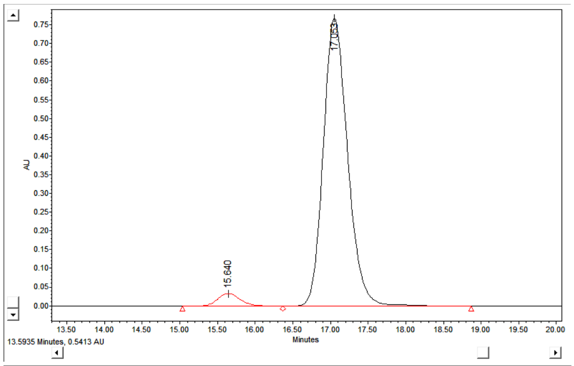

E	Name	Retention Time (min)	Area (μV*sec)	% Area	Height (μV)	Int Type	Amount	Units	Peak Type	Peak Codes
1		15.640	683501	3.83	33594	bv			Unknown	
2		17.053	17169766	96.17	768123	vb			Unknown	

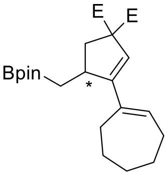

2e

Reaction time: 72 h. Isolated yield: 57 mg (68%). *ee* = 92%.

^{1}**H NMR** ($CDCl_3$, 400 MHz): δ 5.92 (t, J = 6.8 Hz, 1H), 5.56 (br. s, 1H), 3.72 (s, 3H), 3.70 (s, 3H), 3.17 – 3.11 (m, 1H), 2.74 (dd, J = 13.7, 6.8 Hz, 1H), 2.37 – 2.32 (m, 2H), 2.21 (dd, J = 13.7, 3.9 Hz, 1H), 2.21 – 2.17 (m, 2H), 1.75 – 1.72 (m, 2H), 1.50 – 1.46 (m, 4H), 1.24 (s, 6H), 1.24 (s, 6H), 1.22 (m, 1H), 0.69 (dd, J = 16.1, 11.6 Hz, 1H).

^{13}**C NMR** ($CDCl_3$, 100 MHz): δ 172.4, 172.4, 155.5, 139.2, 132.0, 119.8, 83.2 (2C), 65.3, 52.8, 52.8, 40.0, 40.0, 32.5, 31.0, 28.6, 26.8, 26.5, 25.1 (2C), 24.8 (2C).

HRMS (ESI) m/z for $C_{23}H_{35}O_6BNa$ $[M+Na]^+$ calcd 441.2423, found 441.2401.

Retention time: 15.0 & 16.5 min, CHIRALCEL®-IC, *iso*-hexane/*iso*-propanol = 95/5, 30 °C, 0.5 mL/min.

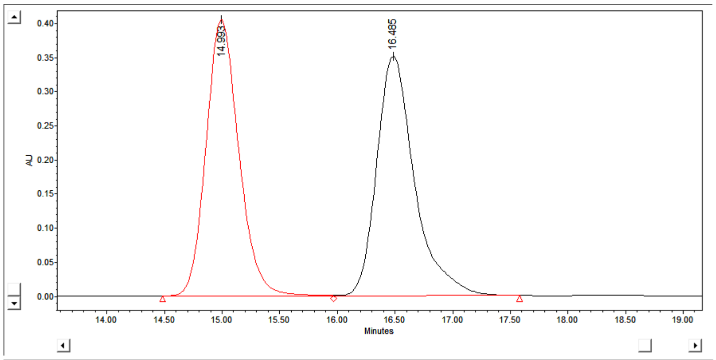

Name	Retention Time (min)	Area (µV*sec)	% Area	Height (µV)	Int Type	Amount	Units	Peak Type	Peak Codes
1	14.993	7983524	49.97	405083	BV			Unknown	
2	16.485	7991740	50.03	351353	VB			Unknown	

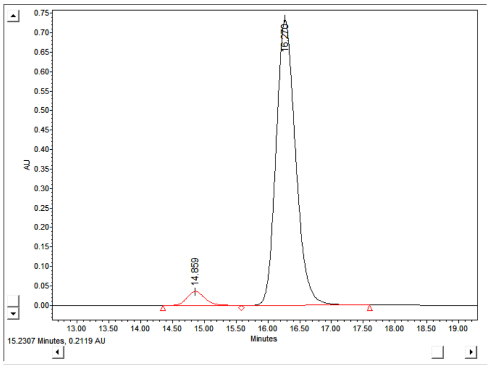

Name	Retention Time (min)	Area (µV*sec)	% Area	Height (µV)	Int Type	Amount	Units	Peak Type	Peak Codes
1	14.859	685604	4.21	35933	bv			Unknown	
2	16.270	15584910	95.79	735820	vb			Unknown	

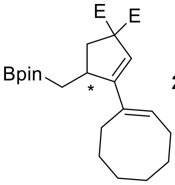

2f

Reaction time: 96 h. Isolated yield: 74 mg (86%). *ee* = 88%.

^{1}H NMR ($CDCl_3$, 400 MHz): δ 5.75 (t, J = 8.2 Hz, 1H), 5.61 (s, 1H), 3.71 (s, 3H), 3.70 (s, 3H), 3.20 – 3.14 (m, 1H), 2.69 (dd, J = 13.6, 8.4 Hz, 1H), 2.40 – 2.38 (m, 2H), 2.27 (dd, J = 13.6, 3.1 Hz, 1H), 2.22 – 2.16 (m, 2 H), 1.54 – 1.40 (m, 8H), 1.27 (dd, J = 16.2, 3.2 Hz, 1H), 1.25 (s, 6H), 1.24 (s, 6H), 0.69 (dd, J = 16.2, 11.9 Hz, 1H).

^{13}C NMR ($CDCl_3$, 100 MHz): δ 172.4, 172.3, 153.6, 135.0, 130.4, 120.3, 83.2 (2C), 65.3, 52.8 (2C), 39.7, 39.5, 30.3, 29.0, 27.2, 27.0, 26.9, 26.2, 25.1 (2C), 24.8 (2C), 18.2 (C–B).

HRMS (ESI) m/z for $C_{24}H_{37}O_6BNa$ $[M+Na]^+$ calcd 455.2580 , found 455.2601.

Retention time: 14.9 & 17.3 min, CHIRALCEL®-IC, *iso*-hexane/*iso*-propanol = 95/5, 30 ^{o}C, 0.5 mL/min.

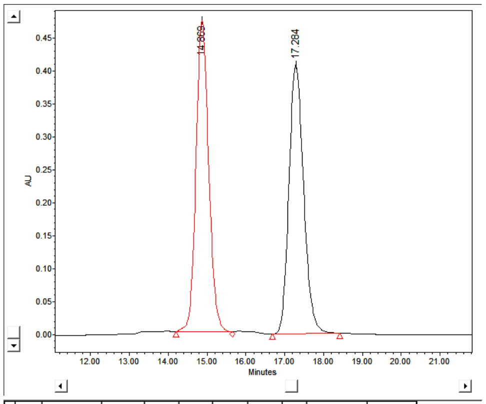

Name	Retention Time (min)	Area (µV*sec)	% Area	Height (µV)	Int Type	Amount	Units	Peak Type	Peak Codes
1	14.869	10725020	50.00	471966	bV			Unknown	
2	17.284	10723292	50.00	408477	BB			Unknown	

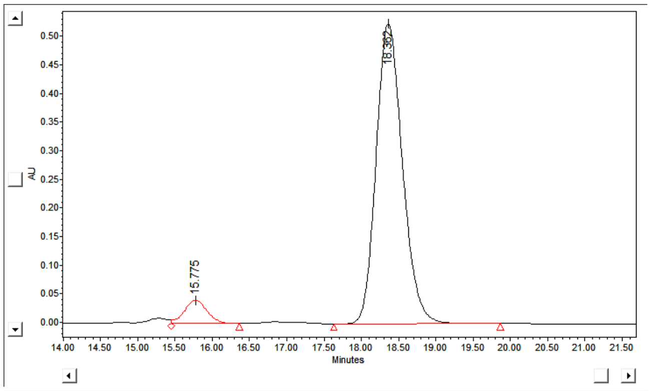

Name	Retention Time (min)	Area (µV*sec)	% Area	Height (µV)	Int Type	Amount	Units	Peak Type	Peak Codes
1	15.775	834806	6.10	40232	vb			Unknown	
2	18.362	12851874	93.90	524056	bb			Unknown	

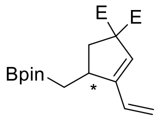

2g

Reaction time: 96 h. Isolated yield: 24 mg (34%). *ee* = 51%.

^{1}H NMR ($CDCl_3$, 400 MHz): δ 6.37 (dd, J = 17.8, 11.0 Hz, 1H), 5.68 (br. s, 1H), 5.31 (dd, J = 17.8, 0.9 Hz, 1H), 5.20 (dd, J = 11.0, 1.2 Hz, 1H), 3.73 (s, 3H), 3.71 (s, 3H), 3.15 (m, 1H), 2.77 (dd, J = 13.8, 8.5 Hz, 1H), 2.23 (dd, J = 13.8, 3.8 Hz, 1H), 1.27 (dd, J = 16.0, 3.6 Hz, 1H), 1.25 (s, 6H), 1.24 (s, 6H), 0.75 (dd, J = 16.0, 11.4 Hz, 1H).

^{13}C NMR ($CDCl_3$, 100 MHz): δ 172.0, 171.9, 151.7, 131.4, 125.3, 117.5, 83.3 (2C), 65.3, 52.9 (2C), 40.0, 39.4, 25.1 (2C), 24.8 (2C).

HRMS (ESI) m/z for $C_{18}H_{27}O_6BNa$ $[M+Na]^+$ calcd 373.1796, found 373.1812.

Retention time: 36.1 & 40.1 min, CHIRALCEL®-ID, *iso*-hexane/*iso*-propanol = 99/1, 30 °C, 0.3 mL/min.

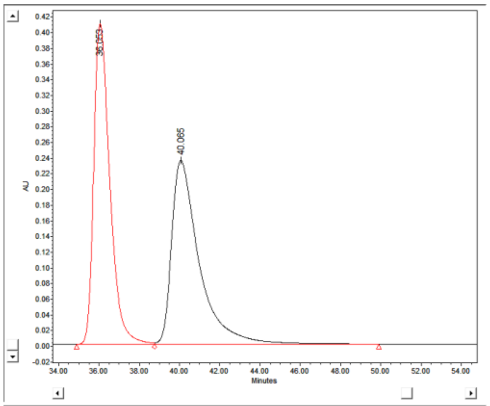

E	Name	Retention Time (min)	Area (μV*sec)	% Area	Height (μV)	Int Type	Amount	Units	Peak Type	Peak Codes
1		36.053	22730387	50.43	408667	bv			Unknown	
2		40.065	22346316	49.57	234790	vb			Unknown	

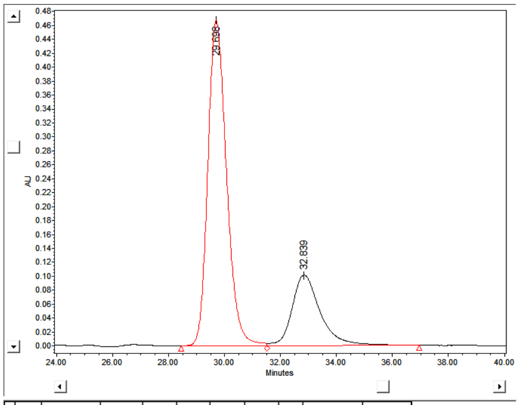

E	Name	Retention Time (min)	Area (μV*sec)	% Area	Height (μV)	Int Type	Amount	Units	Peak Type	Peak Codes
1		29.698	21662941	75.42	466662	bv			Unknown	
2		32.839	7061801	24.58	101272	vb			Unknown	

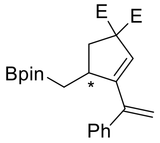

2h

Reaction time: 48 h. Isolated yield: 64 mg (75%). *ee* = 71%.

^{1}H NMR ($CDCl_3$, 400 MHz): δ 7.36 – 7.27 (m, 5H), 5.60 (d, J = 1.4 Hz, 1H), 5.33 (d, J = 1.2 Hz, 1H), 5.26 (d, J = 1.2 Hz, 1H), 3.74 (s, 3H), 3.69 (s, 3H), 3.27 (m, 1H), 2.88 (dd, J = 13.8, 8.4 Hz, 1H), 2.22 (dd, J = 13.8, 4.9 Hz, 1H), 1.24 (dd, J = 16.1, 3.7 Hz, 1H), 1.23 (s, 12H), 0.78 (dd, J = 16.1, 11.2 Hz, 1H).

^{13}C NMR ($CDCl_3$, 100 MHz): δ 172.0, 171.8, 153.4, 144.3, 140.9, 128.2 (2C), 128.0 (2C), 127.6, 126.2, 116.0, 83.2 (2C), 65.5, 52.8, 52.8, 40.8, 39.8, 25.0 (2C), 24.8 (2C), 17.1 (C–B).

Retention time: 15.4 & 22.2 min, CHIRALCEL®-OJ, *iso*-hexane/*iso*-propanol = 98/2, 30 °C, 0.5 mL/min.

HRMS (ESI) m/z for $C_{24}H_{31}O_6BNa$ $[M+Na]^+$ calcd 449.2110, found 449.2093.

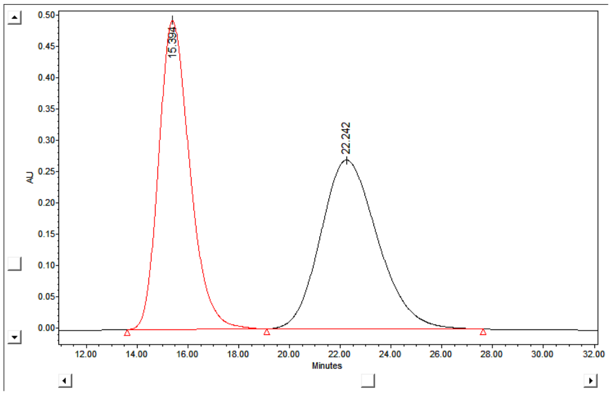

E	Name	Retention Time (min)	Area (µV*sec)	% Area	Height (µV)	Int Type	Amount	Units	Peak Type	Peak Codes
1		15.394	42034918	49.98	492712	BB			Unknown	
2		22.242	42071530	50.02	269491	Bb			Unknown	

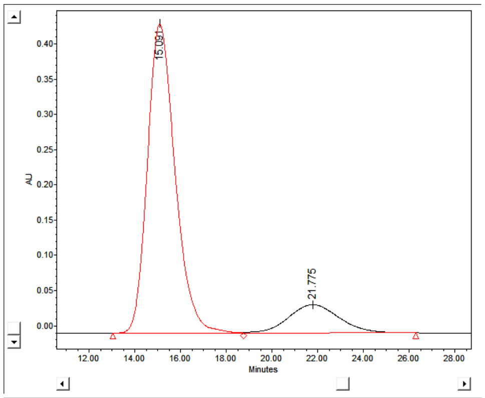

E	Name	Retention Time (min)	Area (µV*sec)	% Area	Height (µV)	Int Type	Amount	Units	Peak Type	Peak Codes
1		15.091	36855021	85.66	438724	bv			Unknown	
2		21.775	6168403	14.34	40088	vb			Unknown	

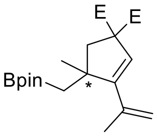

2i

Reaction time: 96 h. Isolated yield: 64 mg (79%). *ee* = 75%.

^{1}H NMR ($CDCl_3$, 400 MHz): δ 5.64 (s, 1H), 5.14 (br. s, 1H), 5.02 (m, 1H), 3.71 (s, 3H), 3.71 (s, 3H), 2.62 (d, J = 13.8 Hz, 1H), 2.46 (d, J = 13.8 Hz, 1H), 1.89 (br. s, 3H), 1.31 (s, 3H), 1.28 (d, J = 15.4 Hz, 1H), 1.20 (s, 12H), 1.07 (d, J = 15.4 Hz, 1H).

^{13}C NMR ($CDCl_3$, 100 MHz): δ 172.1, 172.0, 156.0, 138.1, 123.8, 115.2, 83.0 (2C), 63.5, 52.9, 52.7, 47.9, 47.8, 29.2, 24.9 (2C), 24.9 (2C), 23.7.

Retention time: 29.4 & 35.7 min, CHIRALCEL®-OJ, *iso*-hexane/*iso*-propanol = 99/1, 25 °C, 0.3 mL/min.

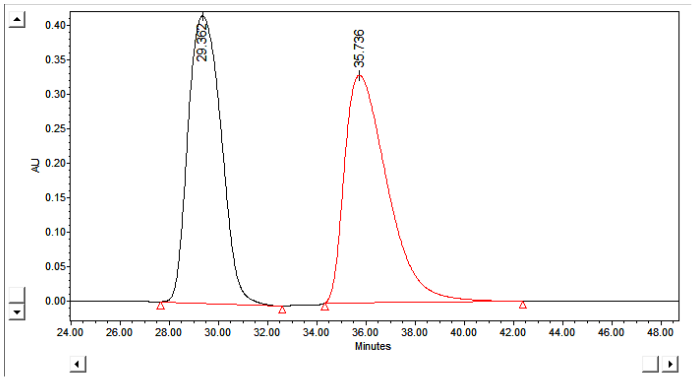

E	Name	Retention Time (min)	Area (µV*sec)	% Area	Height (µV)	Int Type	Amount	Units	Peak Type	Peak Codes
1		29.362	38894854	49.45	415826	bb			Unknown	
2		35.736	39766716	50.55	329293	bb			Unknown	

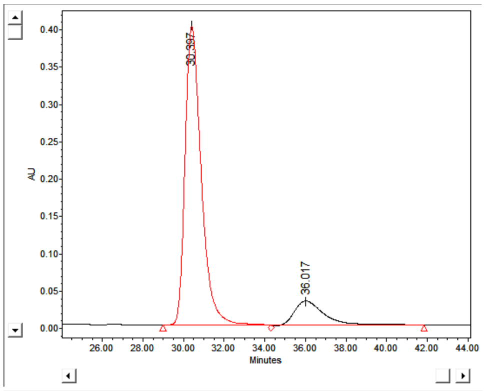

E	Name	Retention Time (min)	Area (µV*sec)	% Area	Height (µV)	Int Type	Amount	Units	Peak Type	Peak Codes
1		30.397	22093431	87.53	400089	bv			Unknown	
2		36.017	3148422	12.47	31760	vb			Unknown	

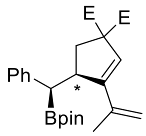

2k

Reaction time: 86 h. Isolated yield: 9 mg (10%). *ee* = 68%.

^{1}H NMR ($CDCl_3$, 400 MHz): δ 7.25 – 7.21 (m, 4H), 7.14 (m, 1H), 5.77 (d, J = 1.3 Hz, 1H), 4.94 (m, 1H), 4.91 (br. s, 1H), 3.77 (s, 3H), 3.67 (s, 3H), 3.48 (m, 1H), 2.92 (d, J = 6.0 Hz, 1H), 2.70 (dd, J = 14.1, 8.3 Hz, 1H), 2.62 (dd, J = 14.1, 5.5 Hz, 1H), 1.86 (br. s, 3H), 1.21 (s, 6H), 1.18 (s, 6H).

^{13}C NMR ($CDCl_3$, 100 MHz): δ 171.9, 171.8, 152.2, 141.9, 138.6, 129.5 (2C), 128.4 (2C), 125.7, 124.2, 115.2, 83.4 (2C), 65.3, 52.9, 52.6, 48.1, 36.5, 24.9 (2C), 25.7 (2C), 21.6.

HRMS (ESI) m/z for $C_{25}H_{33}O_6BNa$ $[M+Na]^+$ calcd 463.2267, found 463.2273.

Retention time: 23.7 & 36.9 min, CHIRALCEL®-IA, *iso*-hexane/*iso*-propanol = 99/1, 25 °C, 0.3 mL/min.

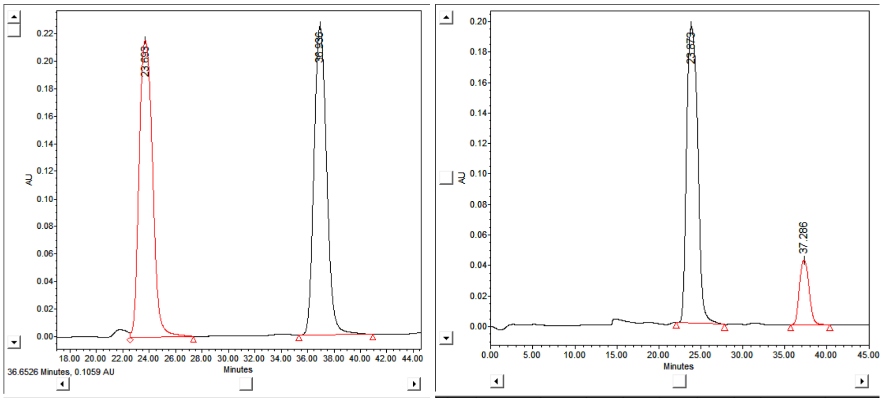

	Name	Retention Time (min)	Area (µV*sec)	% Area	Height (µV)	Int Type	Amount	Units	Peak Type	Peak Codes
1		23.693	15141269	50.51	214562	vb			Unknown	
2		36.936	14833796	49.49	223469	bb			Unknown	

	Name	Retention Time (min)	Area (µV*sec)	% Area	Height (µV)	Int Type	Amount	Units	Peak Type	Peak Codes
1		23.873	17815711	84.11	194587	bb			Unknown	
2		37.286	3365871	15.89	42601	bb			Unknown	

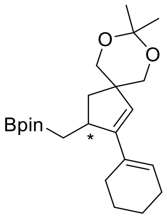

2m

Reaction time: 108 h. Isolated yield: 59 mg (76%). *ee* = 93%.

^{1}H NMR ($CDCl_3$, 400 MHz): δ 5.75 (t, J = 3.8 Hz, 1H), 5.52 (br. s, 1H), 3.76 (d, J = 11.2 Hz, 1H), 3.68 (d, J = 11.2 Hz, 1H), 3.63 (d, J = 11.2 Hz, 1H), 3.54 (d, J = 11.2 Hz, 1H), 3.14 – 3.09 (m, 1H), 2.28 – 2.21 (m, 1H), 2.15 – 2.06 (m, 3H), 2.04 (dd, J = 13.8, 9.1 Hz, 1H), 1.67 – 1.55 (m, 4H), 1.52 (dd, J = 13.8, 3.5 Hz, 1H), 1.43 (s, 3H), 1.42 (s, 3H), 1.32 (dd, J = 15.9, 3.0 Hz, 1H), 1.25 (s, 6H), 1.23 (s, 6H), 0.64 (dd, J = 15.9, 11.5 Hz, 1H).

^{13}C NMR ($CDCl_3$, 100 MHz): δ 151.6, 132.2, 125.9, 124.7, 97.5, 83.1 (2C), 69.8, 69.6, 48.0, 40.0, 38.9, 26.7, 25.8, 25.1 (2C), 24.8 (2C), 24.4, 23.6, 22.9, 22.4, 20.1 (C–B).

HRMS (ESI) m/z for $C_{23}H_{37}BO_4Na$ $[M+Na]^+$ calcd 411.2677, found 411.2691.

Retention time: 14.3 & 15.3 min, CHIRALCEL®-IA, *iso*-hexane/*iso*-propanol = 98/2, 25 °C, 0.3 mL/min.

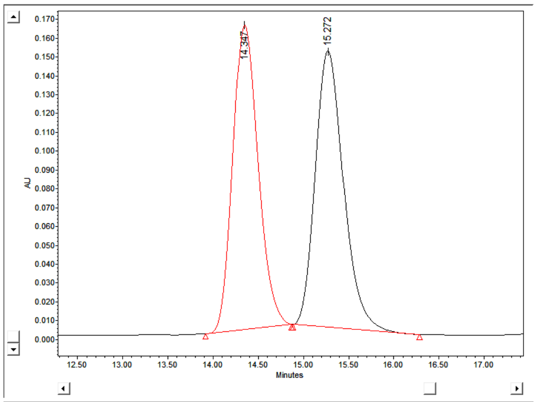

E	Name	Retention Time (min)	Area (μV*sec)	% Area	Height (μV)	Int Type	Amount	Units	Peak Type	Peak Codes
1		14.347	3154860	49.68	161661	bb			Unknown	
2		15.272	3195590	50.32	146785	bb			Unknown	

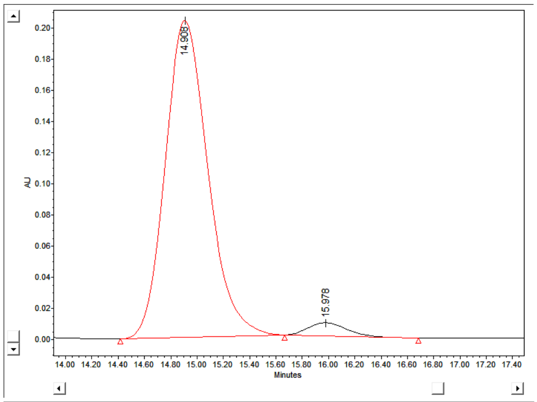

E	Name	Retention Time (min)	Area (μV*sec)	% Area	Height (μV)	Int Type	Amount	Units	Peak Type	Peak Codes
1		14.908	4575933	96.41	203150	bb			Unknown	
2		15.978	170156	3.59	8400	bb			Unknown	

(1) Black, D. K.; Landor, S. R.; Patel, A. N.; Whiter, P. F. *Tetrahedron Lett.* **1963**, *4*, 483.

(2) Franzén, J.; Bäckvall, J.-E. *J. Am. Chem. Soc.* **2003**, *125*, 6056

(3) Deng, Y.; Bäckvall, J.-E. *Angew. Chem. Int. Ed.* **2013**, *52*, 3217.

(4) (a) Hatano, M.; Ikeno, T.; Matsumura, T.; Torii, S.; Ishihara, K. *Adv. Synth. Catal.* **2008**, *350*, 1776–1780. (b) Storer, R. I.; Carrera, D. E.; Ni, Y.; Macmillan, D. W. C. *J. Am. Chem. Soc.* **2006**, *128*, 84–86. (c) Holloway, C. A.; Muratore, M. E.; Storer, R.; Dixon, D. J. *Org. Lett.* **2010**, *12*, 4720–4723. (d) Itoh, J.; Fuchibe, K.; Akiyama, T. *Angew. Chem. Int. Ed.* **2006**, *45*, 4796–4798. (e) Yamanaka, M.; Itoh, J.; Fuchibe, K.; Akiyama, T. *J. Am. Chem. Soc.* **2007**, *129*, 6756–6764.(f) Seayad, J.; Seayad, A. M.; List, B. *J. Am. Chem. Soc.* **2006**, *128*, 1086–1087. (g) Huang, Y.-Y.; Chakrabarti, A.; Morita, N.; Schneider, U.; Kobayashi, S. *Angew. Chem. Int. Ed.* **2011**, *50*, 11121–11124.

(5) (a) Alexander, J. B.; La, D. S.; Cefalo, D. R.; Hoveyda, A. H.; Schrock, R. R. *J. Am. Chem. Soc.* **1998**, *120*, 4041–4042. (b) Schrock, R. R.; Hoveyda, A. H. WO2000002834 A1, **2000**.

(6) Gutierrez, E. G.; Moorhead, E. J.; Smith, E. H.; Lin, V.; Ackerman, L. K. G.; Knezevic, C. E.; Sun, V.; Grant, S.; Wenzel, A. G. *Eur. J. Org. Chem.* **2010**, 3027–3031.

(7) Hua, Z.; Vassar, V. C.; Ojima, I. *Org. Lett.* **2003**, *5*, 3831–3834.

(8) Nakashima, D.; Yamamoto, H. *J. Am. Chem. Soc.* **2006**, *128*, 9626–9627.

(9) Persson, A. K. Å.; Jiang, T.; Johnson, M. T.; Bäckvall, J.-E. *Angew. Chem. Int. Ed.* **2011**, *50*, 6155–6159.

```
NAME     TJ-20130910-D-679-diEt-prod
EXPNO                     10
PROCNO                     1
Date_               20130910
Time                   16.51
INSTRUM                spect
PROBHD   5 mm PABBO BB-
PULPROG                 zg30
TD                     65536
SOLVENT                CDCl3
NS                        16
DS                         2
SWH                 8223.685 Hz
FIDRES              0.125483 Hz
AQ                 3.9846387 sec
RG                       724
DW                    60.800 usec
DE                      6.50 usec
TE                     297.7 K
D1                1.00000000 sec
TD0                        1

======== CHANNEL f1 ========
NUC1                      1H
P1                      8.90 usec
PL1                    -4.00 dB
PL1W             24.73352814 W
SFO1             400.1324710 MHz
SI                     32768
SF               400.1300095 MHz
WDW                       EM
SSB                        0
LB                      0.30 Hz
GB                         0
PC                      1.00
```

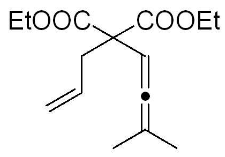

1b

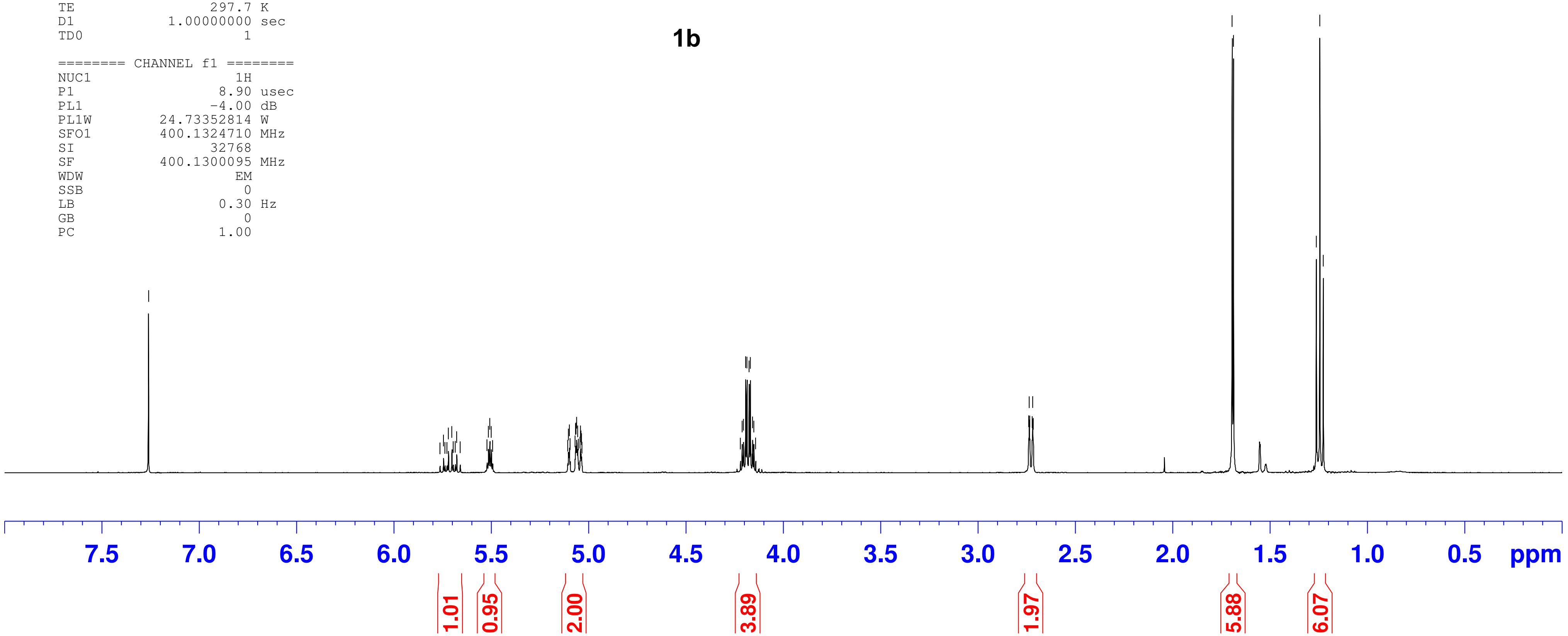

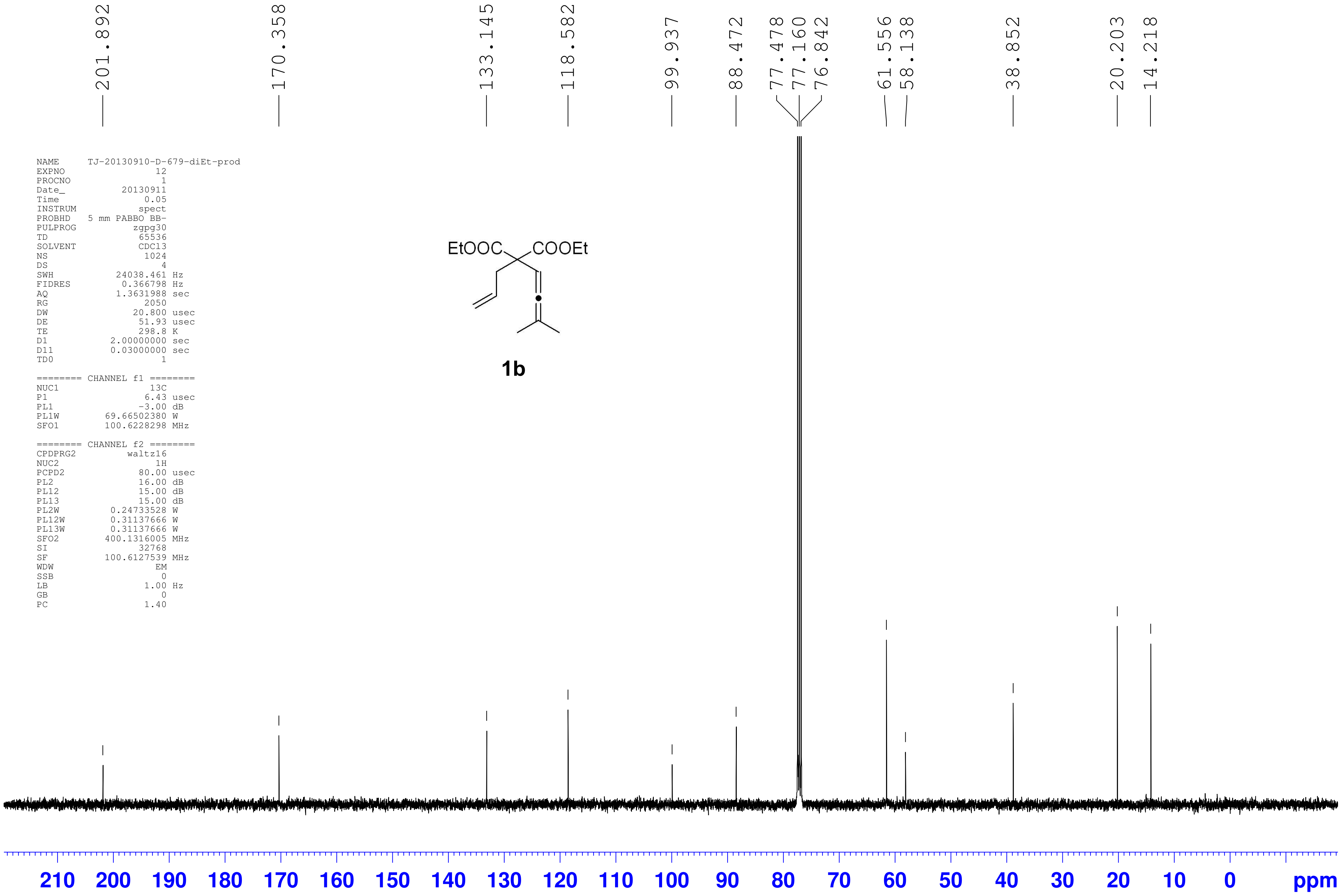
201.892
170.358
133.145
118.582
99.937
88.472
77.478
77.160
76.842
61.556
58.138
38.852
20.203
14.218
NAME TJ-20130910-D-679-diEt-prod
EXPNO 12
PROCNO 1
Date_ 20130911
Time 0.05
INSTRUM spect
PROBHD 5 mm PABBO BB-
PULPROG zgpg30
TD 65536
SOLVENT CDCl3
NS 1024
DS 4
SWH 24038.461 Hz
FIDRES 0.366798 Hz
AQ 1.3631988 sec
RG 2050
DW 20.800 usec
DE 51.93 usec
TE 298.8 K
D1 2.00000000 sec
D11 0.03000000 sec
TD0 1
======== CHANNEL f1 ========
NUC1 13C
P1 6.43 usec
PL1 -3.00 dB
PL1W 69.66502380 W
SFO1 100.6228298 MHz
======== CHANNEL f2 ========
CPDPRG2 waltz16
NUC2 1H
PCPD2 80.00 usec
PL2 16.00 dB
PL12 15.00 dB
PL13 15.00 dB
PL2W 0.24733528 W
PL12W 0.31137666 W
PL13W 0.31137666 W
SFO2 400.1316005 MHz
SI 32768
SF 100.6127539 MHz
WDW EM
SSB 0
LB 1.00 Hz
GB 0
PC 1.40
210 200 190 180 170 160 150 140 130 120 110 100 90 80 70 60 50 40 30 20 10 0 ppm
EtOOC
COOEt

1b

```
NAME      TJ-20140808-E-988-prod
EXPNO                   10
PROCNO                   1
Date_             20140808
Time                 21.48
INSTRUM              spect
PROBHD    5 mm PABBO BB/
PULPROG               zg30
TD                   65536
SOLVENT              CDCl3
NS                      16
DS                       2
SWH               8012.820 Hz
FIDRES            0.122266 Hz
AQ               4.0894966 sec
RG                  171.39
DW                  62.400 usec
DE                    6.50 usec
TE                   300.7 K
D1              1.00000000 sec
TD0                      1

======== CHANNEL f1 ========
SFO1           400.2424716 MHz
NUC1                    1H
P1                    9.40 usec
SI                   65536
SF             400.2400096 MHz
WDW                     EM
SSB                      0
LB                    0.30 Hz
GB                       0
PC                    1.00
```

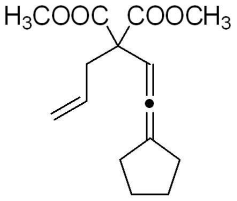

1d

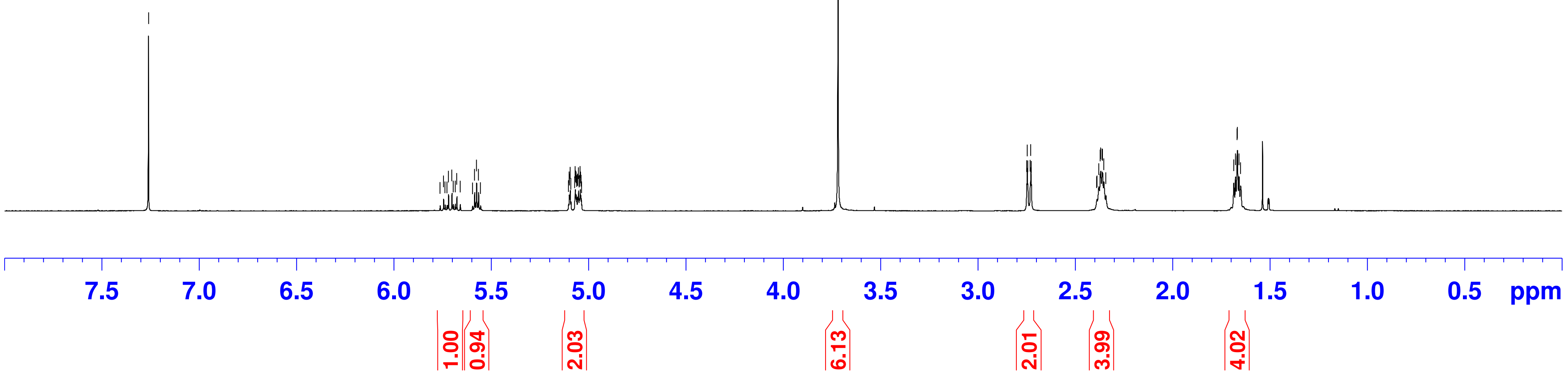

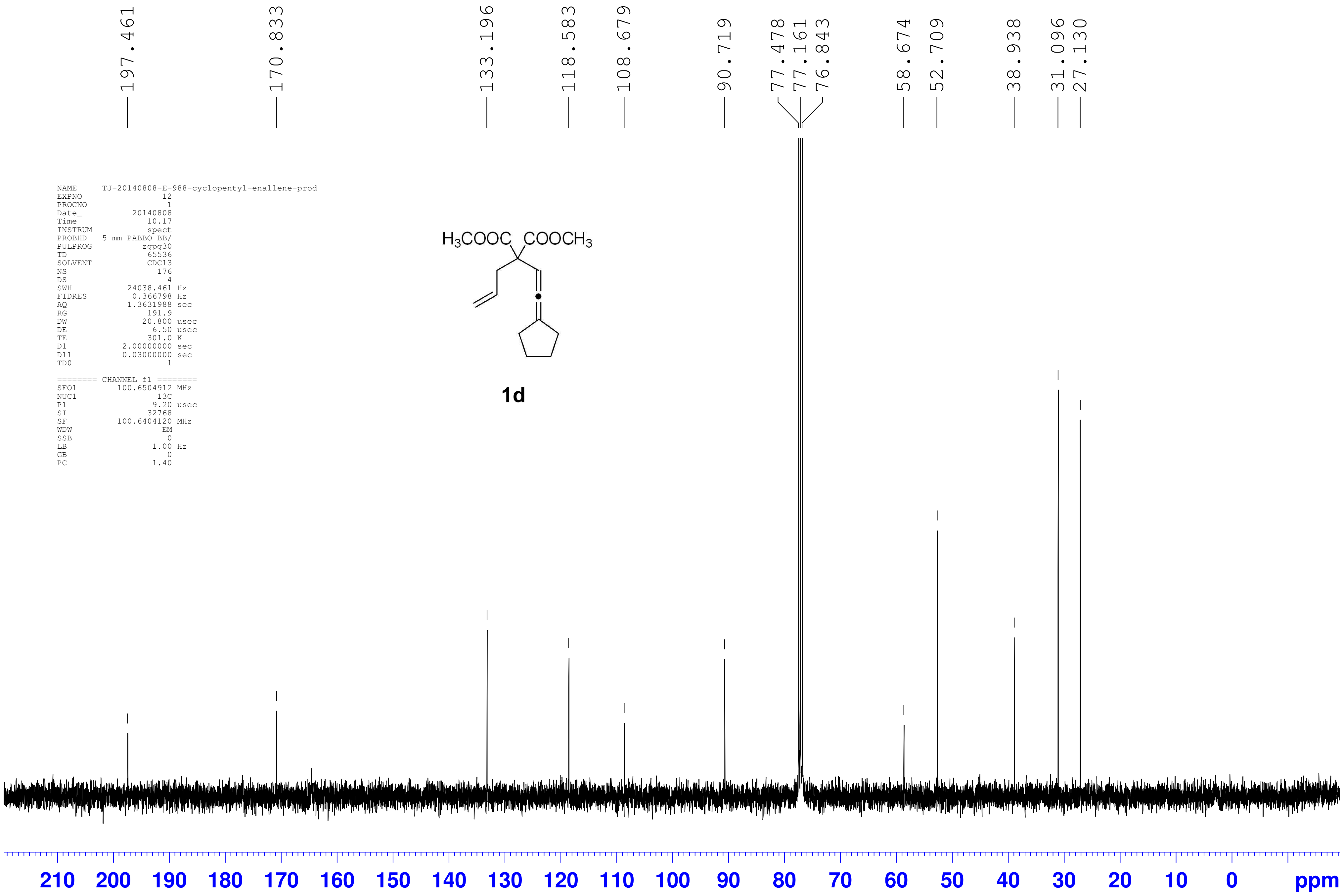
197.461
170.833
133.196
118.583
108.679
90.719
77.478
77.161
76.843
58.674
52.709
38.938
31.096
27.130
NAME TJ-20140808-E-988-cyclopentyl-enallene-prod
EXPNO 12
PROCNO 1
Date_ 20140808
Time 10.17
INSTRUM spect
PROBHD 5 mm PABBO BB/
PULPROG zgpg30
TD 65536
SOLVENT CDCl3
NS 176
DS 4
SWH 24038.461 Hz
FIDRES 0.366798 Hz
AQ 1.3631988 sec
RG 191.9
DW 20.800 usec
DE 6.50 usec
TE 301.0 K
D1 2.00000000 sec
D11 0.03000000 sec
TD0 1
======== CHANNEL f1 ========
SFO1 100.6504912 MHz
NUC1 13C
P1 9.20 usec
SI 32768
SF 100.6404120 MHz
WDW EM
SSB 0
LB 1.00 Hz
GB 0
PC 1.40
H3COOC COOCH3
1d
210 200 190 180 170 160 150 140 130 120 110 100 90 80 70 60 50 40 30 20 10 0 ppm

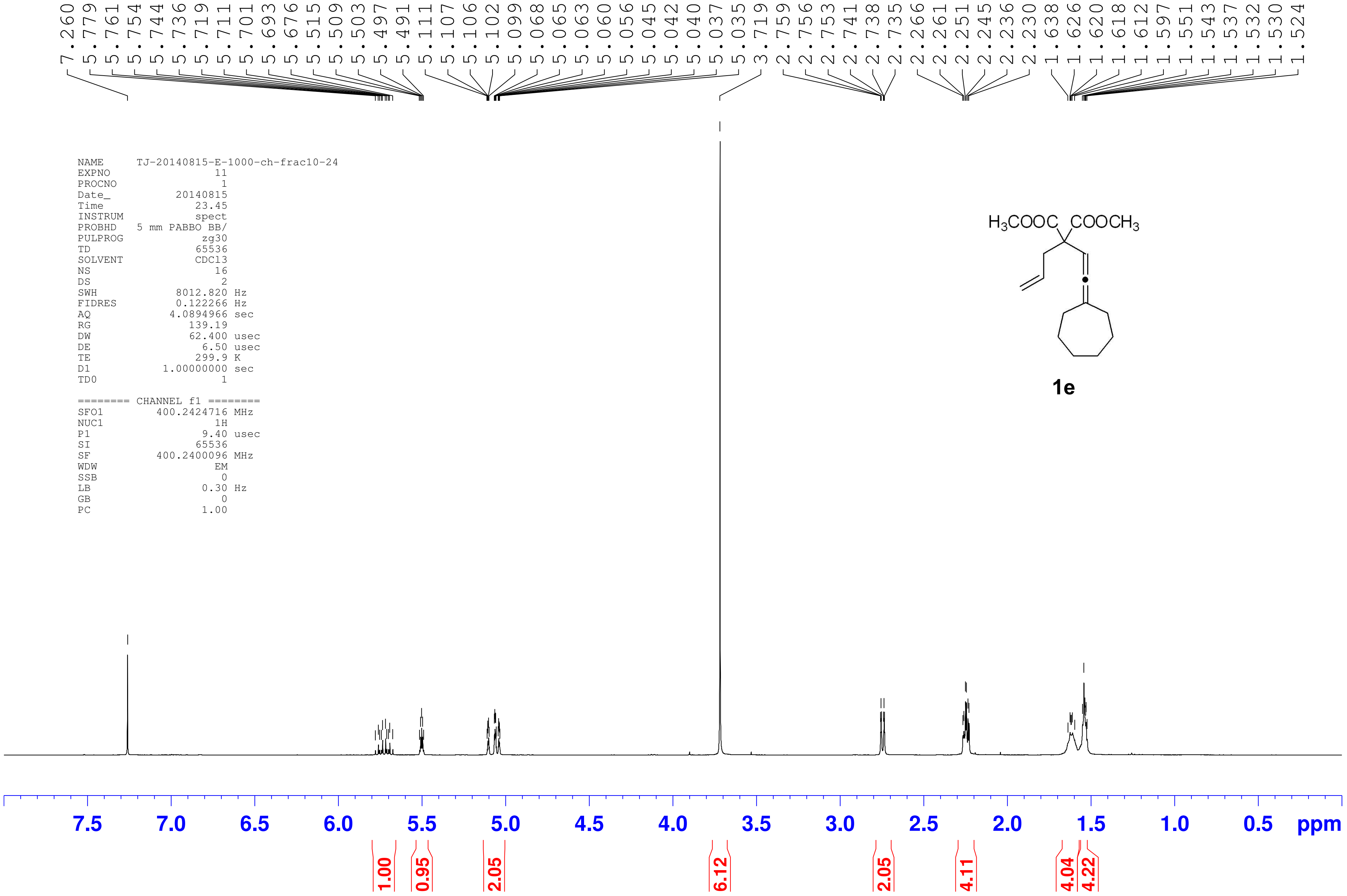
7.260
5.779
5.761
5.754
5.744
5.736
5.719
5.711
5.701
5.693
5.676
5.515
5.509
5.503
5.497
5.491
5.111
5.107
5.106
5.102
5.099
5.068
5.065
5.063
5.060
5.056
5.045
5.042
5.040
5.037
5.035
3.719
2.759
2.756
2.753
2.741
2.738
2.735
2.266
2.261
2.251
2.245
2.236
2.230
1.638
1.626
1.620
1.618
1.612
1.597
1.551
1.543
1.537
1.532
1.530
1.524
NAME TJ-20140815-E-1000-ch-frac10-24
EXPNO 11
PROCNO 1
Date_ 20140815
Time 23.45
INSTRUM spect
PROBHD 5 mm PABBO BB/
PULPROG zg30
TD 65536
SOLVENT CDCl3
NS 16
DS 2
SWH 8012.820 Hz
FIDRES 0.122266 Hz
AQ 4.0894966 sec
RG 139.19
DW 62.400 usec
DE 6.50 usec
TE 299.9 K
D1 1.00000000 sec
TD0 1
======== CHANNEL f1 ========
SFO1 400.2424716 MHz
NUC1 1H
P1 9.40 usec
SI 65536
SF 400.2400096 MHz
WDW EM
SSB 0
LB 0.30 Hz
GB 0
PC 1.00
H3COOC
COOCH3
1e
7.5
7.0
6.5
6.0
5.5
5.0
4.5
4.0
3.5
3.0
2.5
2.0
1.5
1.0
0.5
ppm
1.00
0.95
2.05
6.12
2.05
4.11
4.04
4.22

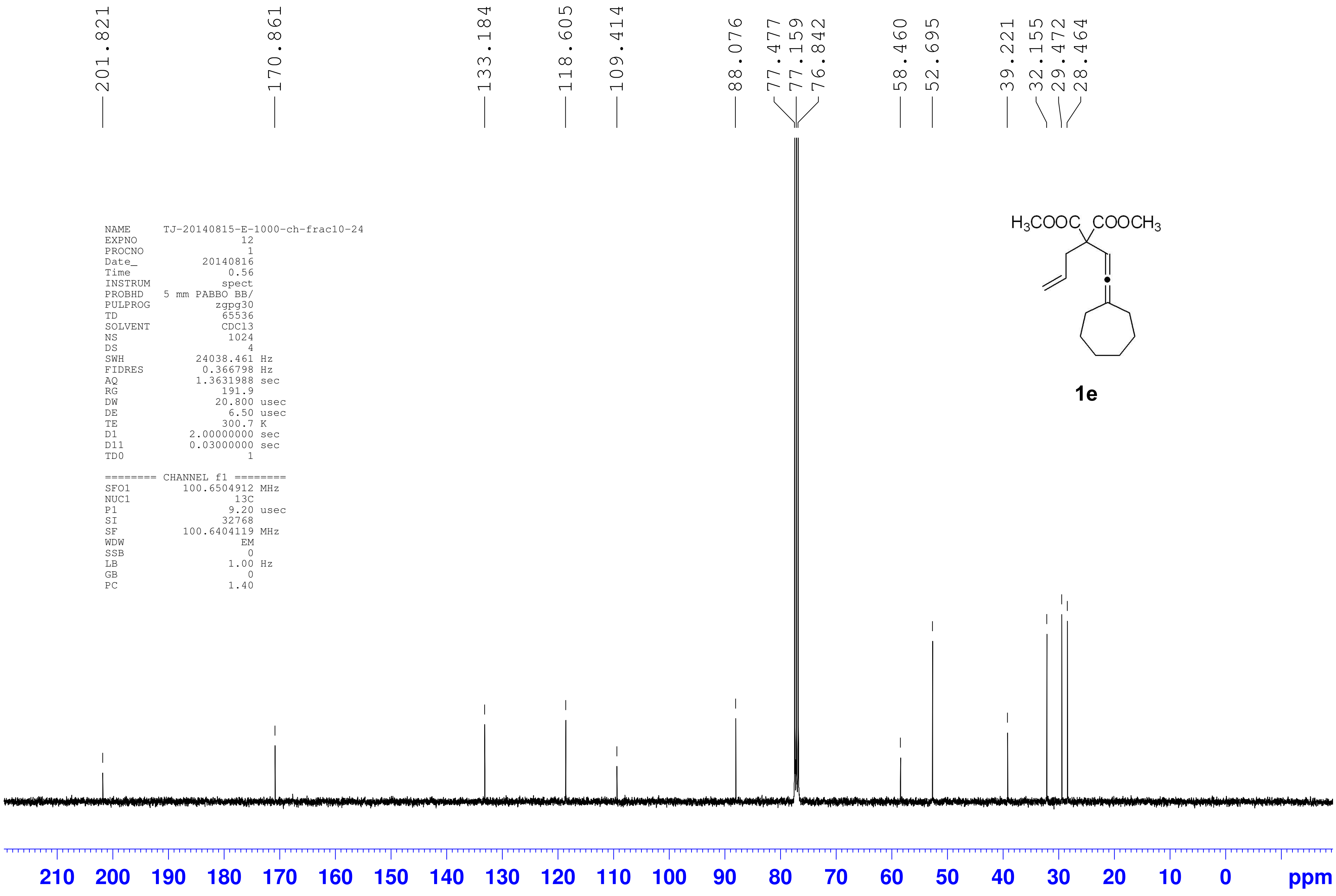

NAME TJ-20140815-E-1000-ch-frac10-24
EXPNO 12
PROCNO 1
Date_ 20140816
Time 0.56
INSTRUM spect
PROBHD 5 mm PABBO BB/
PULPROG zgpg30
TD 65536
SOLVENT CDCl3
NS 1024
DS 4
SWH 24038.461 Hz
FIDRES 0.366798 Hz
AQ 1.3631988 sec
RG 191.9
DW 20.800 usec
DE 6.50 usec
TE 300.7 K
D1 2.00000000 sec
D11 0.03000000 sec
TD0 1

======== CHANNEL f1 ========
SFO1 100.6504912 MHz
NUC1 13C
P1 9.20 usec
SI 32768
SF 100.6404119 MHz
WDW EM
SSB 0
LB 1.00 Hz
GB 0
PC 1.40

H_3COOC $COOCH_3$

1e

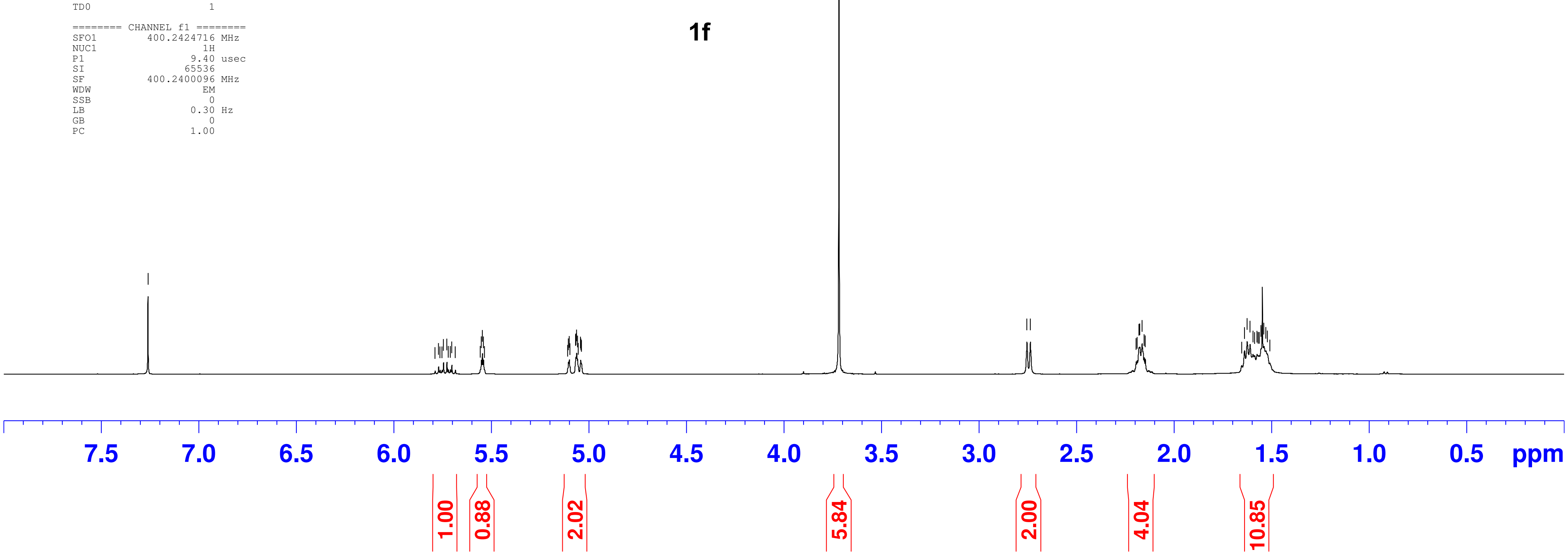


```
NAME     TJ-20140901-E-1014-co-enallene-frac16-26
EXPNO              12
PROCNO              1
Date_        20140901
Time            20.08
INSTRUM         spect
PROBHD   5 mm PABBO BB/
PULPROG          zg30
TD              65536
SOLVENT         CDCl3
NS                 16
DS                  2
SWH          8012.820 Hz
FIDRES       0.122266 Hz
AQ          4.0894966 sec
RG             139.19
DW             62.400 usec
DE               6.50 usec
TE              298.2 K
D1         1.00000000 sec
TD0                 1

======== CHANNEL f1 ========
SFO1      400.2424716 MHz
NUC1               1H
P1               9.40 usec
SI              65536
SF        400.2400096 MHz
WDW                EM
SSB                 0
LB               0.30 Hz
GB                  0
PC               1.00
```

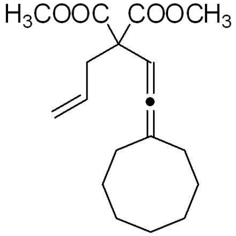

1f

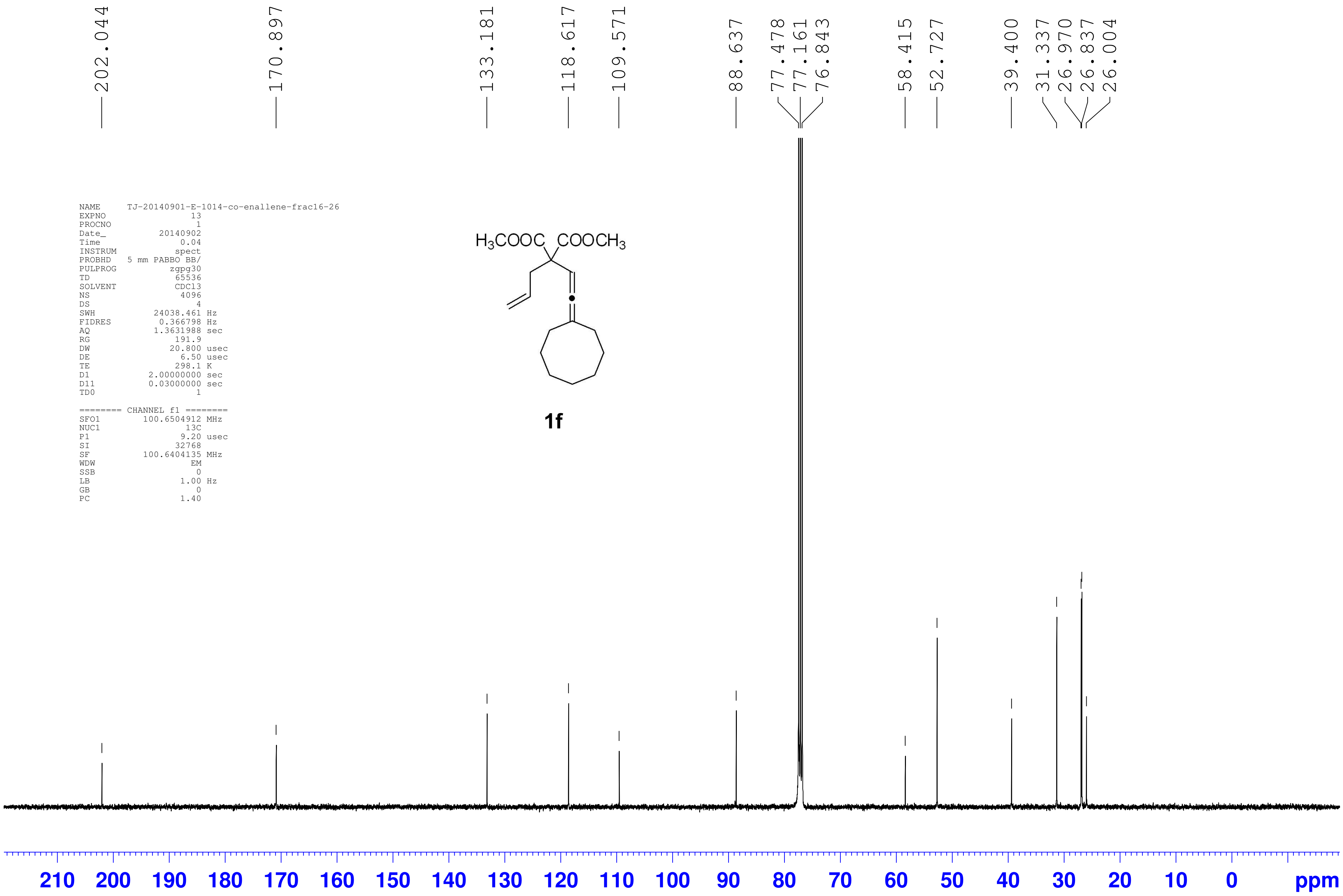
202.044
170.897
133.181
118.617
109.571
88.637
77.478
77.161
76.843
58.415
52.727
39.400
31.337
26.970
26.837
26.004
NAME TJ-20140901-E-1014-co-enallene-frac16-26
EXPNO 13
PROCNO 1
Date_ 20140902
Time 0.04
INSTRUM spect
PROBHD 5 mm PABBO BB/
PULPROG zgpg30
TD 65536
SOLVENT CDCl3
NS 4096
DS 4
SWH 24038.461 Hz
FIDRES 0.366798 Hz
AQ 1.3631988 sec
RG 191.9
DW 20.800 usec
DE 6.50 usec
TE 298.1 K
D1 2.00000000 sec
D11 0.03000000 sec
TD0 1
======== CHANNEL f1 ========
SFO1 100.6504912 MHz
NUC1 13C
P1 9.20 usec
SI 32768
SF 100.6404135 MHz
WDW EM
SSB 0
LB 1.00 Hz
GB 0
PC 1.40
H3COOC COOCH3
1f
210 200 190 180 170 160 150 140 130 120 110 100 90 80 70 60 50 40 30 20 10 0 ppm

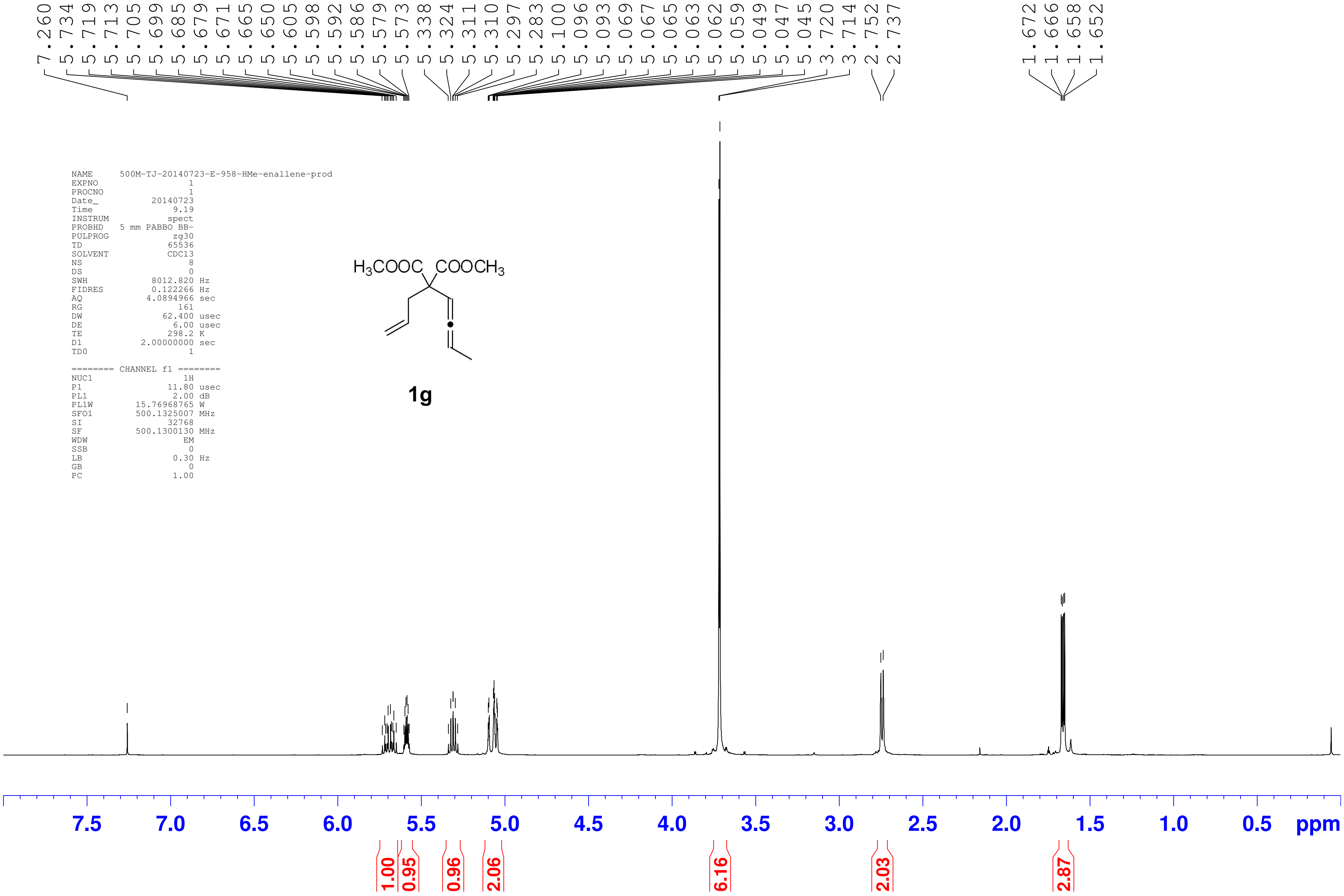


```
NAME       500M-TJ-20140723-E-958-HMe-enallene-prod
EXPNO                 1
PROCNO                1
Date_          20140723
Time               9.19
INSTRUM           spect
PROBHD   5 mm PABBO BB-
PULPROG            zg30
TD                65536
SOLVENT           CDCl3
NS                    8
DS                    0
SWH            8012.820 Hz
FIDRES         0.122266 Hz
AQ            4.0894966 sec
RG                  161
DW               62.400 usec
DE                 6.00 usec
TE                298.2 K
D1           2.00000000 sec
TD0                   1

======== CHANNEL f1 ========
NUC1                 1H
P1                11.80 usec
PL1                2.00 dB
PL1W        15.76968765 W
SFO1        500.1325007 MHz
SI                32768
SF          500.1300130 MHz
WDW                  EM
SSB                   0
LB                 0.30 Hz
GB                    0
PC                 1.00
```

1g

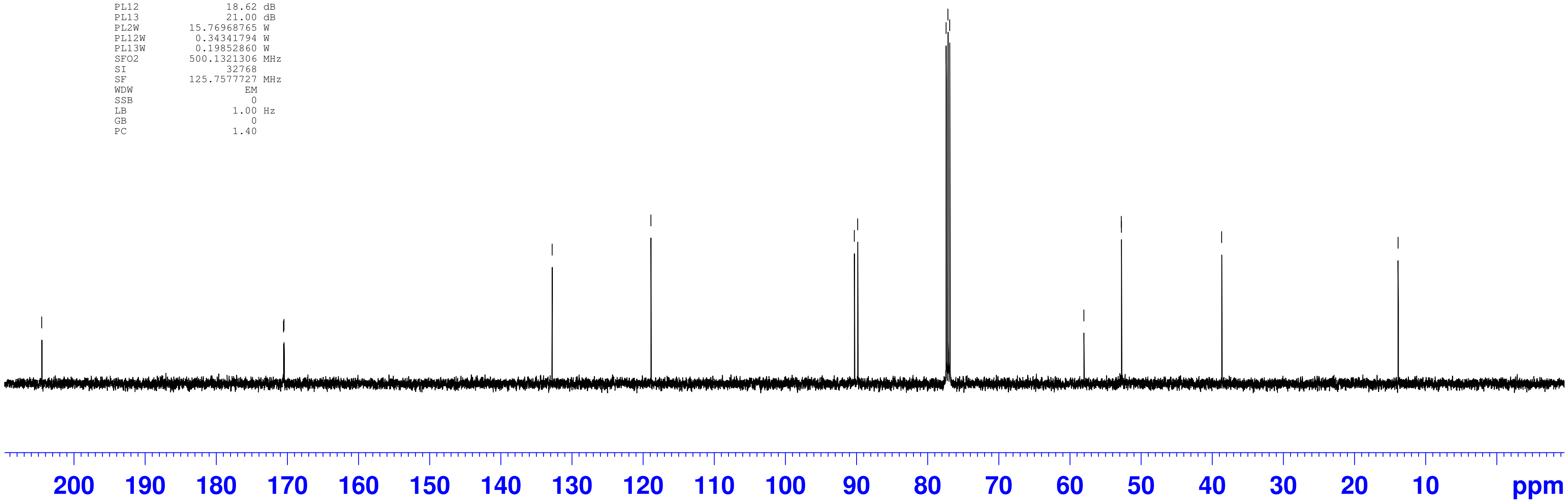

NAME	500M-TJ-20140723-E-958-HMe-enallene-prod
EXPNO	2
PROCNO	1
Date_	20140723
Time	9.22
INSTRUM	spect
PROBHD	5 mm PABBO BB-
PULPROG	zgpg30
TD	32768
SOLVENT	CDCl3
NS	32
DS	0
SWH	27573.529 Hz
FIDRES	0.841477 Hz
AQ	0.5942430 sec
RG	2050
DW	18.133 usec
DE	12.00 usec
TE	298.3 K
D1	2.00000000 sec
D11	0.03000000 sec
TD0	1

======== CHANNEL f1 ========

NUC1	13C
P1	7.50 usec
PL1	0.00 dB
PL1W	83.89700317 W
SFO1	125.7703648 MHz

======== CHANNEL f2 ========

CPDPRG2	waltz16
NUC2	1H
PCPD2	80.00 usec
PL2	2.00 dB
PL12	18.62 dB
PL13	21.00 dB
PL2W	15.76968765 W
PL12W	0.34341794 W
PL13W	0.19852860 W
SFO2	500.1321306 MHz
SI	32768
SF	125.7577727 MHz
WDW	EM
SSB	0
LB	1.00 Hz
GB	0
PC	1.40

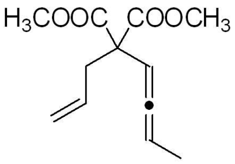

1g

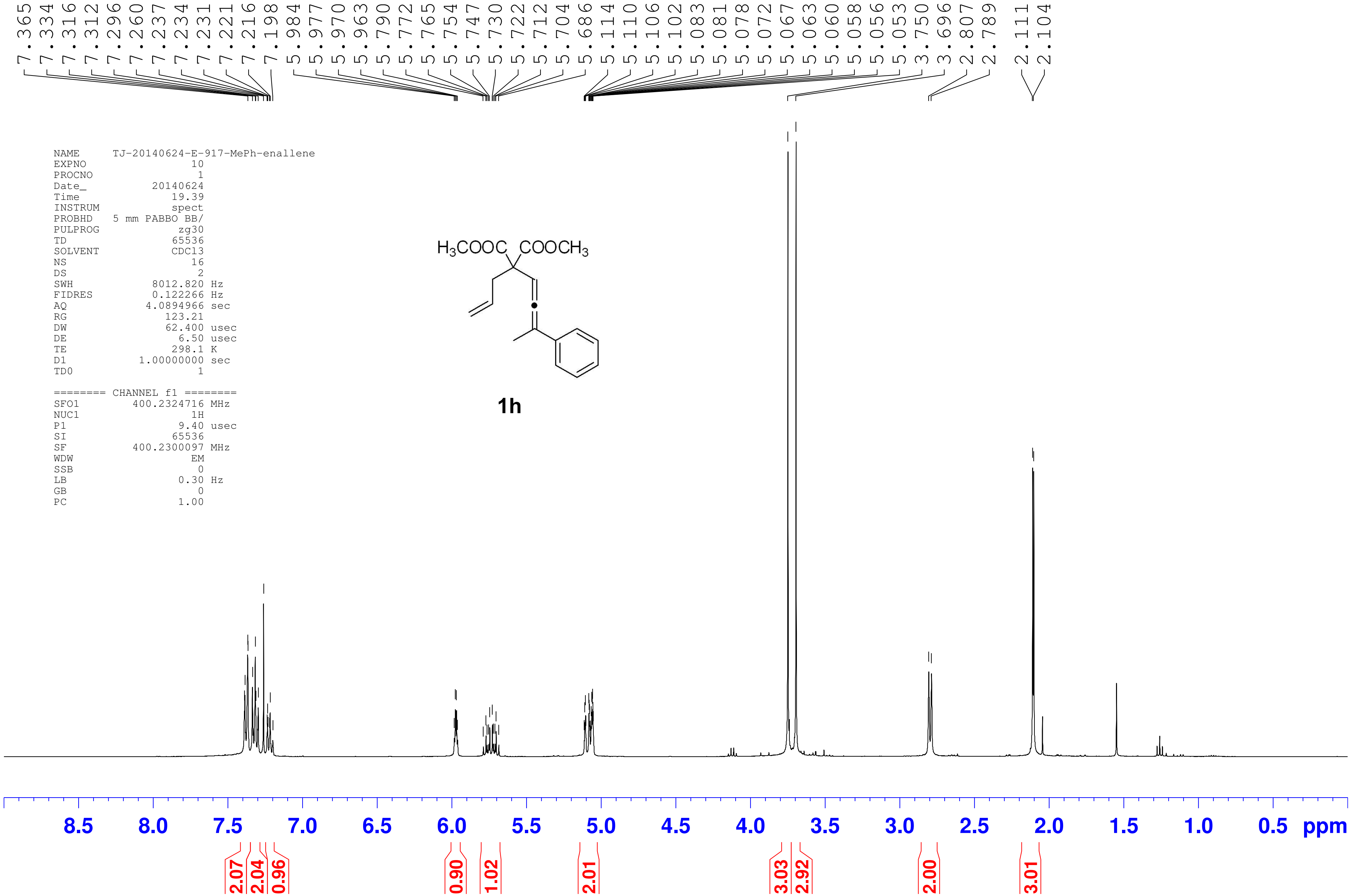
7.365
7.334
7.316
7.312
7.296
7.260
7.237
7.234
7.231
7.221
7.216
7.198
5.984
5.977
5.970
5.963
5.790
5.772
5.765
5.754
5.747
5.730
5.722
5.712
5.704
5.686
5.114
5.110
5.106
5.102
5.083
5.081
5.078
5.072
5.067
5.063
5.060
5.058
5.056
5.053
3.750
3.696
2.807
2.789
2.111
2.104
NAME TJ-20140624-E-917-MePh-enallene
EXPNO 10
PROCNO 1
Date_ 20140624
Time 19.39
INSTRUM spect
PROBHD 5 mm PABBO BB/
PULPROG zg30
TD 65536
SOLVENT CDCl3
NS 16
DS 2
SWH 8012.820 Hz
FIDRES 0.122266 Hz
AQ 4.0894966 sec
RG 123.21
DW 62.400 usec
DE 6.50 usec
TE 298.1 K
D1 1.00000000 sec
TD0 1
======== CHANNEL f1 ========
SFO1 400.2324716 MHz
NUC1 1H
P1 9.40 usec
SI 65536
SF 400.2300097 MHz
WDW EM
SSB 0
LB 0.30 Hz
GB 0
PC 1.00
H3COOC COOCH3
1h
8.5
8.0
7.5
7.0
6.5
6.0
5.5
5.0
4.5
4.0
3.5
3.0
2.5
2.0
1.5
1.0
0.5
ppm
2.07
2.04
0.96
0.90
1.02
2.01
3.03
2.92
2.00
3.01

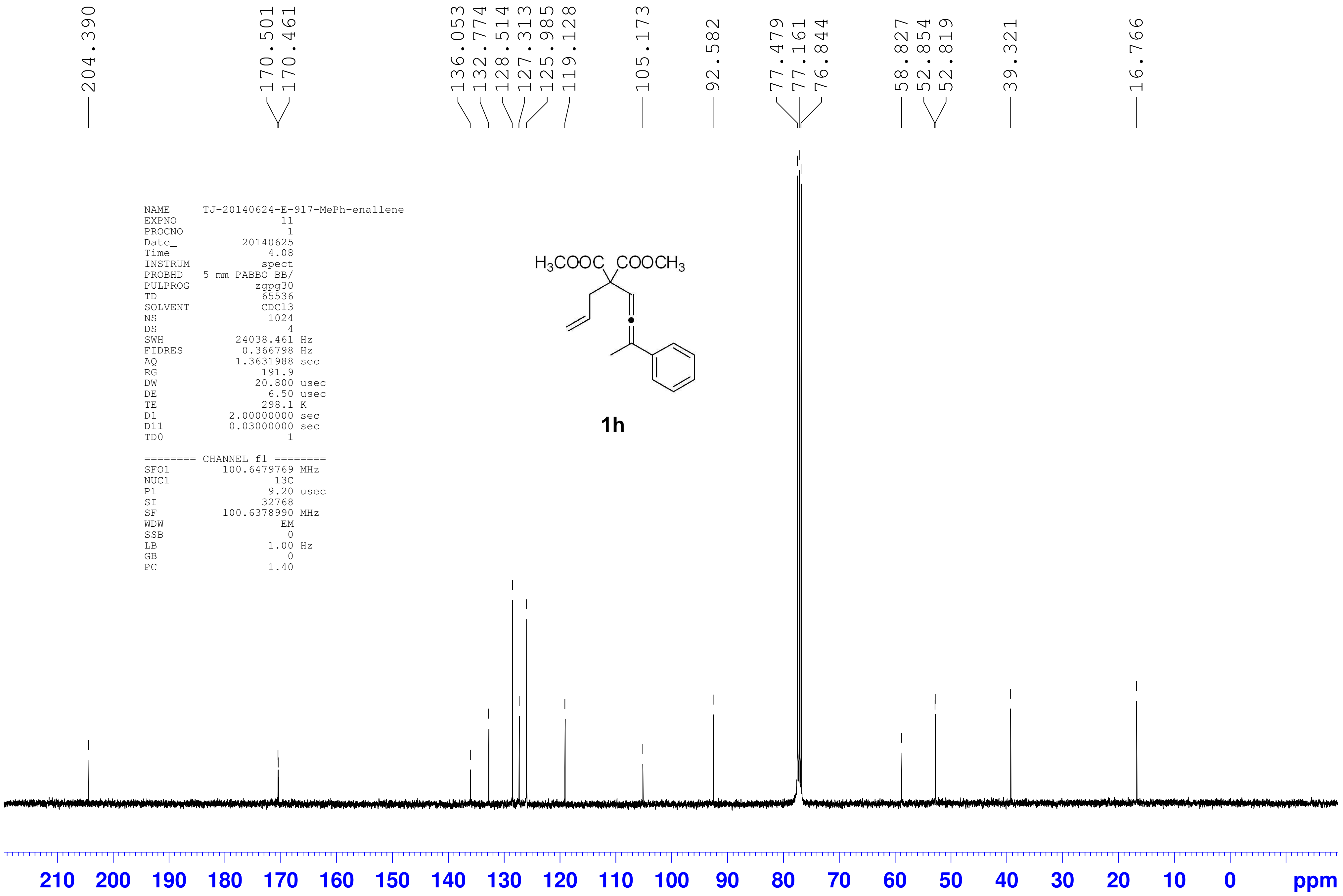

1h

```
NAME      TJ-20140624-E-917-MePh-enallene
EXPNO                    11
PROCNO                    1
Date_              20140625
Time                   4.08
INSTRUM               spect
PROBHD    5 mm PABBO BB/
PULPROG             zgpg30
TD                    65536
SOLVENT               CDCl3
NS                     1024
DS                        4
SWH               24038.461 Hz
FIDRES             0.366798 Hz
AQ                1.3631988 sec
RG                    191.9
DW                   20.800 usec
DE                     6.50 usec
TE                    298.1 K
D1               2.00000000 sec
D11              0.03000000 sec
TD0                       1

======== CHANNEL f1 ========
SFO1            100.6479769 MHz
NUC1                    13C
P1                     9.20 usec
SI                    32768
SF              100.6378990 MHz
WDW                      EM
SSB                       0
LB                     1.00 Hz
GB                        0
PC                     1.40
```

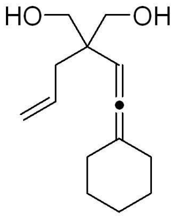
HO
OH

S1

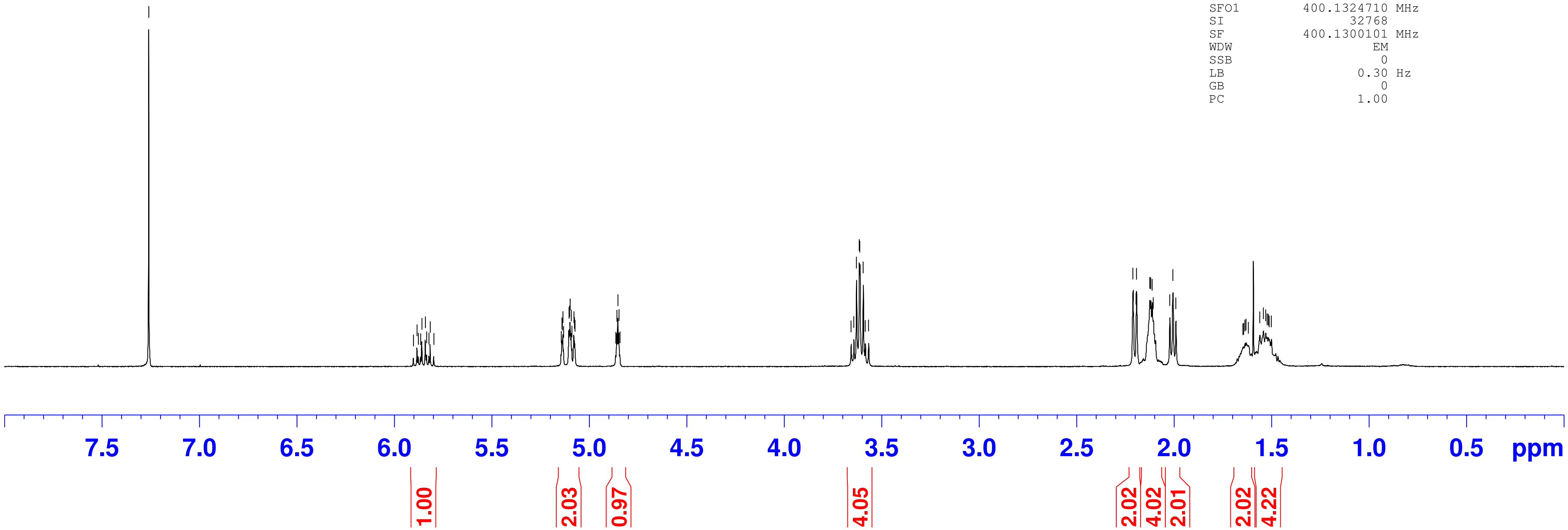
7.260
5.884
5.877
5.865
5.859
5.841
5.835
5.823
5.816
5.798
5.144
5.141
5.139
5.136
5.132
5.104
5.102
5.098
5.093
5.090
5.079
5.076
5.074
4.864
4.859
4.853
4.848
4.843
3.658
3.644
3.630
3.616
3.613
3.596
3.585
3.568
2.212
2.209
2.196
2.193
2.125
2.120
2.113
2.107
2.023
2.007
1.992
1.647
1.644
1.637
1.631
1.619
1.561
1.542
1.530
1.523
1.517
1.513
1.502
NAME TJ-20141106-E-1022-cy-LAH-frac
EXPNO 10
PROCNO 1
Date_ 20141106
Time 10.27
INSTRUM spect
PROBHD 5 mm PABBO BB-
PULPROG zg30
TD 65536
SOLVENT CDCl3
NS 16
DS 2
SWH 8223.685 Hz
FIDRES 0.125483 Hz
AQ 3.9846387 sec
RG 912
DW 60.800 usec
DE 6.50 usec
TE 673.2 K
D1 1.00000000 sec
TD0 1
======== CHANNEL f1 ========
NUC1 1H
P1 8.90 usec
PL1 -4.00 dB
PL1W 24.73352814 W
SFO1 400.1324710 MHz
SI 32768
SF 400.1300101 MHz
WDW EM
SSB 0
LB 0.30 Hz
GB 0
PC 1.00
7.5
7.0
6.5
6.0
5.5
5.0
4.5
4.0
3.5
3.0
2.5
2.0
1.5
1.0
0.5
ppm
1.00
2.03
0.97
4.05
2.02
4.02
2.01
2.02
4.22

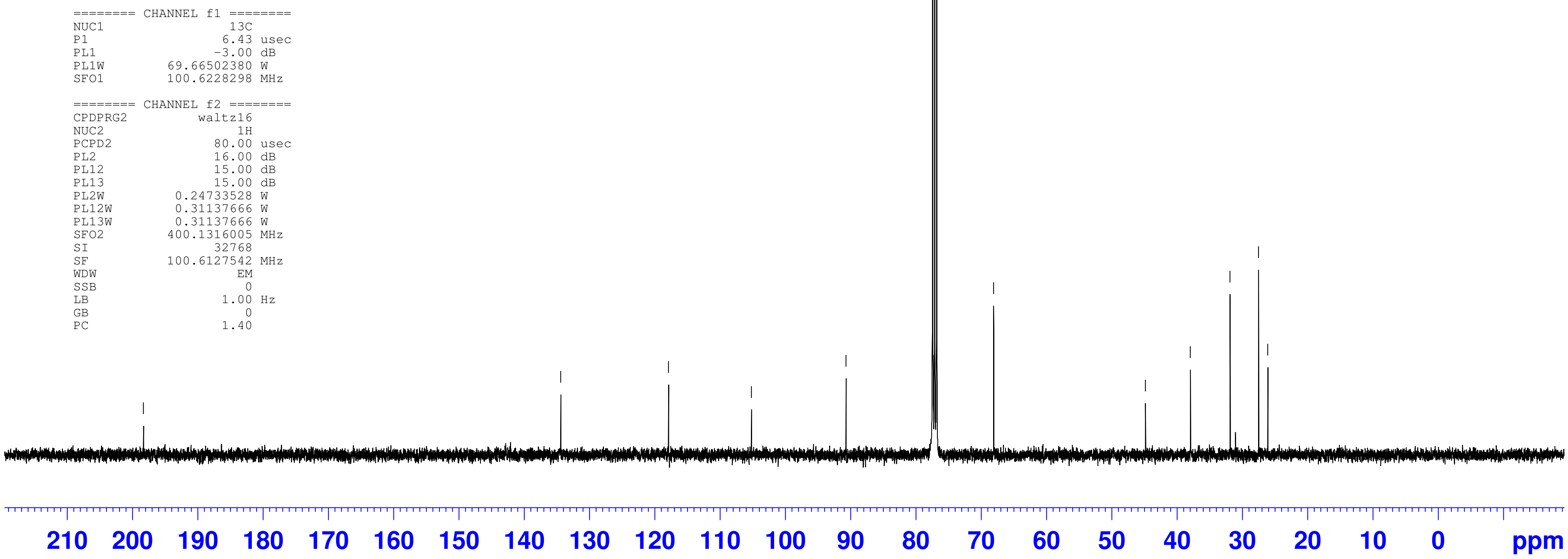


```
NAME      TJ-20141106-E-1022-cy-LAH-frac
EXPNO                   20
PROCNO                   1
Date_             20141110
Time                 23.03
INSTRUM              spect
PROBHD    5 mm PABBO BB-
PULPROG            zgpg30
TD                  65536
SOLVENT             CDCl3
NS                   3072
DS                      4
SWH             24038.461 Hz
FIDRES           0.366798 Hz
AQ              1.3631988 sec
RG                   2050
DW                 20.800 usec
DE                  51.93 usec
TE                  298.2 K
D1             2.00000000 sec
D11            0.03000000 sec
TD0                     1

======== CHANNEL f1 ========
NUC1                  13C
P1                   6.43 usec
PL1                 -3.00 dB
PL1W          69.66502380 W
SFO1          100.6228298 MHz

======== CHANNEL f2 ========
CPDPRG2           waltz16
NUC2                   1H
PCPD2               80.00 usec
PL2                 16.00 dB
PL12                15.00 dB
PL13                15.00 dB
PL2W           0.24733528 W
PL12W          0.31137666 W
PL13W          0.31137666 W
SFO2          400.1316005 MHz
SI                  32768
SF            100.6127542 MHz
WDW                    EM
SSB                     0
LB                   1.00 Hz
GB                      0
PC                   1.40
```

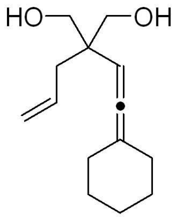

S1

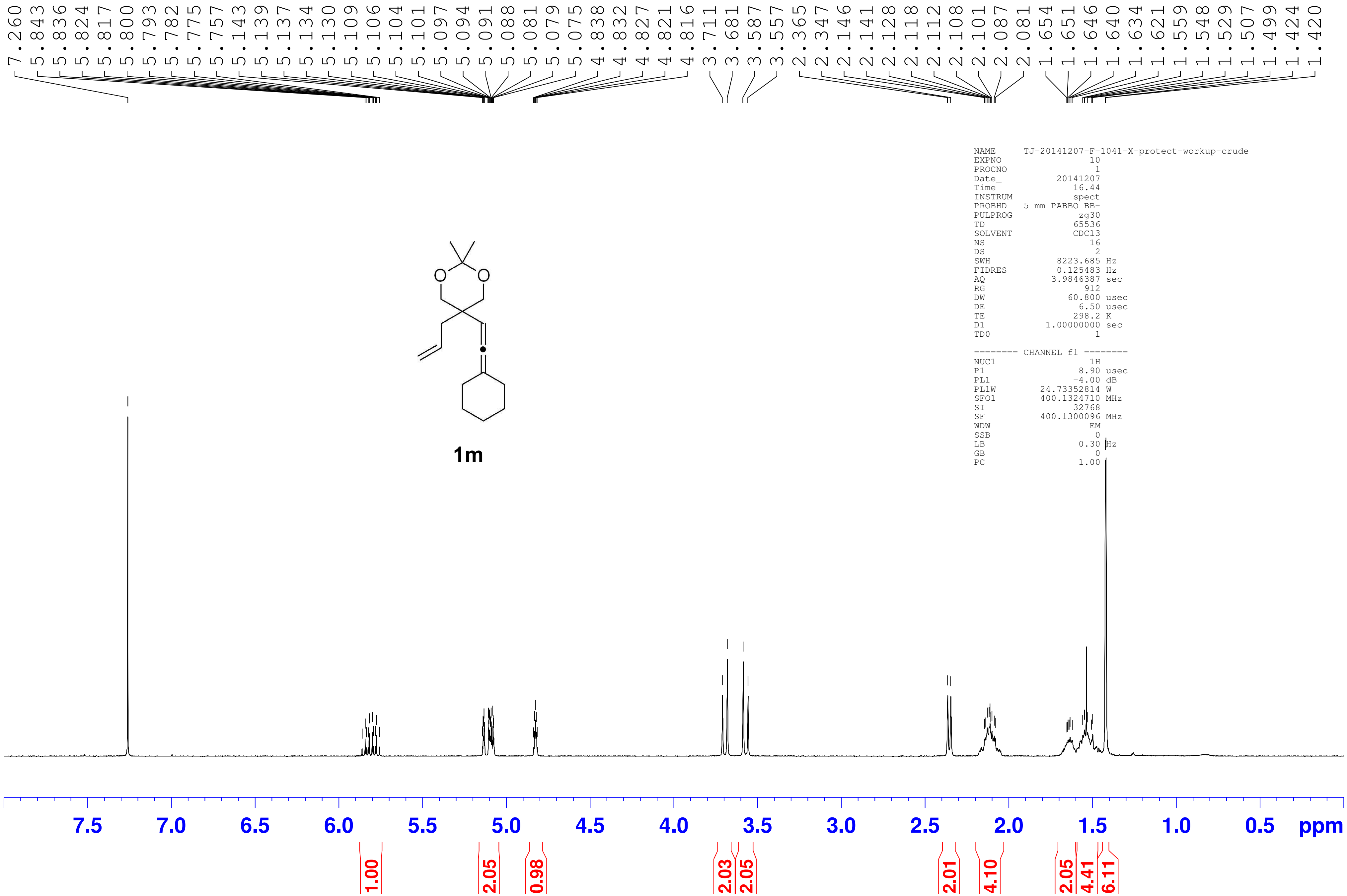
7.260
5.843
5.836
5.824
5.817
5.800
5.793
5.782
5.775
5.757
5.143
5.139
5.137
5.134
5.130
5.109
5.106
5.104
5.101
5.097
5.094
5.091
5.088
5.081
5.079
5.075
4.838
4.832
4.827
4.821
4.816
3.711
3.681
3.587
3.557
2.365
2.347
2.146
2.141
2.128
2.118
2.112
2.108
2.101
2.087
2.081
1.654
1.651
1.646
1.640
1.634
1.621
1.559
1.548
1.529
1.507
1.499
1.424
1.420
NAME TJ-20141207-F-1041-X-protect-workup-crude
EXPNO 10
PROCNO 1
Date_ 20141207
Time 16.44
INSTRUM spect
PROBHD 5 mm PABBO BB-
PULPROG zg30
TD 65536
SOLVENT CDCl3
NS 16
DS 2
SWH 8223.685 Hz
FIDRES 0.125483 Hz
AQ 3.9846387 sec
RG 912
DW 60.800 usec
DE 6.50 usec
TE 298.2 K
D1 1.00000000 sec
TD0 1
======== CHANNEL f1 ========
NUC1 1H
P1 8.90 usec
PL1 -4.00 dB
PL1W 24.73352814 W
SFO1 400.1324710 MHz
SI 32768
SF 400.1300096 MHz
WDW EM
SSB 0
LB 0.30 Hz
GB 0
PC 1.00
7.5
7.0
6.5
6.0
5.5
5.0
4.5
4.0
3.5
3.0
2.5
2.0
1.5
1.0
0.5
ppm
1.00
2.05
0.98
2.03
2.05
2.01
4.10
2.05
4.41
6.11

1m

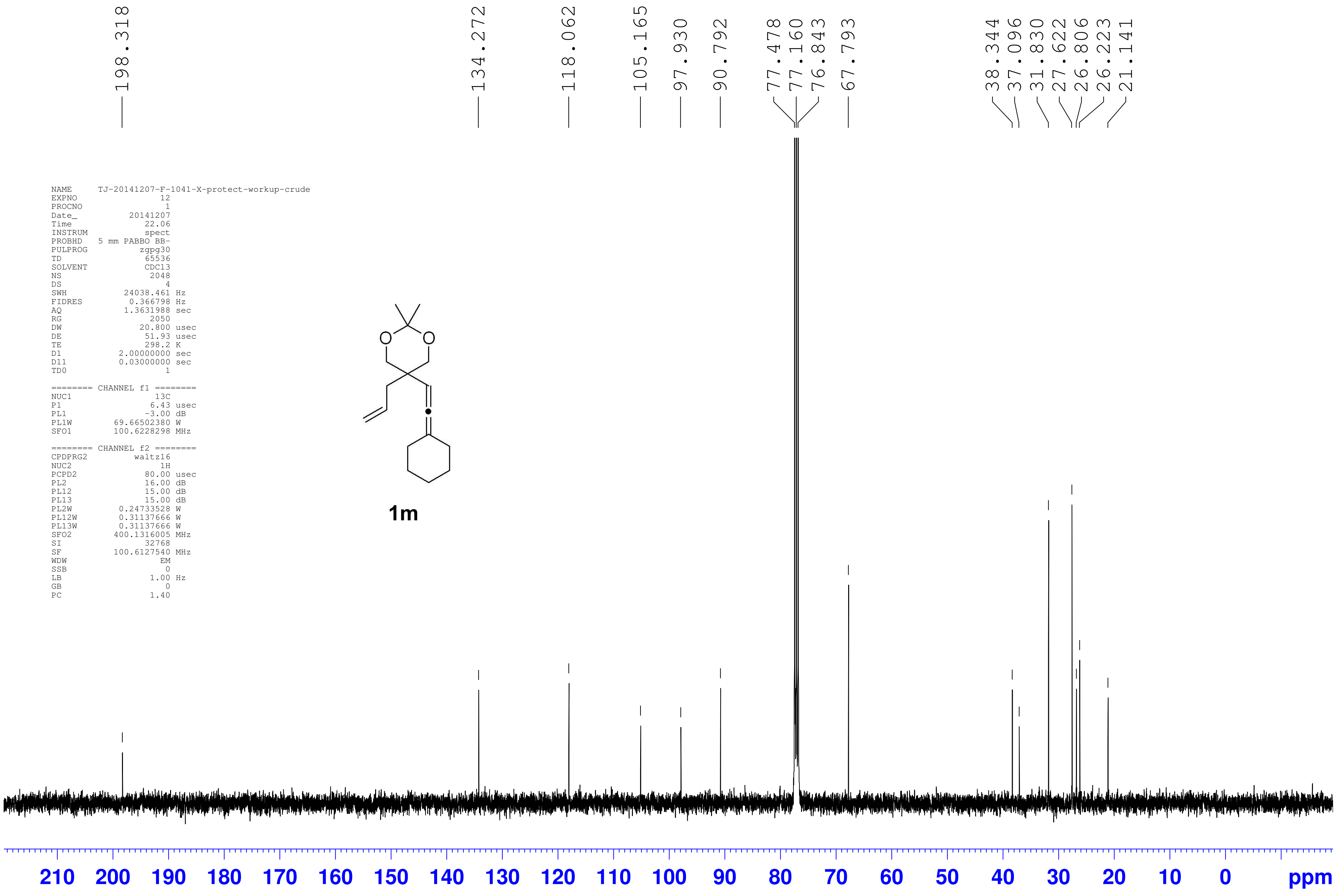
198.318
134.272
118.062
105.165
97.930
90.792
77.478
77.160
76.843
67.793
38.344
37.096
31.830
27.622
26.806
26.223
21.141
NAME TJ-20141207-F-1041-X-protect-workup-crude
EXPNO 12
PROCNO 1
Date_ 20141207
Time 22.06
INSTRUM spect
PROBHD 5 mm PABBO BB-
PULPROG zgpg30
TD 65536
SOLVENT CDCl3
NS 2048
DS 4
SWH 24038.461 Hz
FIDRES 0.366798 Hz
AQ 1.3631988 sec
RG 2050
DW 20.800 usec
DE 51.93 usec
TE 298.2 K
D1 2.00000000 sec
D11 0.03000000 sec
TD0 1
======== CHANNEL f1 ========
NUC1 13C
P1 6.43 usec
PL1 -3.00 dB
PL1W 69.66502380 W
SFO1 100.6228298 MHz
======== CHANNEL f2 ========
CPDPRG2 waltz16
NUC2 1H
PCPD2 80.00 usec
PL2 16.00 dB
PL12 15.00 dB
PL13 15.00 dB
PL2W 0.24733528 W
PL12W 0.31137666 W
PL13W 0.31137666 W
SFO2 400.1316005 MHz
SI 32768
SF 100.6127540 MHz
WDW EM
SSB 0
LB 1.00 Hz
GB 0
PC 1.40
O
O
1m
210 200 190 180 170 160 150 140 130 120 110 100 90 80 70 60 50 40 30 20 10 0 ppm

7.260
7.202

2.249
1.828
1.461

NAME TJ-20140826-(S)-funki-Pacid-re
EXPNO 10
PROCNO 1
Date_ 20140826
Time 12.25
INSTRUM spect
PROBHD 5 mm PABBO BB/
PULPROG zg30
TD 65536
SOLVENT CDCl3
NS 16
DS 2
SWH 8012.820 Hz
FIDRES 0.122266 Hz
AQ 4.0894966 sec
RG 96.29
DW 62.400 usec
DE 6.50 usec
TE 298.2 K
D1 1.00000000 sec
TD0 1

======== CHANNEL f1 ========
SFO1 400.2424716 MHz
NUC1 1H
P1 9.40 usec
SI 65536
SF 400.2400096 MHz
WDW EM
SSB 0
LB 0.30 Hz
GB 0
PC 1.00

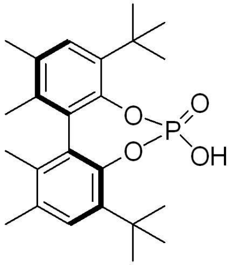

4a

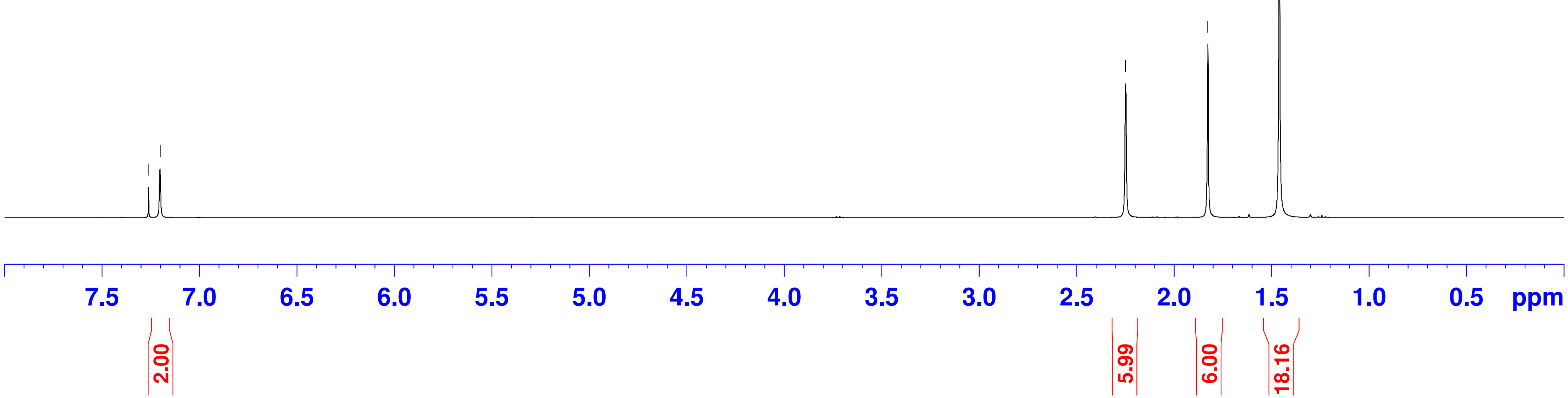

NAME TJ-20140826-(S)-funki-Pacid-re
EXPNO 13
PROCNO 1
Date_ 20140826
Time 12.53
INSTRUM spect
PROBHD 5 mm PABBO BB/
PULPROG zgpg30
TD 65536
SOLVENT CDCl3
NS 176
DS 4
SWH 24038.461 Hz
FIDRES 0.366798 Hz
AQ 1.3631988 sec
RG 191.9
DW 20.800 usec
DE 6.50 usec
TE 298.1 K
D1 2.00000000 sec
D11 0.03000000 sec
TD0 1

======== CHANNEL f1 ========
SFO1 100.6504912 MHz
NUC1 13C
P1 9.20 usec
SI 32768
SF 100.6404139 MHz
WDW EM
SSB 0
LB 1.00 Hz
GB 0
PC 1.40

4a

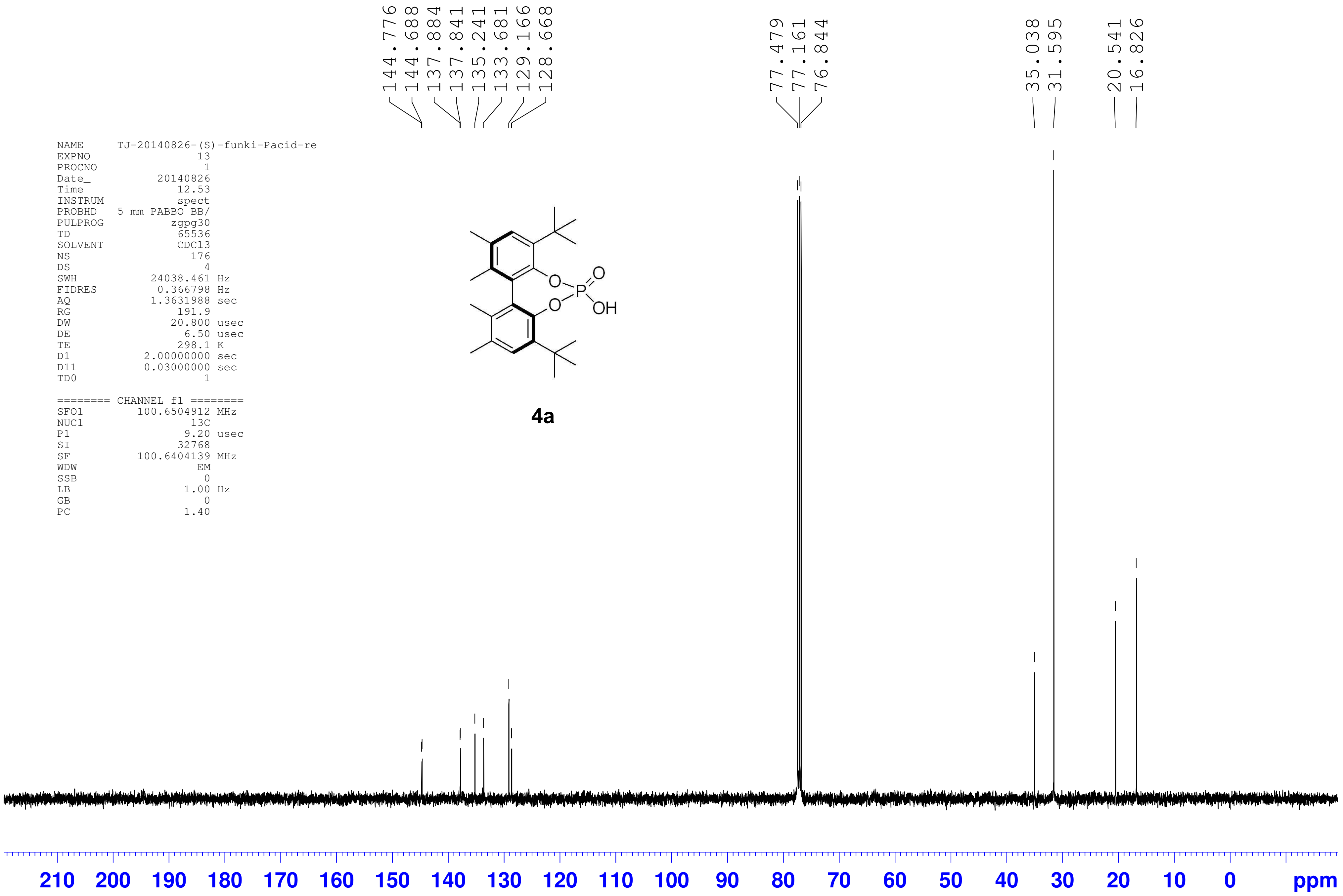

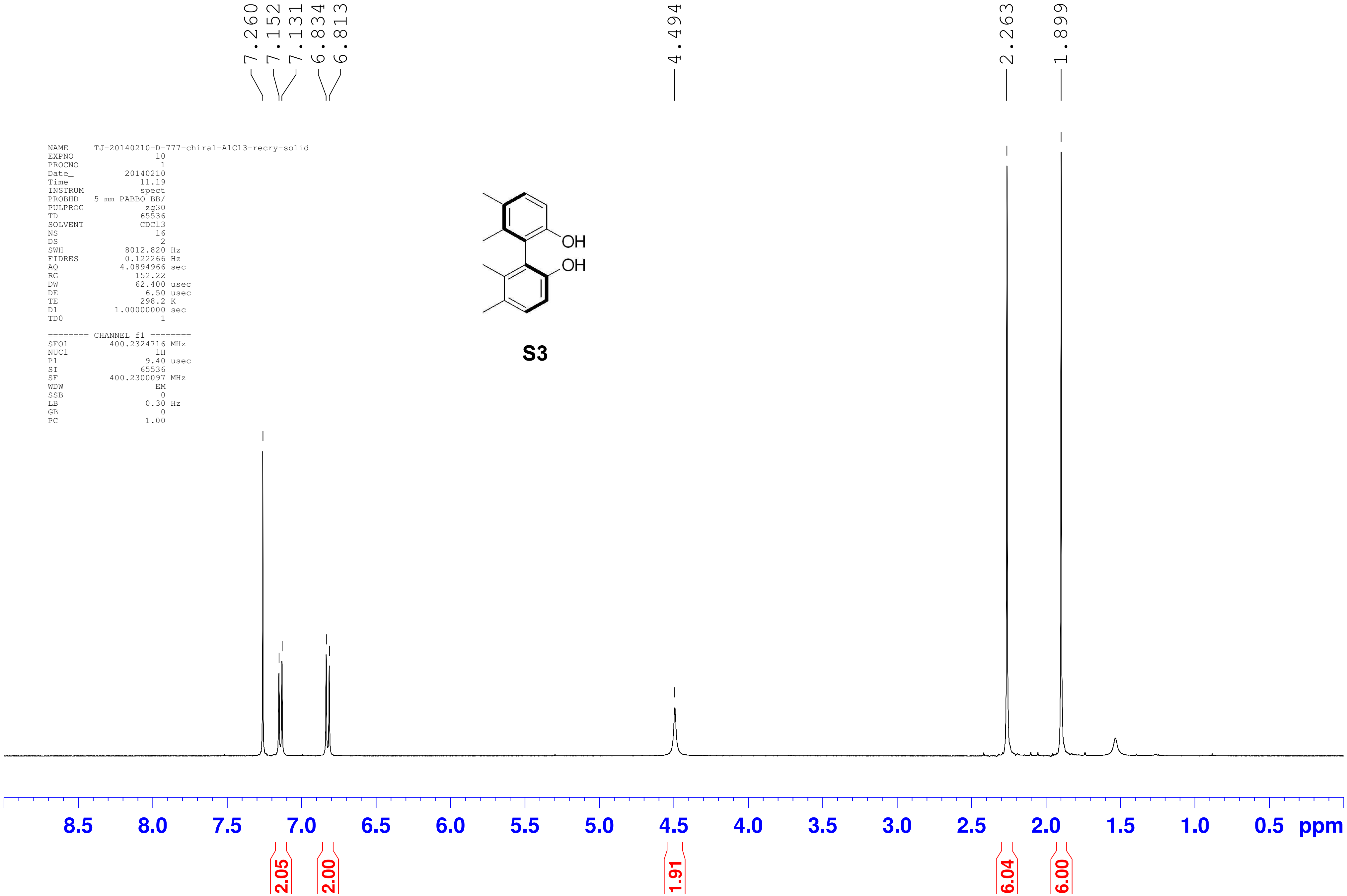
7.260
7.152
7.131
6.834
6.813
4.494
2.263
1.899
NAME TJ-20140210-D-777-chiral-AlCl3-recry-solid
EXPNO 10
PROCNO 1
Date_ 20140210
Time 11.19
INSTRUM spect
PROBHD 5 mm PABBO BB/
PULPROG zg30
TD 65536
SOLVENT CDCl3
NS 16
DS 2
SWH 8012.820 Hz
FIDRES 0.122266 Hz
AQ 4.0894966 sec
RG 152.22
DW 62.400 usec
DE 6.50 usec
TE 298.2 K
D1 1.00000000 sec
TD0 1
======== CHANNEL f1 ========
SFO1 400.2324716 MHz
NUC1 1H
P1 9.40 usec
SI 65536
SF 400.2300097 MHz
WDW EM
SSB 0
LB 0.30 Hz
GB 0
PC 1.00
OH
OH
S3
8.5
8.0
7.5
7.0
6.5
6.0
5.5
5.0
4.5
4.0
3.5
3.0
2.5
2.0
1.5
1.0
0.5
ppm
2.05
2.00
1.91
6.04
6.00

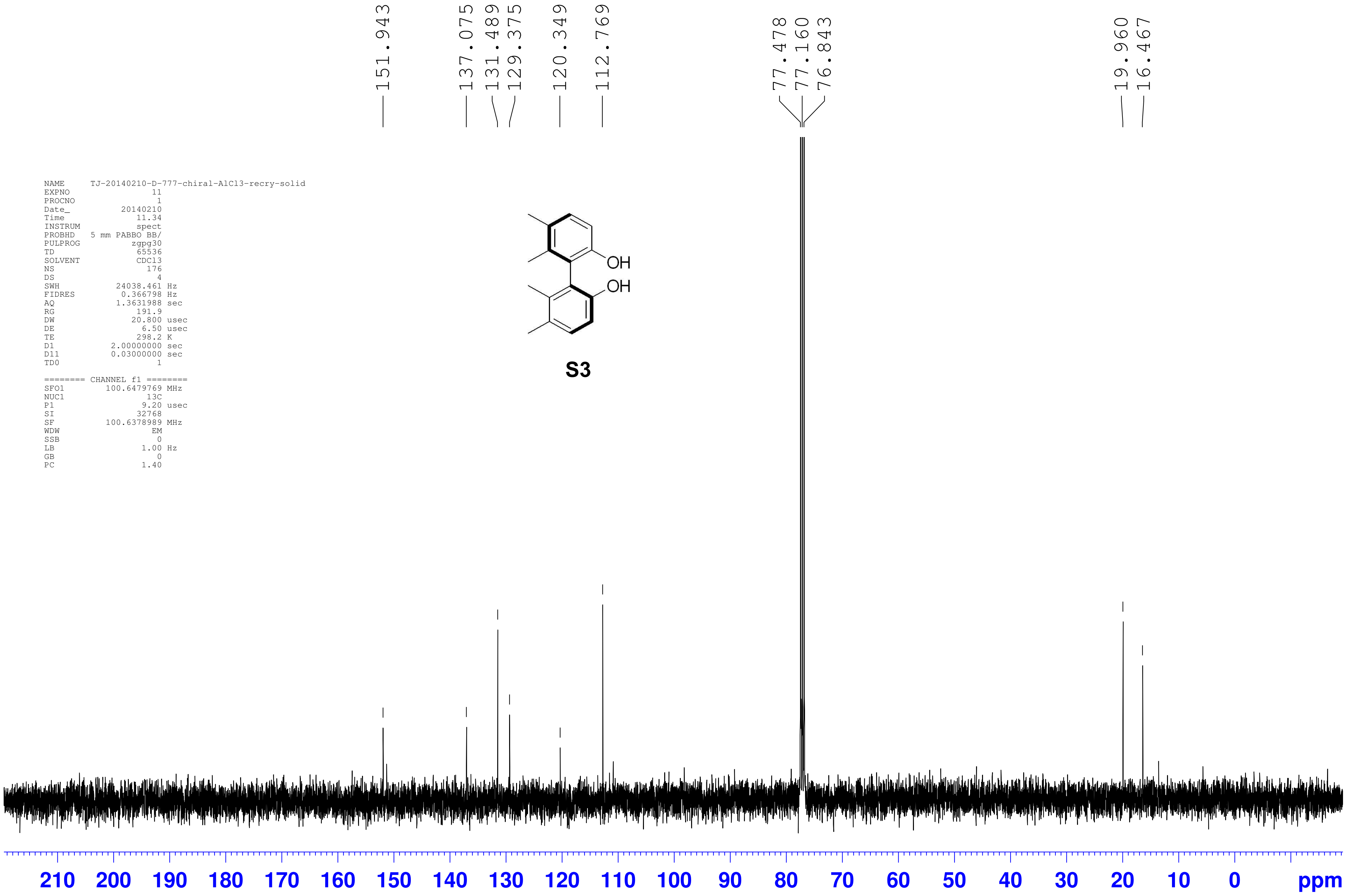

151.943
137.075
131.489
129.375
120.349
112.769
77.478
77.160
76.843
19.960
16.467
NAME TJ-20140210-D-777-chiral-AlCl3-recry-solid
EXPNO 11
PROCNO 1
Date_ 20140210
Time 11.34
INSTRUM spect
PROBHD 5 mm PABBO BB/
PULPROG zgpg30
TD 65536
SOLVENT CDCl3
NS 176
DS 4
SWH 24038.461 Hz
FIDRES 0.366798 Hz
AQ 1.3631988 sec
RG 191.9
DW 20.800 usec
DE 6.50 usec
TE 298.2 K
D1 2.00000000 sec
D11 0.03000000 sec
TD0 1
======== CHANNEL f1 ========
SFO1 100.6479769 MHz
NUC1 13C
P1 9.20 usec
SI 32768
SF 100.6378989 MHz
WDW EM
SSB 0
LB 1.00 Hz
GB 0
PC 1.40
OH
OH
S3
210 200 190 180 170 160 150 140 130 120 110 100 90 80 70 60 50 40 30 20 10 0 ppm

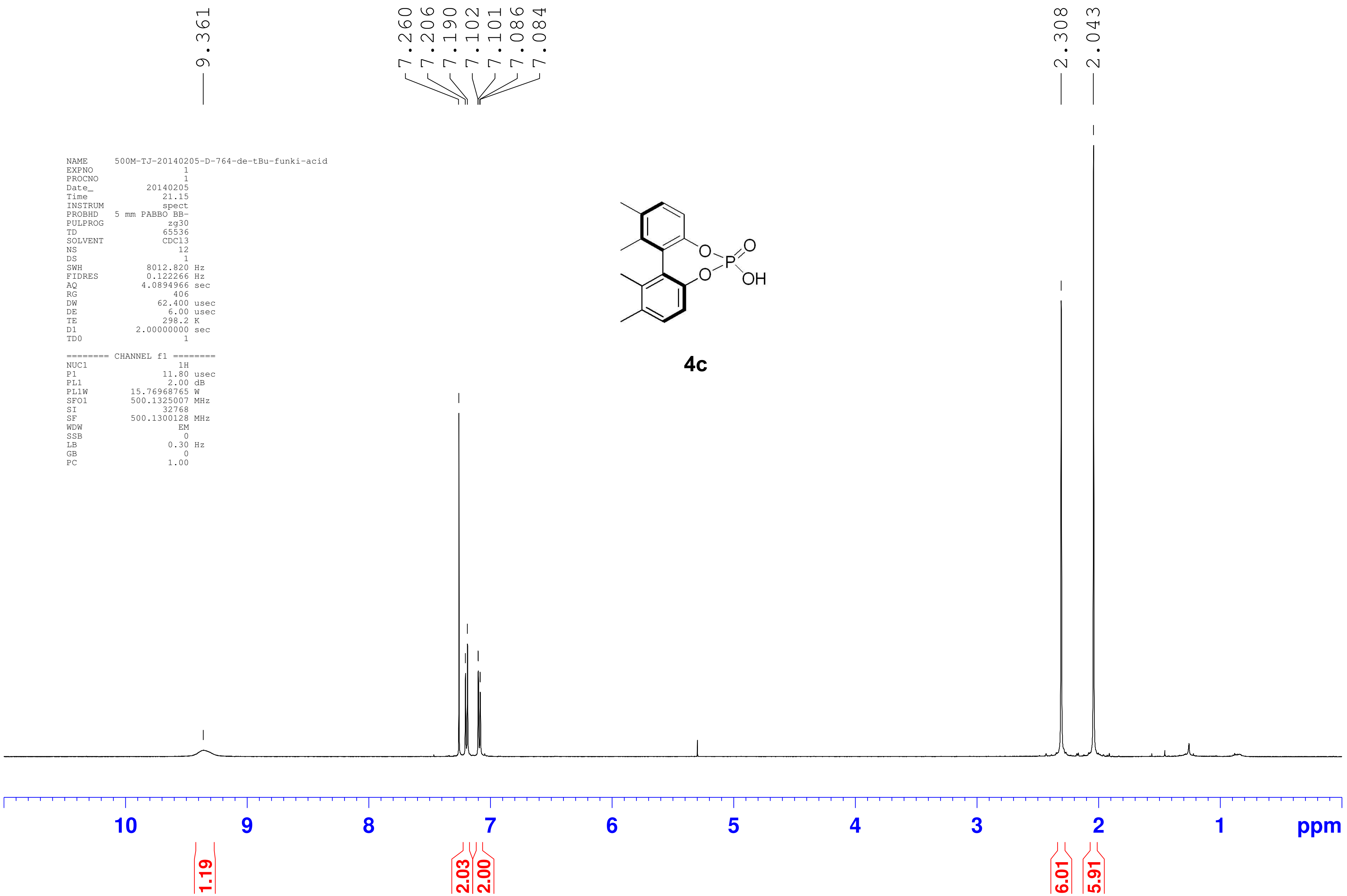
9.361
7.260
7.206
7.190
7.102
7.101
7.086
7.084
2.308
2.043
NAME 500M-TJ-20140205-D-764-de-tBu-funki-acid
EXPNO 1
PROCNO 1
Date_ 20140205
Time 21.15
INSTRUM spect
PROBHD 5 mm PABBO BB-
PULPROG zg30
TD 65536
SOLVENT CDCl3
NS 12
DS 1
SWH 8012.820 Hz
FIDRES 0.122266 Hz
AQ 4.0894966 sec
RG 406
DW 62.400 usec
DE 6.00 usec
TE 298.2 K
D1 2.00000000 sec
TD0 1
======== CHANNEL f1 ========
NUC1 1H
P1 11.80 usec
PL1 2.00 dB
PL1W 15.76968765 W
SFO1 500.1325007 MHz
SI 32768
SF 500.1300128 MHz
WDW EM
SSB 0
LB 0.30 Hz
GB 0
PC 1.00
O
P
O
O
OH
4c
10
9
8
7
6
5
4
3
2
1
ppm
1.19
2.03
2.00
6.01
5.91

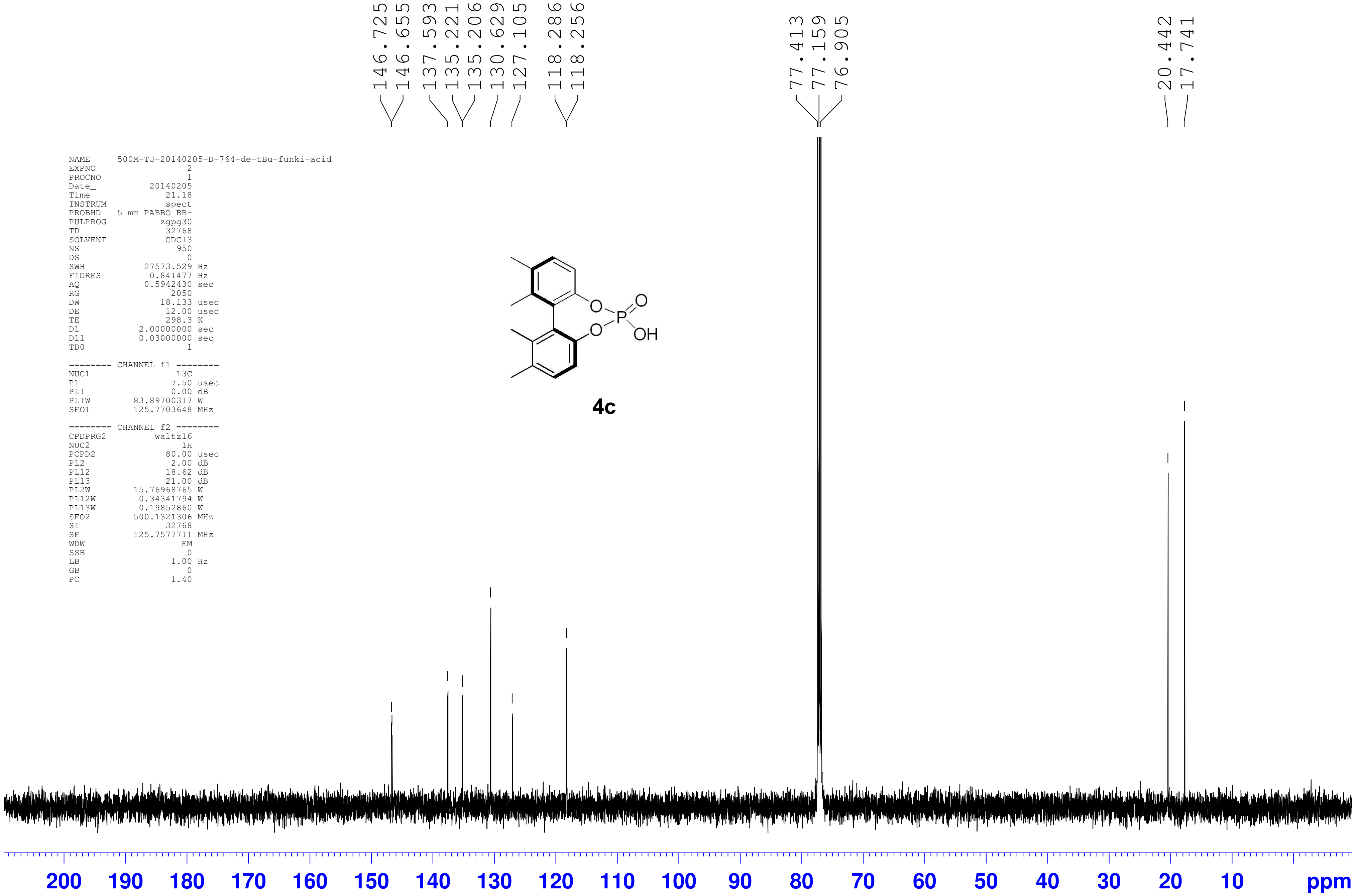


```
NAME       500M-TJ-20140205-D-764-de-tBu-funki-acid
EXPNO                    2
PROCNO                   1
Date_             20140205
Time                 21.18
INSTRUM              spect
PROBHD    5 mm PABBO BB-
PULPROG            zgpg30
TD                  32768
SOLVENT             CDCl3
NS                    950
DS                      0
SWH             27573.529 Hz
FIDRES           0.841477 Hz
AQ              0.5942430 sec
RG                   2050
DW                 18.133 usec
DE                  12.00 usec
TE                  298.3 K
D1             2.00000000 sec
D11            0.03000000 sec
TD0                     1

======== CHANNEL f1 ========
NUC1                  13C
P1                   7.50 usec
PL1                  0.00 dB
PL1W          83.89700317 W
SFO1          125.7703648 MHz

======== CHANNEL f2 ========
CPDPRG2           waltz16
NUC2                   1H
PCPD2               80.00 usec
PL2                  2.00 dB
PL12                18.62 dB
PL13                21.00 dB
PL2W          15.76968765 W
PL12W          0.34341794 W
PL13W          0.19852860 W
SFO2          500.1321306 MHz
SI                  32768
SF            125.7577711 MHz
WDW                    EM
SSB                     0
LB                   1.00 Hz
GB                      0
PC                   1.40
```

4c

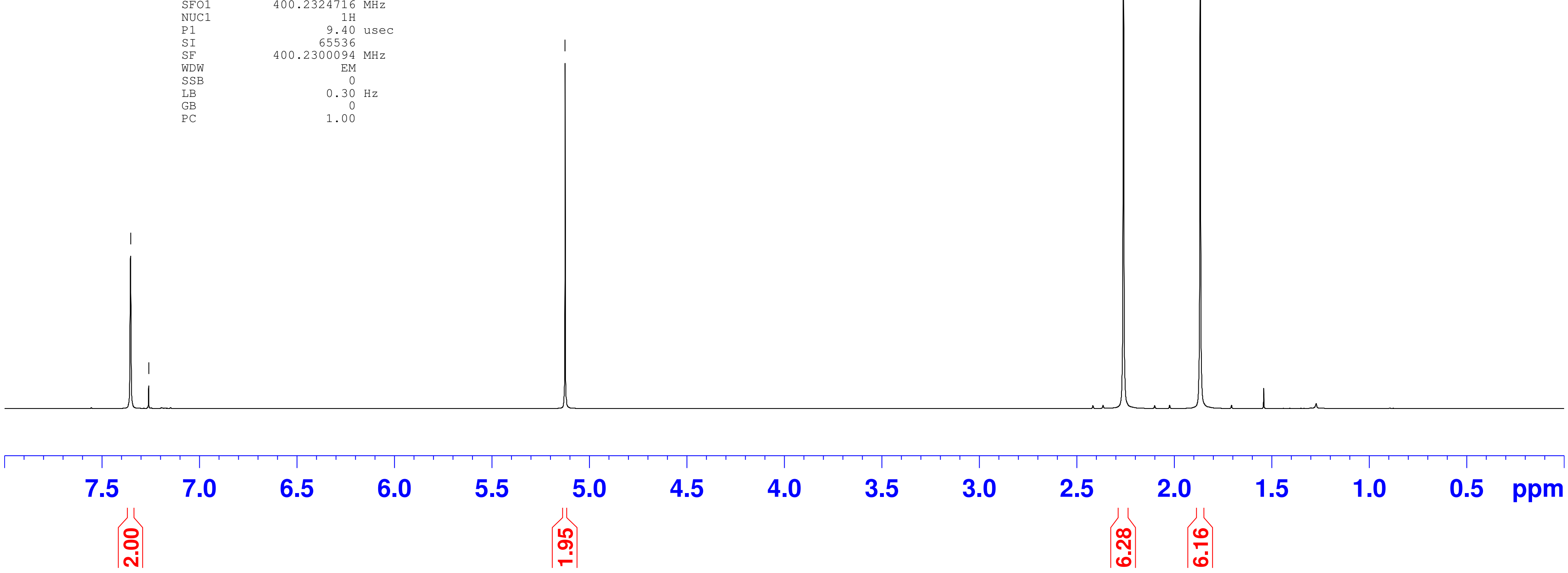


```
NAME      TJ-20140408-D-825-diOH-Br2-frac
EXPNO                  10
PROCNO                  1
Date_            20140408
Time                13.58
INSTRUM             spect
PROBHD    5 mm PABBO BB/
PULPROG              zg30
TD                  65536
SOLVENT             CDCl3
NS                     16
DS                      2
SWH              8012.820 Hz
FIDRES           0.122266 Hz
AQ              4.0894966 sec
RG                  54.55
DW                 62.400 usec
DE                   6.50 usec
TE                  298.2 K
D1             1.00000000 sec
TD0                     1

======== CHANNEL f1 ========
SFO1          400.2324716 MHz
NUC1                   1H
P1                   9.40 usec
SI                  65536
SF            400.2300094 MHz
WDW                    EM
SSB                     0
LB                   0.30 Hz
GB                      0
PC                   1.00
```

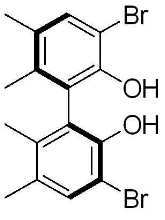

S4

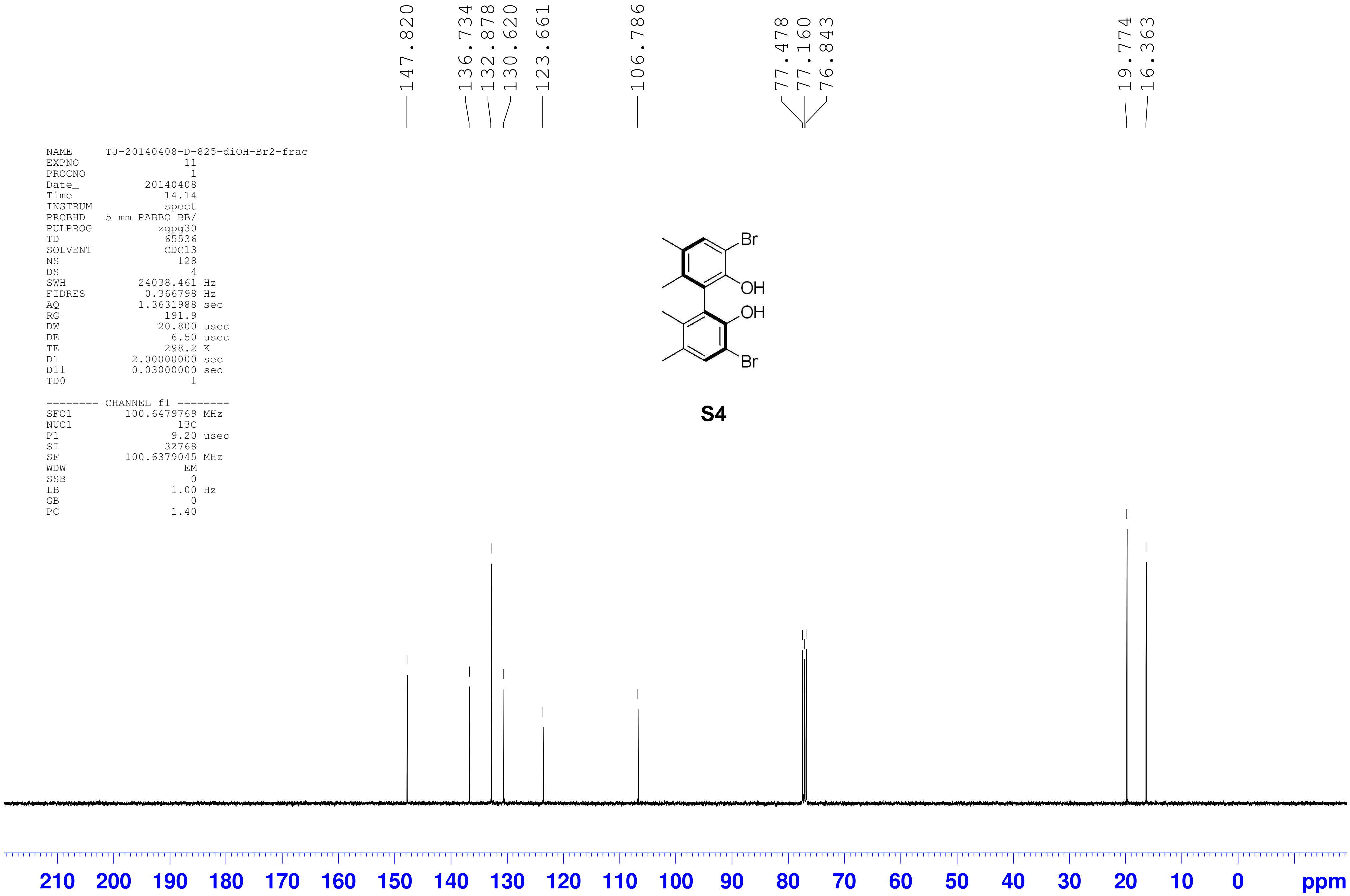

NAME TJ-20140408-D-825-diOH-Br2-frac
EXPNO 11
PROCNO 1
Date_ 20140408
Time 14.14
INSTRUM spect
PROBHD 5 mm PABBO BB/
PULPROG zgpg30
TD 65536
SOLVENT CDCl3
NS 128
DS 4
SWH 24038.461 Hz
FIDRES 0.366798 Hz
AQ 1.3631988 sec
RG 191.9
DW 20.800 usec
DE 6.50 usec
TE 298.2 K
D1 2.00000000 sec
D11 0.03000000 sec
TD0 1

======== CHANNEL f1 ========
SFO1 100.6479769 MHz
NUC1 13C
P1 9.20 usec
SI 32768
SF 100.6379045 MHz
WDW EM
SSB 0
LB 1.00 Hz
GB 0
PC 1.40

S4

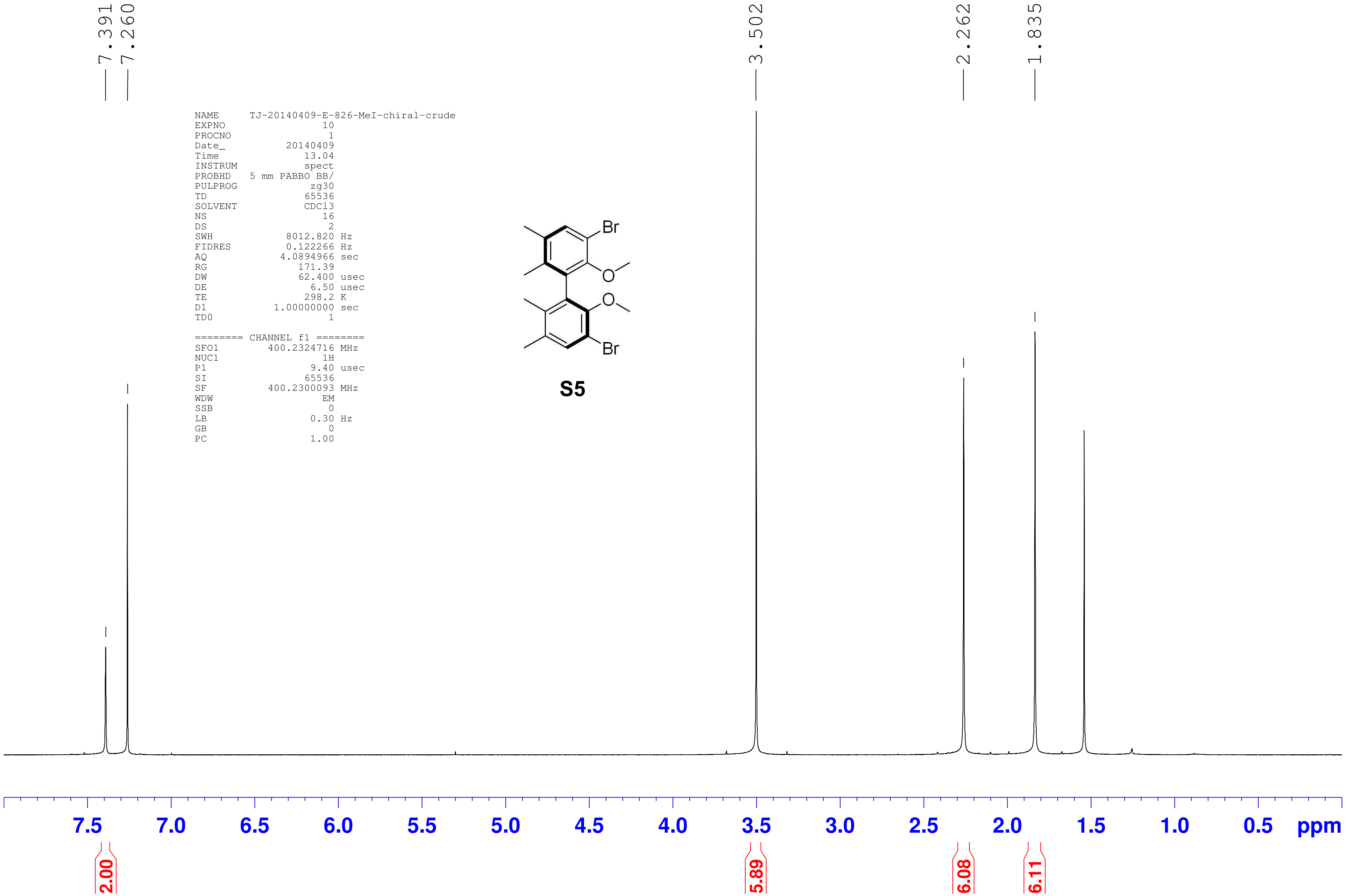
7.391
7.260
3.502
2.262
1.835
NAME TJ-20140409-E-826-MeI-chiral-crude
EXPNO 10
PROCNO 1
Date_ 20140409
Time 13.04
INSTRUM spect
PROBHD 5 mm PABBO BB/
PULPROG zg30
TD 65536
SOLVENT CDCl3
NS 16
DS 2
SWH 8012.820 Hz
FIDRES 0.122266 Hz
AQ 4.0894966 sec
RG 171.39
DW 62.400 usec
DE 6.50 usec
TE 298.2 K
D1 1.00000000 sec
TD0 1
======== CHANNEL f1 ========
SFO1 400.2324716 MHz
NUC1 1H
P1 9.40 usec
SI 65536
SF 400.2300093 MHz
WDW EM
SSB 0
LB 0.30 Hz
GB 0
PC 1.00
Br
O
O
Br
S5
7.5
7.0
6.5
6.0
5.5
5.0
4.5
4.0
3.5
3.0
2.5
2.0
1.5
1.0
0.5
ppm
2.00
5.89
6.08
6.11

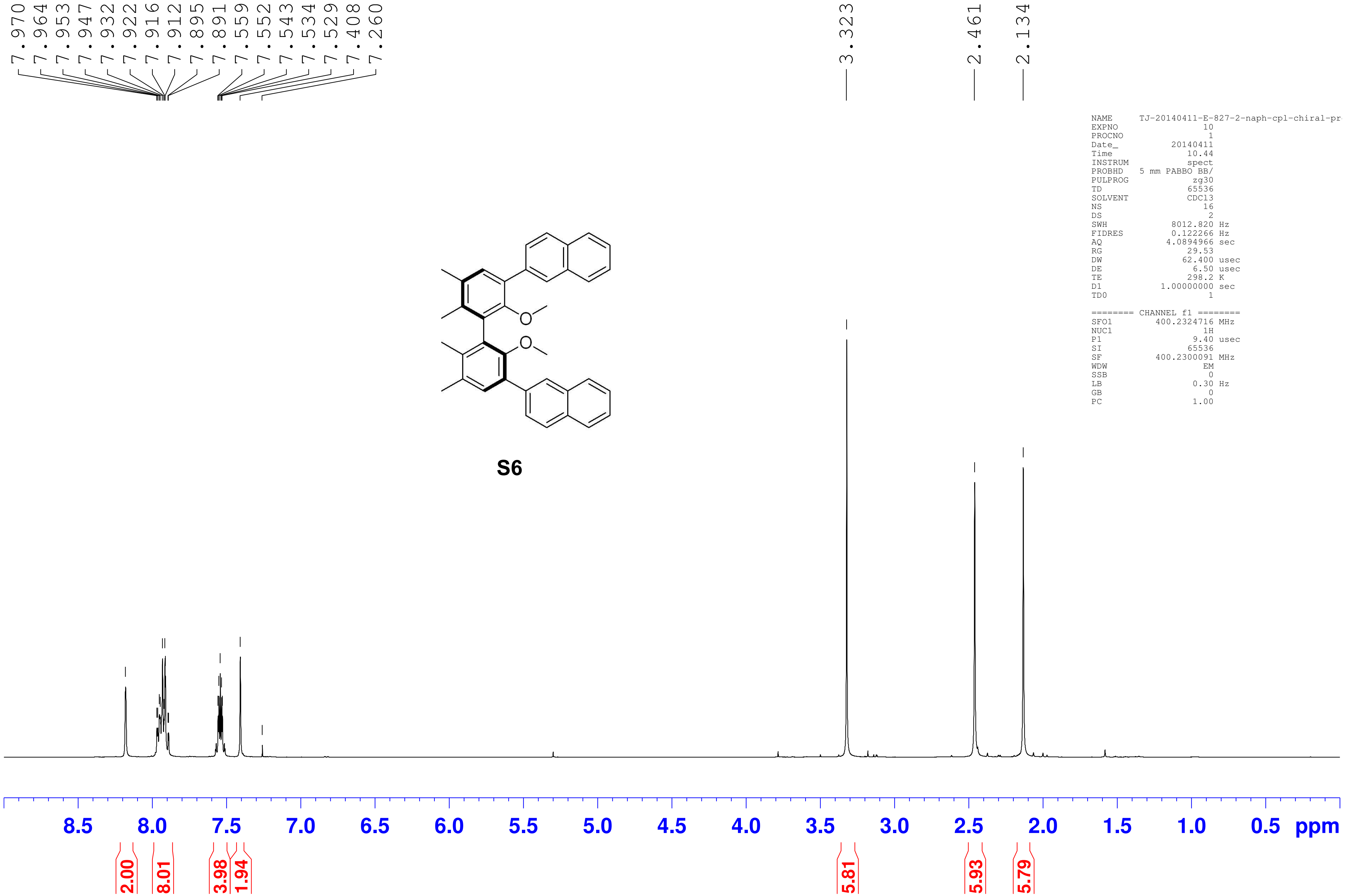
7.970
7.964
7.953
7.947
7.932
7.922
7.916
7.912
7.895
7.891
7.559
7.552
7.543
7.534
7.529
7.408
7.260
3.323
2.461
2.134
S6
NAME TJ-20140411-E-827-2-naph-cpl-chiral-pr
EXPNO 10
PROCNO 1
Date_ 20140411
Time 10.44
INSTRUM spect
PROBHD 5 mm PABBO BB/
PULPROG zg30
TD 65536
SOLVENT CDCl3
NS 16
DS 2
SWH 8012.820 Hz
FIDRES 0.122266 Hz
AQ 4.0894966 sec
RG 29.53
DW 62.400 usec
DE 6.50 usec
TE 298.2 K
D1 1.00000000 sec
TD0 1
======== CHANNEL f1 ========
SFO1 400.2324716 MHz
NUC1 1H
P1 9.40 usec
SI 65536
SF 400.2300091 MHz
WDW EM
SSB 0
LB 0.30 Hz
GB 0
PC 1.00
8.5
8.0
7.5
7.0
6.5
6.0
5.5
5.0
4.5
4.0
3.5
3.0
2.5
2.0
1.5
1.0
0.5
ppm
2.00
8.01
3.98
1.94
5.81
5.93
5.79

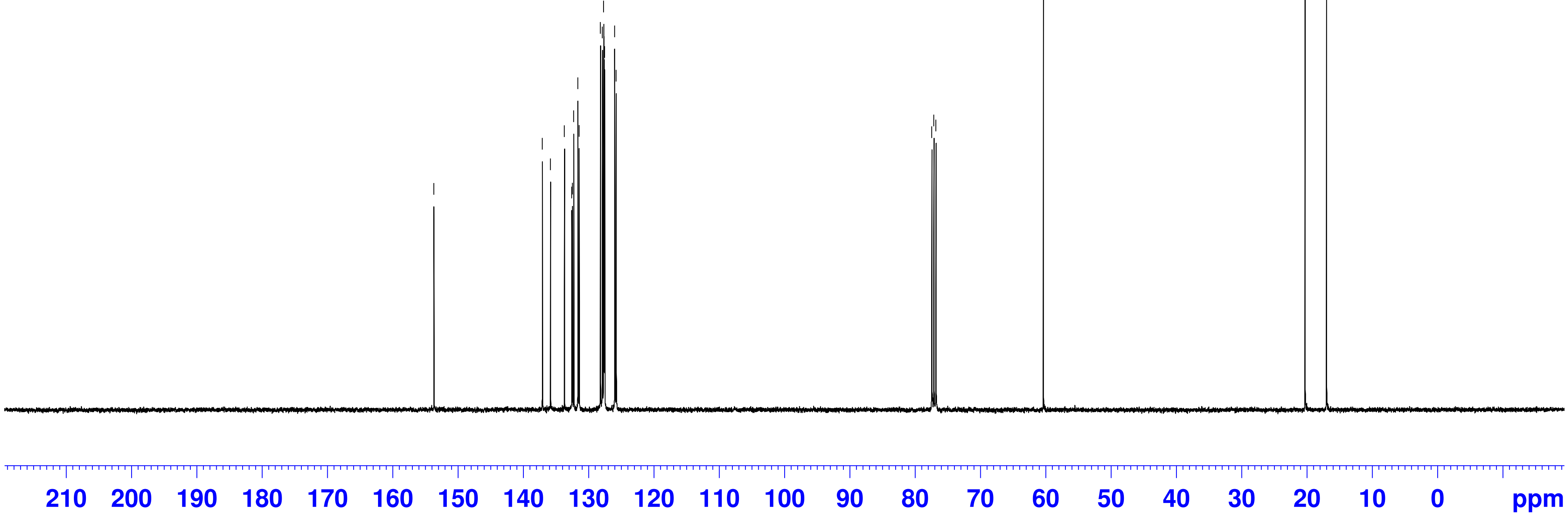


```
NAME     TJ-20140411-E-827-2-naph-cpl-chiral-prod
EXPNO              11
PROCNO              1
Date_        20140411
Time            11.09
INSTRUM         spect
PROBHD   5 mm PABBO BB/
PULPROG        zgpg30
TD              65536
SOLVENT         CDCl3
NS                256
DS                  4
SWH         24038.461 Hz
FIDRES       0.366798 Hz
AQ          1.3631988 sec
RG              191.9
DW             20.800 usec
DE               6.50 usec
TE              298.2 K
D1         2.00000000 sec
D11        0.03000000 sec
TD0                 1

======== CHANNEL f1 ========
SFO1      100.6479769 MHz
NUC1              13C
P1               9.20 usec
SI              32768
SF        100.6379112 MHz
WDW                EM
SSB                 0
LB               1.00 Hz
GB                  0
PC               1.40
```

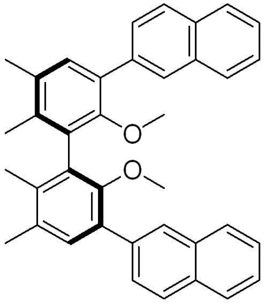

S6

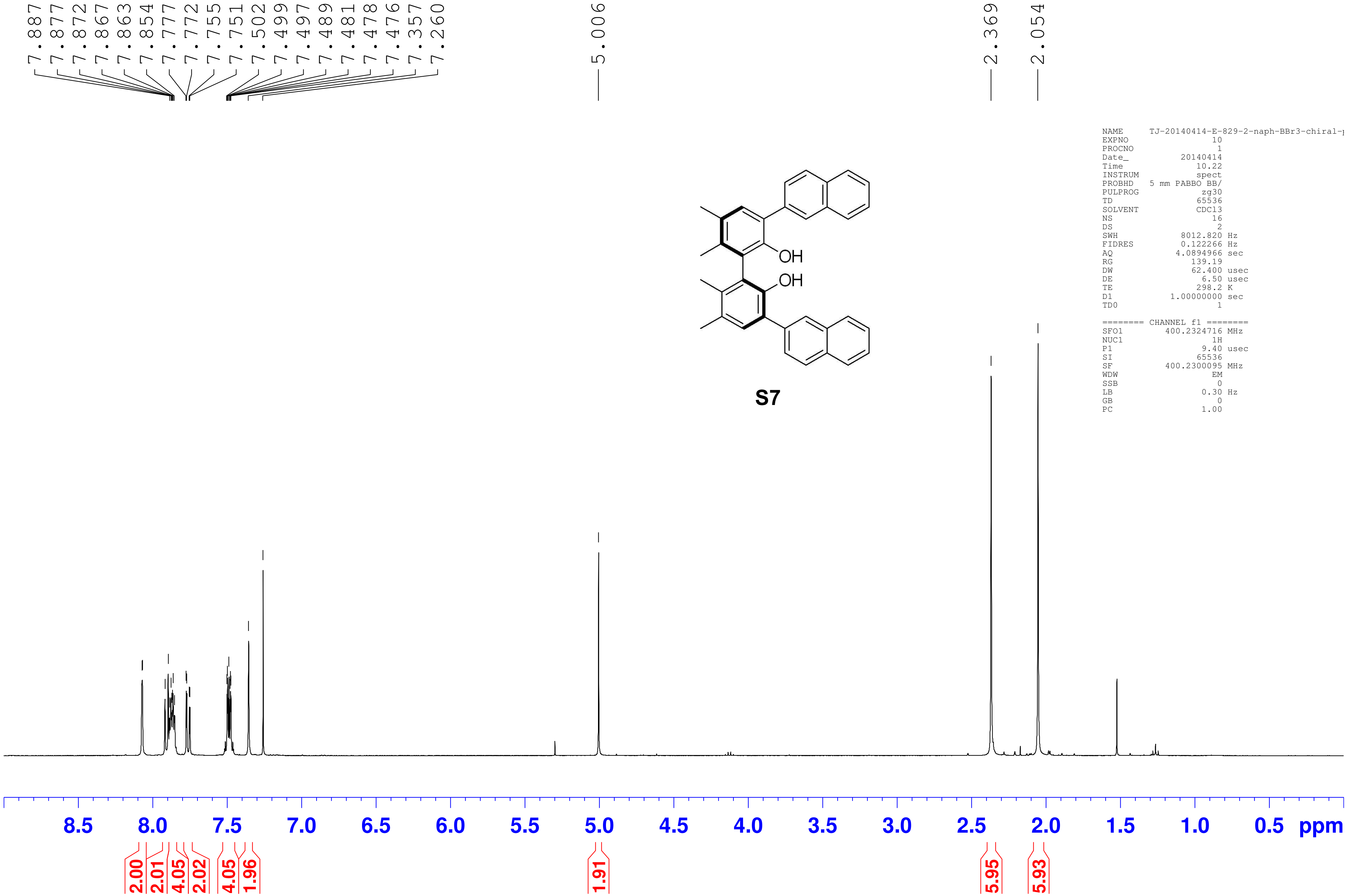
7.887
7.877
7.872
7.867
7.863
7.854
7.777
7.772
7.755
7.751
7.502
7.499
7.497
7.489
7.481
7.478
7.476
7.357
7.260
5.006
2.369
2.054
NAME TJ-20140414-E-829-2-naph-BBr3-chiral-
EXPNO 10
PROCNO 1
Date_ 20140414
Time 10.22
INSTRUM spect
PROBHD 5 mm PABBO BB/
PULPROG zg30
TD 65536
SOLVENT CDCl3
NS 16
DS 2
SWH 8012.820 Hz
FIDRES 0.122266 Hz
AQ 4.0894966 sec
RG 139.19
DW 62.400 usec
DE 6.50 usec
TE 298.2 K
D1 1.00000000 sec
TD0 1
======== CHANNEL f1 ========
SFO1 400.2324716 MHz
NUC1 1H
P1 9.40 usec
SI 65536
SF 400.2300095 MHz
WDW EM
SSB 0
LB 0.30 Hz
GB 0
PC 1.00
8.5
8.0
7.5
7.0
6.5
6.0
5.5
5.0
4.5
4.0
3.5
3.0
2.5
2.0
1.5
1.0
0.5
ppm
2.00
2.01
4.05
2.02
4.05
1.96
1.91
5.95
5.93
OH
OH

S7

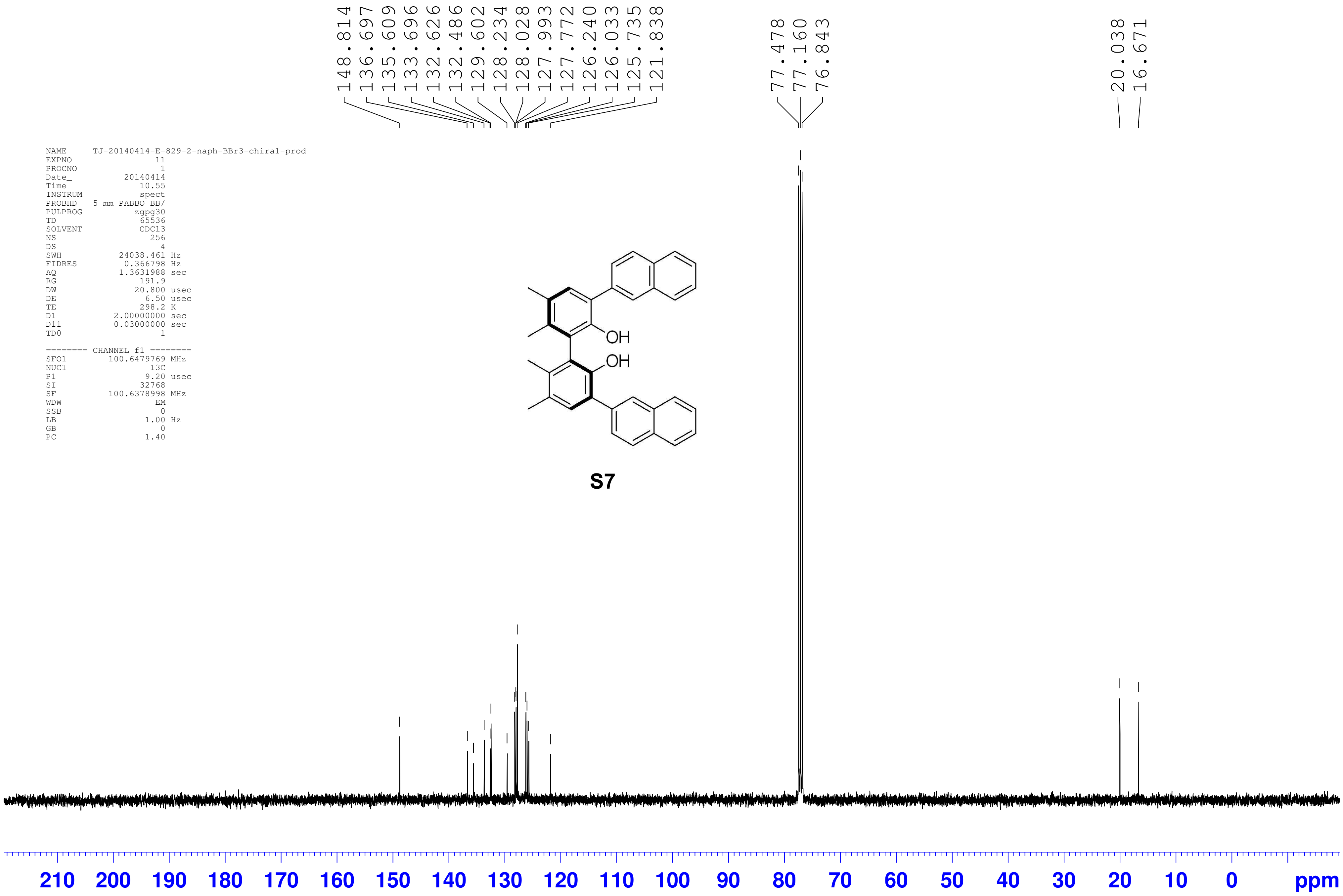
148.814
136.697
135.609
133.696
132.626
132.486
129.602
128.234
128.028
127.993
127.772
126.240
126.033
125.735
121.838
77.478
77.160
76.843
20.038
16.671
NAME TJ-20140414-E-829-2-naph-BBr3-chiral-prod
EXPNO 11
PROCNO 1
Date_ 20140414
Time 10.55
INSTRUM spect
PROBHD 5 mm PABBO BB/
PULPROG zgpg30
TD 65536
SOLVENT CDCl3
NS 256
DS 4
SWH 24038.461 Hz
FIDRES 0.366798 Hz
AQ 1.3631988 sec
RG 191.9
DW 20.800 usec
DE 6.50 usec
TE 298.2 K
D1 2.00000000 sec
D11 0.03000000 sec
TD0 1
======== CHANNEL f1 ========
SFO1 100.6479769 MHz
NUC1 13C
P1 9.20 usec
SI 32768
SF 100.6378998 MHz
WDW EM
SSB 0
LB 1.00 Hz
GB 0
PC 1.40
OH
OH
210 200 190 180 170 160 150 140 130 120 110 100 90 80 70 60 50 40 30 20 10 0 ppm

S7

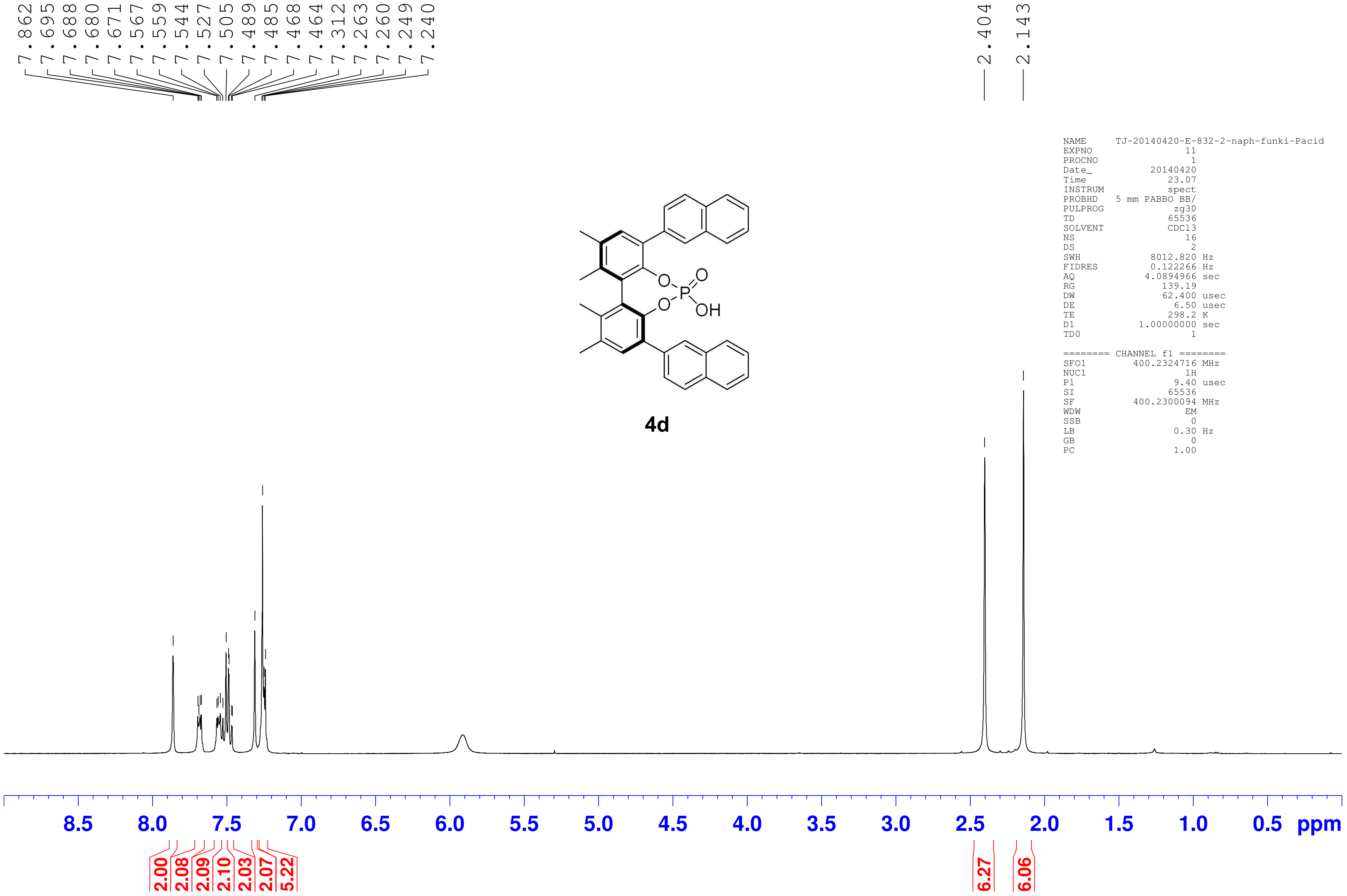
7.862
7.695
7.688
7.680
7.671
7.567
7.559
7.544
7.527
7.505
7.489
7.485
7.468
7.464
7.312
7.263
7.260
7.249
7.240
2.404
2.143
NAME TJ-20140420-E-832-2-naph-funki-Pacid
EXPNO 11
PROCNO 1
Date_ 20140420
Time 23.07
INSTRUM spect
PROBHD 5 mm PABBO BB/
PULPROG zg30
TD 65536
SOLVENT CDCl3
NS 16
DS 2
SWH 8012.820 Hz
FIDRES 0.122266 Hz
AQ 4.0894966 sec
RG 139.19
DW 62.400 usec
DE 6.50 usec
TE 298.2 K
D1 1.00000000 sec
TD0 1
======== CHANNEL f1 ========
SFO1 400.2324716 MHz
NUC1 1H
P1 9.40 usec
SI 65536
SF 400.2300094 MHz
WDW EM
SSB 0
LB 0.30 Hz
GB 0
PC 1.00
8.5
8.0
7.5
7.0
6.5
6.0
5.5
5.0
4.5
4.0
3.5
3.0
2.5
2.0
1.5
1.0
0.5
ppm
2.00
2.08
2.09
2.10
2.03
2.07
5.22
6.27
6.06
O
P
O
O
OH

4d

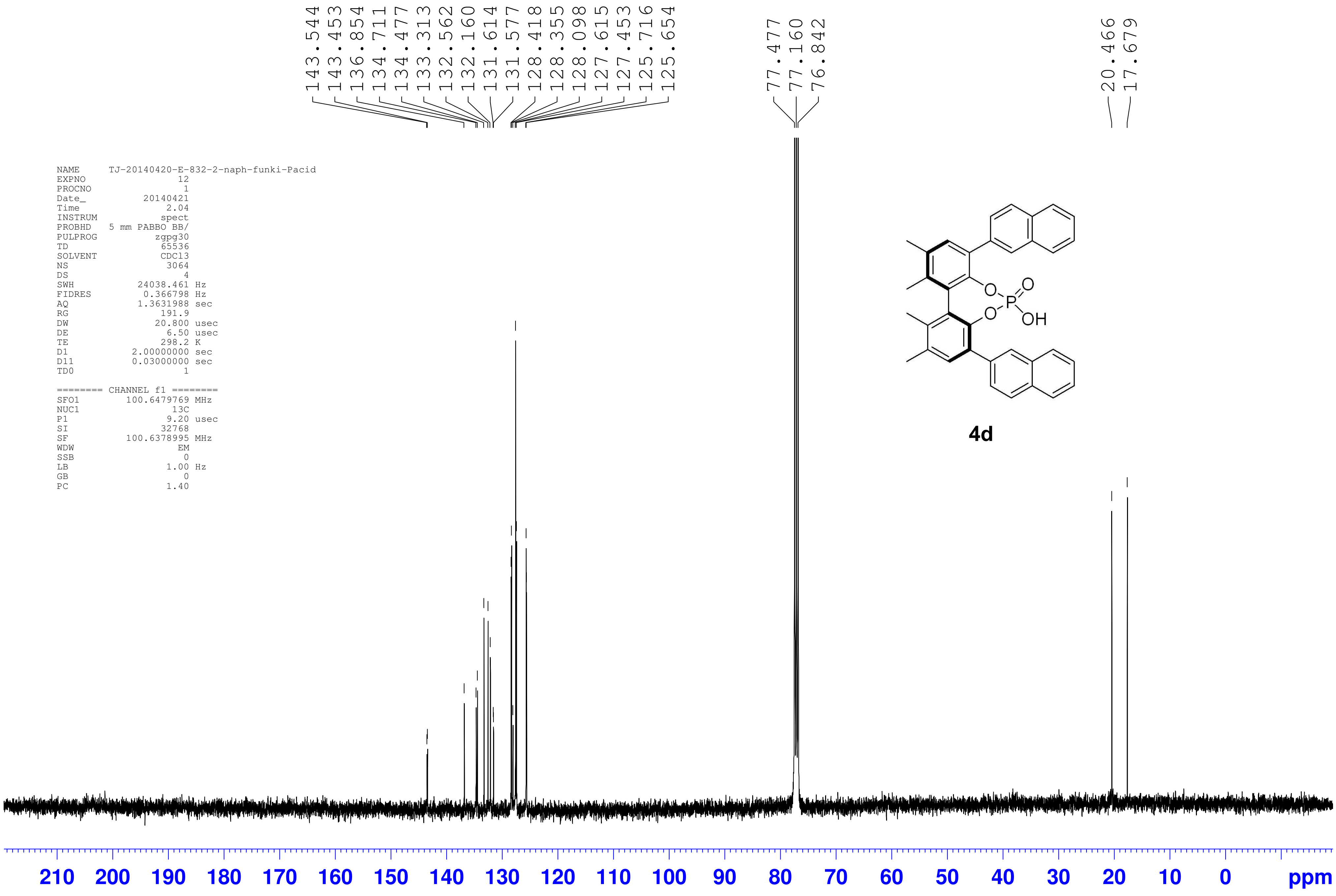
143.544
143.453
136.854
134.711
134.477
133.313
132.562
132.160
131.614
131.577
128.418
128.355
128.098
127.615
127.453
125.716
125.654
77.477
77.160
76.842
20.466
17.679
NAME TJ-20140420-E-832-2-naph-funki-Pacid
EXPNO 12
PROCNO 1
Date_ 20140421
Time 2.04
INSTRUM spect
PROBHD 5 mm PABBO BB/
PULPROG zgpg30
TD 65536
SOLVENT CDCl3
NS 3064
DS 4
SWH 24038.461 Hz
FIDRES 0.366798 Hz
AQ 1.3631988 sec
RG 191.9
DW 20.800 usec
DE 6.50 usec
TE 298.2 K
D1 2.00000000 sec
D11 0.03000000 sec
TD0 1
======== CHANNEL f1 ========
SFO1 100.6479769 MHz
NUC1 13C
P1 9.20 usec
SI 32768
SF 100.6378995 MHz
WDW EM
SSB 0
LB 1.00 Hz
GB 0
PC 1.40
210 200 190 180 170 160 150 140 130 120 110 100 90 80 70 60 50 40 30 20 10 0 ppm
O
P
O
O
OH

4d

```
NAME      TJ-20140825-E-850-funki-PNHTf-prod
EXPNO                 10
PROCNO                 1
Date_           20140825
Time               19.50
INSTRUM            spect
PROBHD    5 mm PABBO BB/
PULPROG             zg30
TD                 65536
SOLVENT            CDCl3
NS                    16
DS                     2
SWH             8012.820 Hz
FIDRES          0.122266 Hz
AQ             4.0894966 sec
RG                 69.35
DW                62.400 usec
DE                  6.50 usec
TE                 299.8 K
D1            1.00000000 sec
TD0                    1

======== CHANNEL f1 ========
SFO1         400.2424716 MHz
NUC1                  1H
P1                  9.40 usec
SI                 65536
SF           400.2400095 MHz
WDW                   EM
SSB                    0
LB                  0.30 Hz
GB                     0
PC                  1.00
```

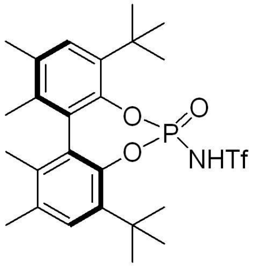

4e

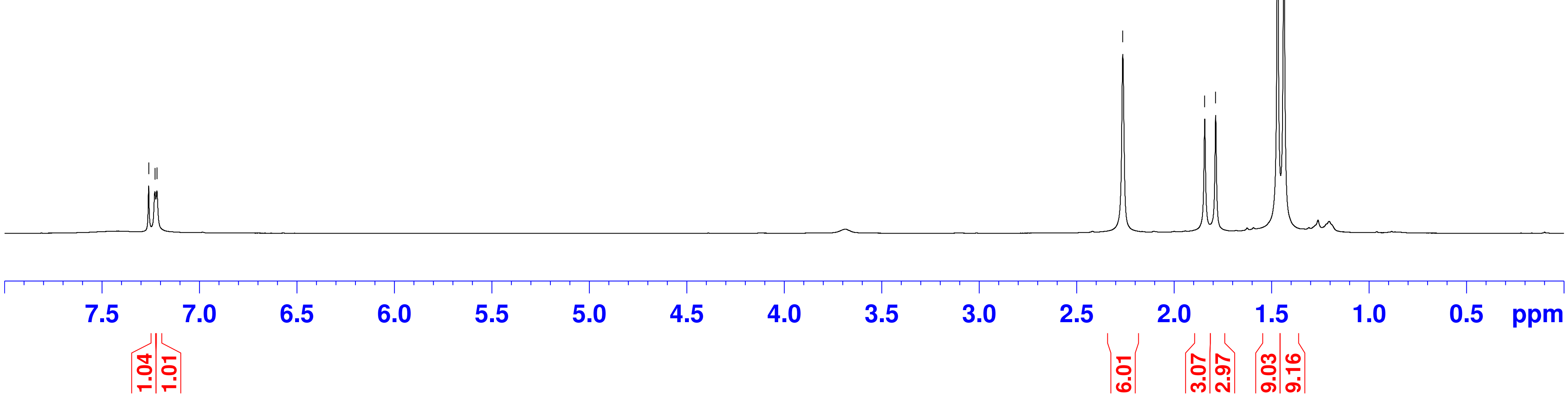

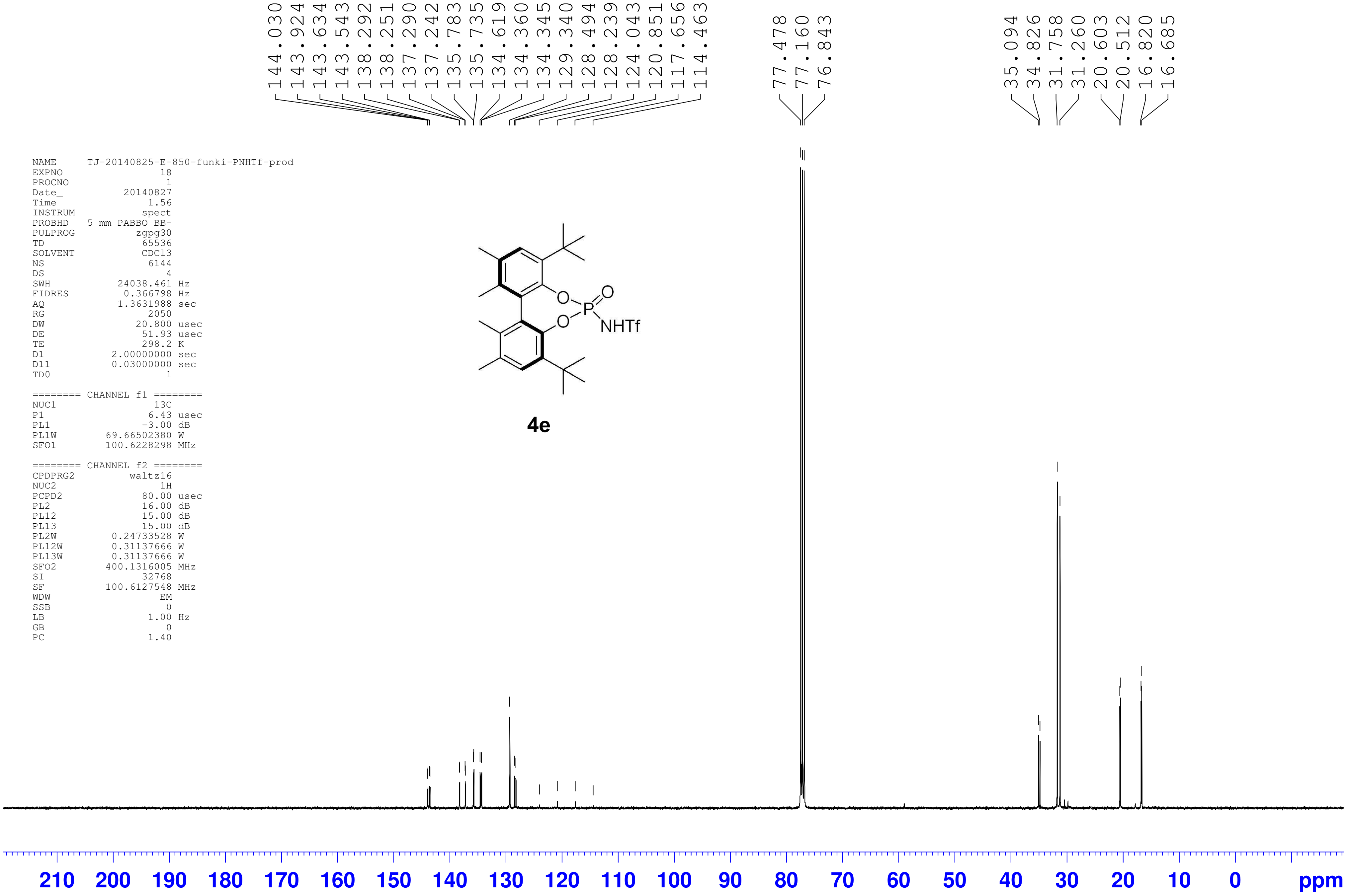


```
NAME       TJ-20140825-E-850-funki-PNHTf-prod
EXPNO              18
PROCNO              1
Date_        20140827
Time             1.56
INSTRUM         spect
PROBHD   5 mm PABBO BB-
PULPROG        zgpg30
TD              65536
SOLVENT         CDCl3
NS               6144
DS                  4
SWH         24038.461 Hz
FIDRES       0.366798 Hz
AQ          1.3631988 sec
RG               2050
DW             20.800 usec
DE              51.93 usec
TE              298.2 K
D1         2.00000000 sec
D11        0.03000000 sec
TD0                 1

======== CHANNEL f1 ========
NUC1              13C
P1               6.43 usec
PL1             -3.00 dB
PL1W      69.66502380 W
SFO1      100.6228298 MHz

======== CHANNEL f2 ========
CPDPRG2       waltz16
NUC2               1H
PCPD2           80.00 usec
PL2             16.00 dB
PL12            15.00 dB
PL13            15.00 dB
PL2W       0.24733528 W
PL12W      0.31137666 W
PL13W      0.31137666 W
SFO2      400.1316005 MHz
SI              32768
SF        100.6127548 MHz
WDW                EM
SSB                 0
LB               1.00 Hz
GB                  0
PC               1.40
```

4e

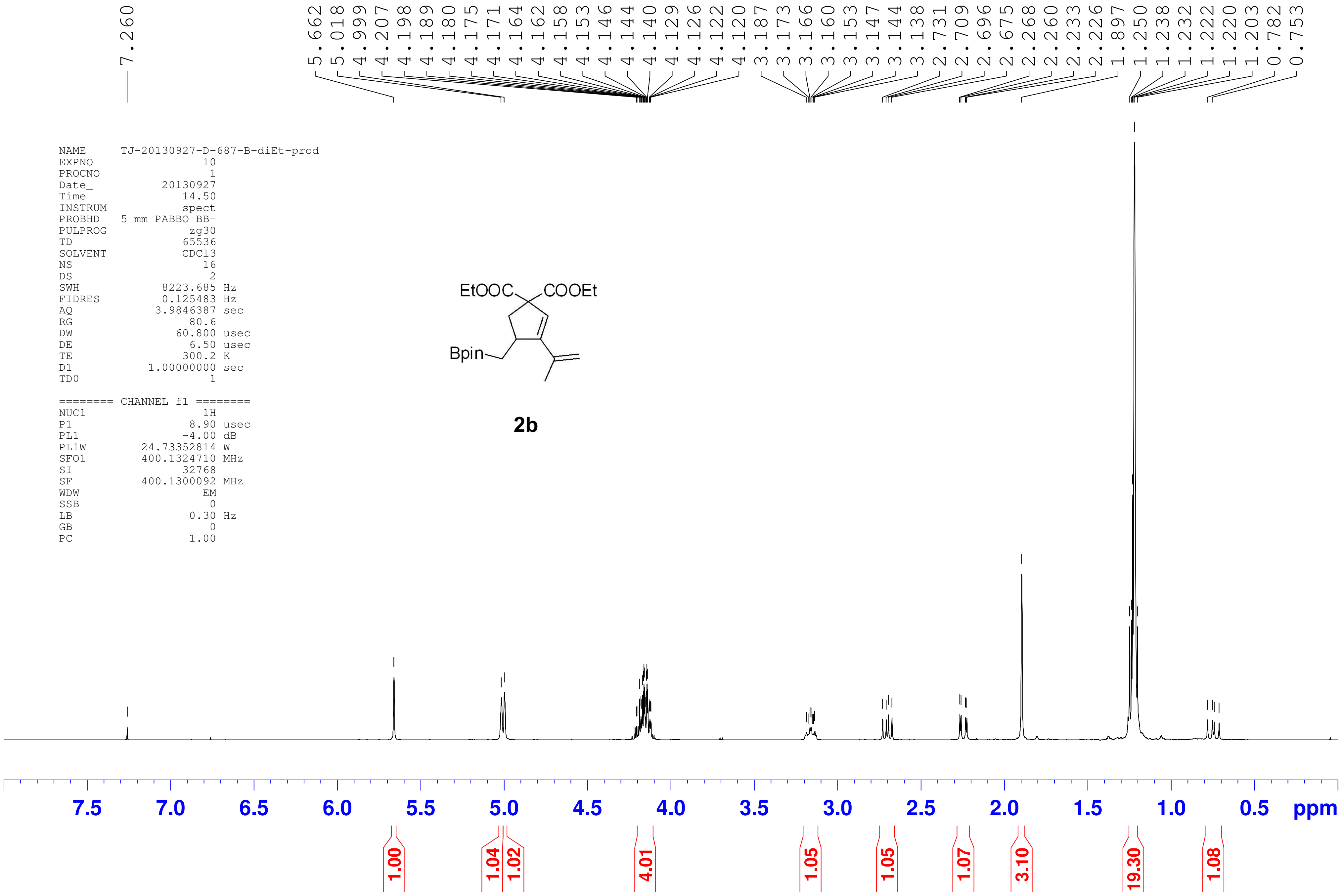


```
NAME     TJ-20130927-D-687-B-diEt-prod
EXPNO                 10
PROCNO                 1
Date_           20130927
Time               14.50
INSTRUM            spect
PROBHD   5 mm PABBO BB-
PULPROG             zg30
TD                 65536
SOLVENT            CDCl3
NS                    16
DS                     2
SWH             8223.685 Hz
FIDRES          0.125483 Hz
AQ             3.9846387 sec
RG                  80.6
DW                60.800 usec
DE                  6.50 usec
TE                 300.2 K
D1            1.00000000 sec
TD0                    1

======== CHANNEL f1 ========
NUC1                  1H
P1                  8.90 usec
PL1                -4.00 dB
PL1W         24.73352814 W
SFO1         400.1324710 MHz
SI                 32768
SF           400.1300092 MHz
WDW                   EM
SSB                    0
LB                  0.30 Hz
GB                     0
PC                  1.00
```

2b

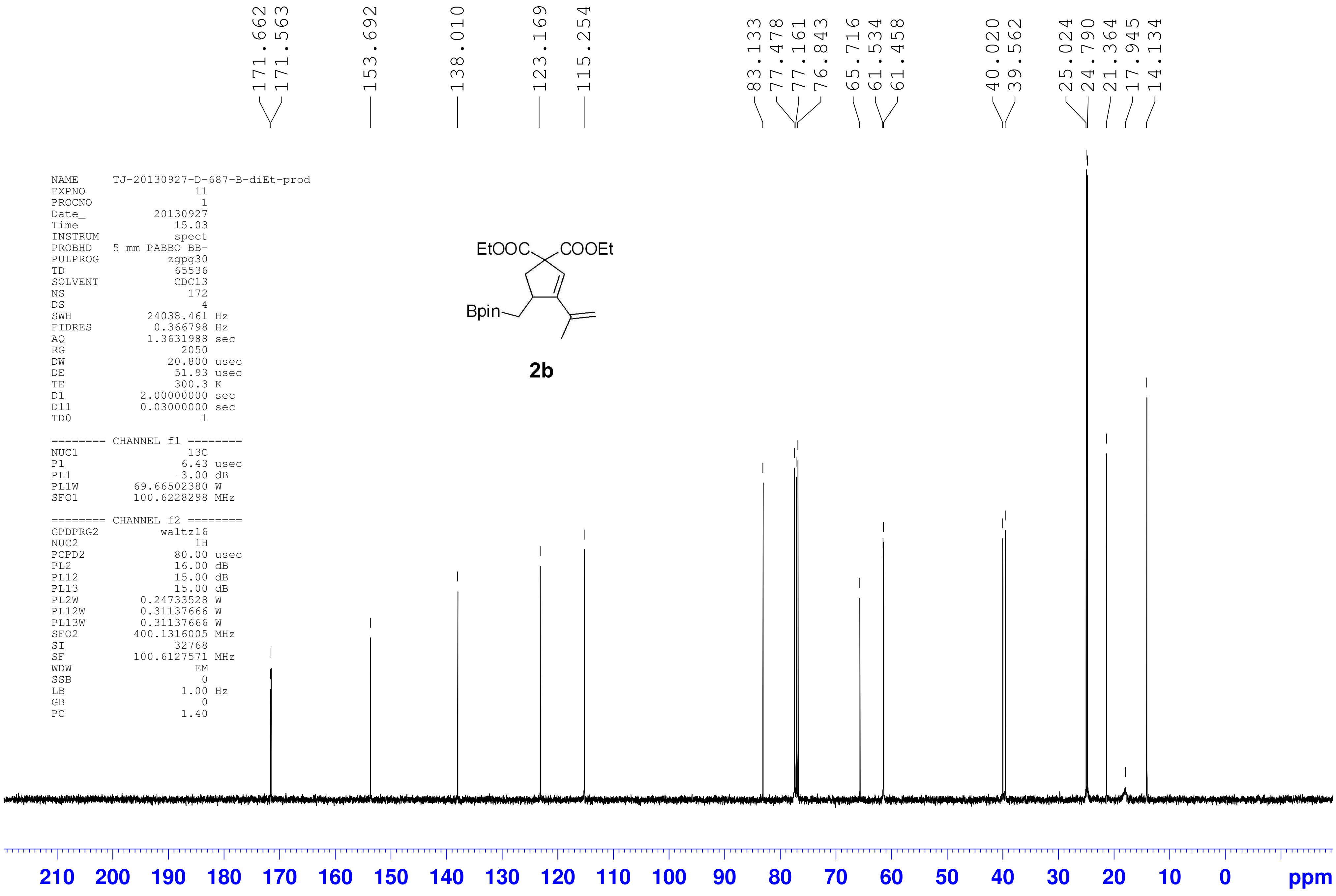

NAME	TJ-20130927-D-687-B-diEt-prod	
EXPNO	11	
PROCNO	1	
Date_	20130927	
Time	15.03	
INSTRUM	spect	
PROBHD	5 mm PABBO BB-	
PULPROG	zgpg30	
TD	65536	
SOLVENT	CDCl3	
NS	172	
DS	4	
SWH	24038.461	Hz
FIDRES	0.366798	Hz
AQ	1.3631988	sec
RG	2050	
DW	20.800	usec
DE	51.93	usec
TE	300.3	K
D1	2.00000000	sec
D11	0.03000000	sec
TD0	1	
======== CHANNEL f1 ========		
NUC1	13C	
P1	6.43	usec
PL1	-3.00	dB
PL1W	69.66502380	W
SFO1	100.6228298	MHz
======== CHANNEL f2 ========		
CPDPRG2	waltz16	
NUC2	1H	
PCPD2	80.00	usec
PL2	16.00	dB
PL12	15.00	dB
PL13	15.00	dB
PL2W	0.24733528	W
PL12W	0.31137666	W
PL13W	0.31137666	W
SFO2	400.1316005	MHz
SI	32768	
SF	100.6127571	MHz
WDW	EM	
SSB	0	
LB	1.00	Hz
GB	0	
PC	1.40	

2b

```
NAME     TJ-20140803-E-981-cy-enallene-B-prod
EXPNO                10
PROCNO                1
Date_          20140803
Time              16.26
INSTRUM           spect
PROBHD   5 mm PABBO BB-
PULPROG            zg30
TD                65536
SOLVENT           CDCl3
NS                   16
DS                    2
SWH            8223.685 Hz
FIDRES         0.125483 Hz
AQ            3.9846387 sec
RG                  144
DW               60.800 usec
DE                 6.50 usec
TE                301.0 K
D1           1.00000000 sec
TD0                   1

======== CHANNEL f1 ========
NUC1                 1H
P1                 8.90 usec
PL1               -4.00 dB
PL1W        24.73352814 W
SFO1        400.1324710 MHz
SI                32768
SF          400.1300093 MHz
WDW                  EM
SSB                   0
LB                 0.30 Hz
GB                    0
PC                 1.00
```

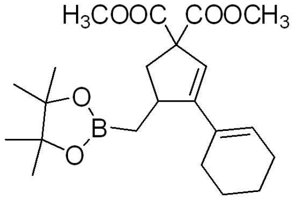

2c

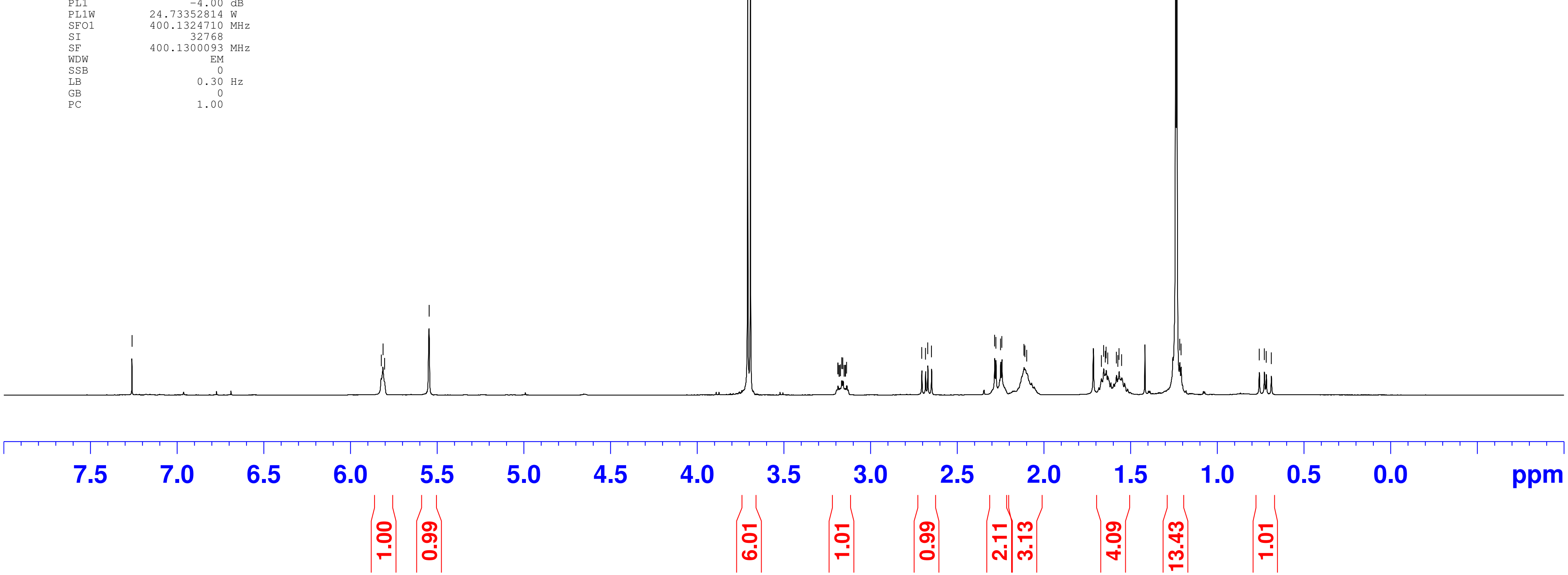

```
NAME     TJ-20140803-E-981-cy-enallene-B-prod
EXPNO               11
PROCNO               1
Date_         20140803
Time             16.32
INSTRUM          spect
PROBHD   5 mm PABBO BB-
PULPROG        zgpg30
TD               65536
SOLVENT          CDCl3
NS                  64
DS                   4
SWH          24038.461 Hz
FIDRES        0.366798 Hz
AQ           1.3631988 sec
RG                2050
DW              20.800 usec
DE               51.93 usec
TE               301.9 K
D1          2.00000000 sec
D11         0.03000000 sec
TD0                  1

======== CHANNEL f1 ========
NUC1               13C
P1                6.43 usec
PL1              -3.00 dB
PL1W       69.66502380 W
SFO1       100.6228298 MHz

======== CHANNEL f2 ========
CPDPRG2        waltz16
NUC2                1H
PCPD2            80.00 usec
PL2              16.00 dB
PL12             15.00 dB
PL13             15.00 dB
PL2W        0.24733528 W
PL12W       0.31137666 W
PL13W       0.31137666 W
SFO2       400.1316005 MHz
SI               32768
SF         100.6127553 MHz
WDW                 EM
SSB                  0
LB                1.00 Hz
GB                   0
PC                1.40
```

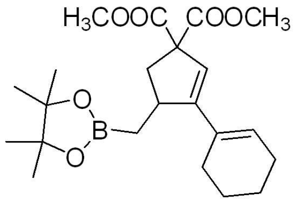

2c

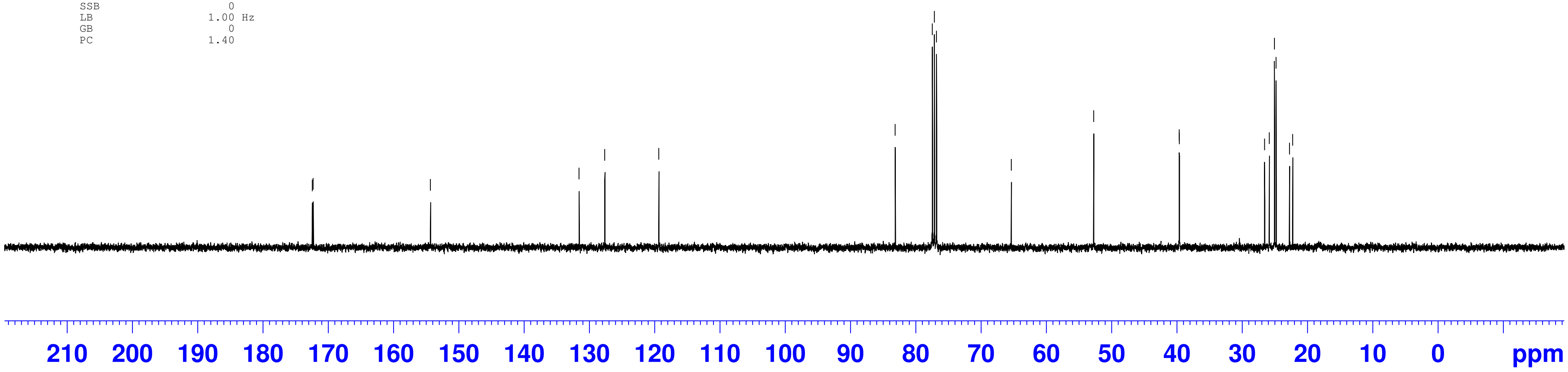

7.260
5.800
5.796
5.793
5.791
5.516
3.720
3.702
3.184
3.175
3.171
3.163
3.157
3.146
3.136
3.134
2.762
2.740
2.727
2.706
2.533
2.528
2.511
2.507
2.489
2.466
2.462
2.454
2.450
2.445
2.440
2.432
2.428
2.410
2.391
2.277
2.268
2.242
2.234
1.905
1.898
1.890
1.886
1.884
1.877
1.869
1.862
1.851
1.847
1.317
1.308
1.276
1.268
1.243
1.235
0.807
0.778
0.767
0.738

NAME	TJ-20140809-E-993-cyclopentyl-B-prod
EXPNO	10
PROCNO	1
Date_	20140809
Time	12.06
INSTRUM	spect
PROBHD	5 mm PABBO BB-
PULPROG	zg30
TD	65536
SOLVENT	CDCl3
NS	16
DS	2
SWH	8223.685 Hz
FIDRES	0.125483 Hz
AQ	3.9846387 sec
RG	181
DW	60.800 usec
DE	6.50 usec
TE	298.2 K
D1	1.00000000 sec
TD0	1

======== CHANNEL f1 ========

NUC1	1H
P1	8.90 usec
PL1	-4.00 dB
PL1W	24.73352814 W
SFO1	400.1324710 MHz
SI	32768
SF	400.1300094 MHz
WDW	EM
SSB	0
LB	0.30 Hz
GB	0
PC	1.00

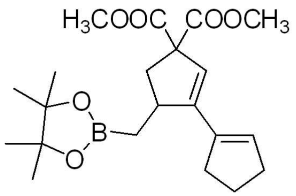

2d

7.5 7.0 6.5 6.0 5.5 5.0 4.5 4.0 3.5 3.0 2.5 2.0 1.5 1.0 0.5 ppm

1.00 0.99 3.00 2.98 0.99 1.00 4.11 1.05 2.02 1.17 11.98 1.01

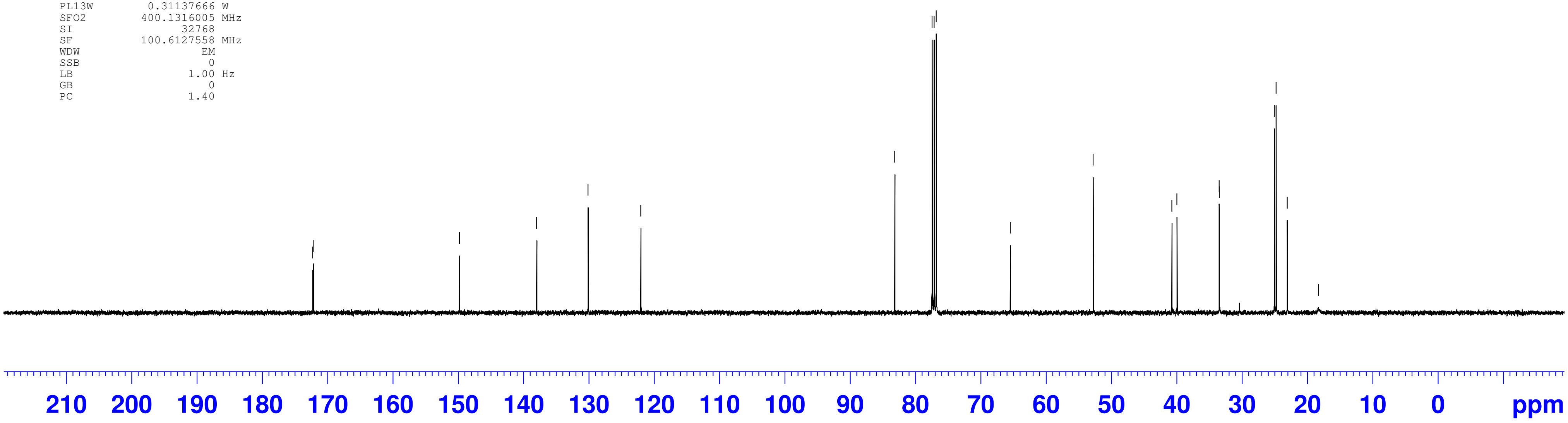


```
NAME     TJ-20140809-E-993-cyclopentyl-B-prod
EXPNO                 11
PROCNO                 1
Date_           20140809
Time               12.36
INSTRUM            spect
PROBHD   5 mm PABBO BB-
PULPROG          zgpg30
TD                 65536
SOLVENT            CDCl3
NS                   360
DS                     4
SWH            24038.461 Hz
FIDRES          0.366798 Hz
AQ             1.3631988 sec
RG                  2050
DW                20.800 usec
DE                 51.93 usec
TE                 298.2 K
D1            2.00000000 sec
D11           0.03000000 sec
TD0                    1

======== CHANNEL f1 ========
NUC1                 13C
P1                  6.43 usec
PL1                -3.00 dB
PL1W         69.66502380 W
SFO1         100.6228298 MHz

======== CHANNEL f2 ========
CPDPRG2          waltz16
NUC2                  1H
PCPD2              80.00 usec
PL2                16.00 dB
PL12               15.00 dB
PL13               15.00 dB
PL2W          0.24733528 W
PL12W         0.31137666 W
PL13W         0.31137666 W
SFO2         400.1316005 MHz
SI                 32768
SF           100.6127558 MHz
WDW                   EM
SSB                    0
LB                  1.00 Hz
GB                     0
PC                  1.40
```

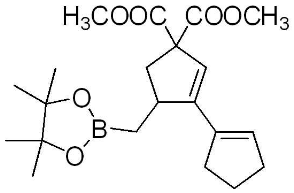

2d

NAME TJ-20140818-E-1002-ch-B-prod-re
EXPNO 10
PROCNO 1
Date_ 20140818
Time 16.29
INSTRUM spect
PROBHD 5 mm PABBO BB-
PULPROG zg30
TD 65536
SOLVENT CDCl3
NS 16
DS 2
SWH 8223.685 Hz
FIDRES 0.125483 Hz
AQ 3.9846387 sec
RG 181
DW 60.800 usec
DE 6.50 usec
TE 298.2 K
D1 1.00000000 sec
TD0 1

======== CHANNEL f1 ========
NUC1 1H
P1 8.90 usec
PL1 -4.00 dB
PL1W 24.73352814 W
SFO1 400.1324710 MHz
SI 32768
SF 400.1300093 MHz
WDW EM
SSB 0
LB 0.30 Hz
GB 0
PC 1.00

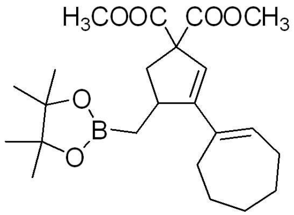

2e

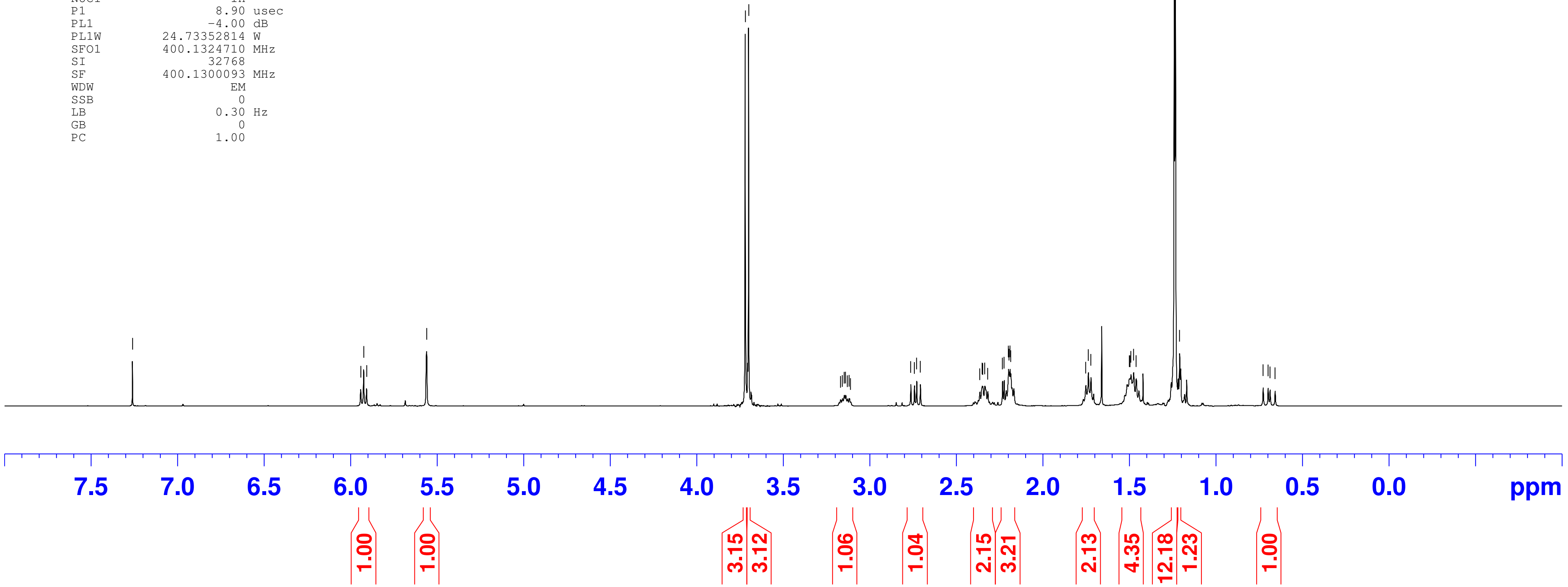

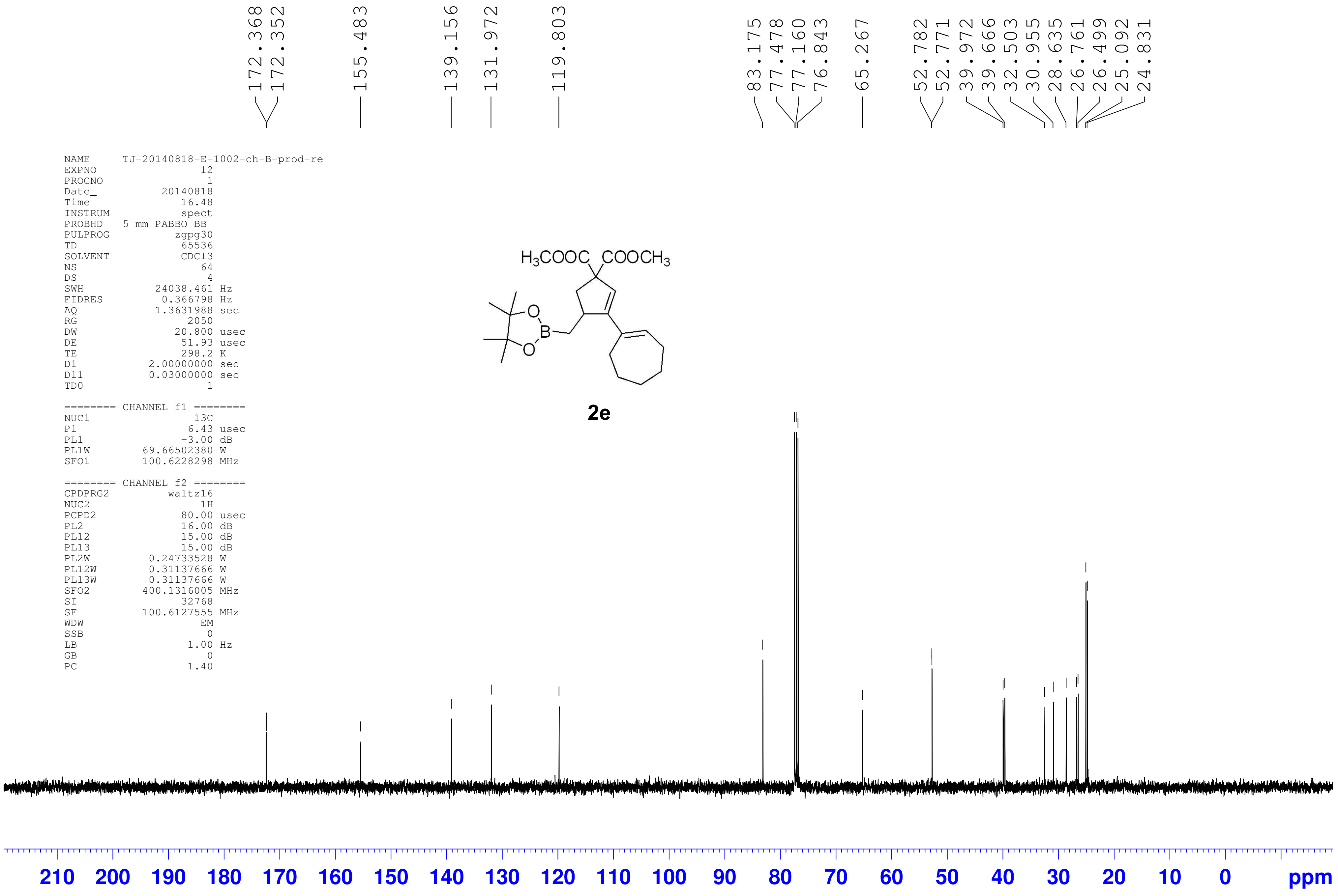
172.368
172.352
155.483
139.156
131.972
119.803
83.175
77.478
77.160
76.843
65.267
52.782
52.771
39.972
39.666
32.503
30.955
28.635
26.761
26.499
25.092
24.831
NAME TJ-20140818-E-1002-ch-B-prod-re
EXPNO 12
PROCNO 1
Date_ 20140818
Time 16.48
INSTRUM spect
PROBHD 5 mm PABBO BB-
PULPROG zgpg30
TD 65536
SOLVENT CDCl3
NS 64
DS 4
SWH 24038.461 Hz
FIDRES 0.366798 Hz
AQ 1.3631988 sec
RG 2050
DW 20.800 usec
DE 51.93 usec
TE 298.2 K
D1 2.00000000 sec
D11 0.03000000 sec
TD0 1
======== CHANNEL f1 ========
NUC1 13C
P1 6.43 usec
PL1 -3.00 dB
PL1W 69.66502380 W
SFO1 100.6228298 MHz
======== CHANNEL f2 ========
CPDPRG2 waltz16
NUC2 1H
PCPD2 80.00 usec
PL2 16.00 dB
PL12 15.00 dB
PL13 15.00 dB
PL2W 0.24733528 W
PL12W 0.31137666 W
PL13W 0.31137666 W
SFO2 400.1316005 MHz
SI 32768
SF 100.6127555 MHz
WDW EM
SSB 0
LB 1.00 Hz
GB 0
PC 1.40
210
200
190
180
170
160
150
140
130
120
110
100
90
80
70
60
50
40
30
20
10
0
ppm
H3COOC
COOCH3
O
B
O

2e

```
NAME      TJ-20140902-E-1015-co-B-rac-prod
EXPNO                 10
PROCNO                 1
Date_           20140902
Time               23.56
INSTRUM            spect
PROBHD   5 mm PABBO BB/
PULPROG             zg30
TD                 65536
SOLVENT            CDCl3
NS                    16
DS                     2
SWH             8012.820 Hz
FIDRES          0.122266 Hz
AQ             4.0894966 sec
RG                 29.53
DW                62.400 usec
DE                  6.50 usec
TE                 298.2 K
D1           1.00000000 sec
TD0                    1

======== CHANNEL f1 ========
SFO1         400.2424716 MHz
NUC1                  1H
P1                  9.40 usec
SI                 65536
SF           400.2400095 MHz
WDW                   EM
SSB                    0
LB                  0.30 Hz
GB                     0
PC                  1.00
```

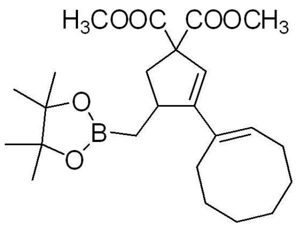

2f

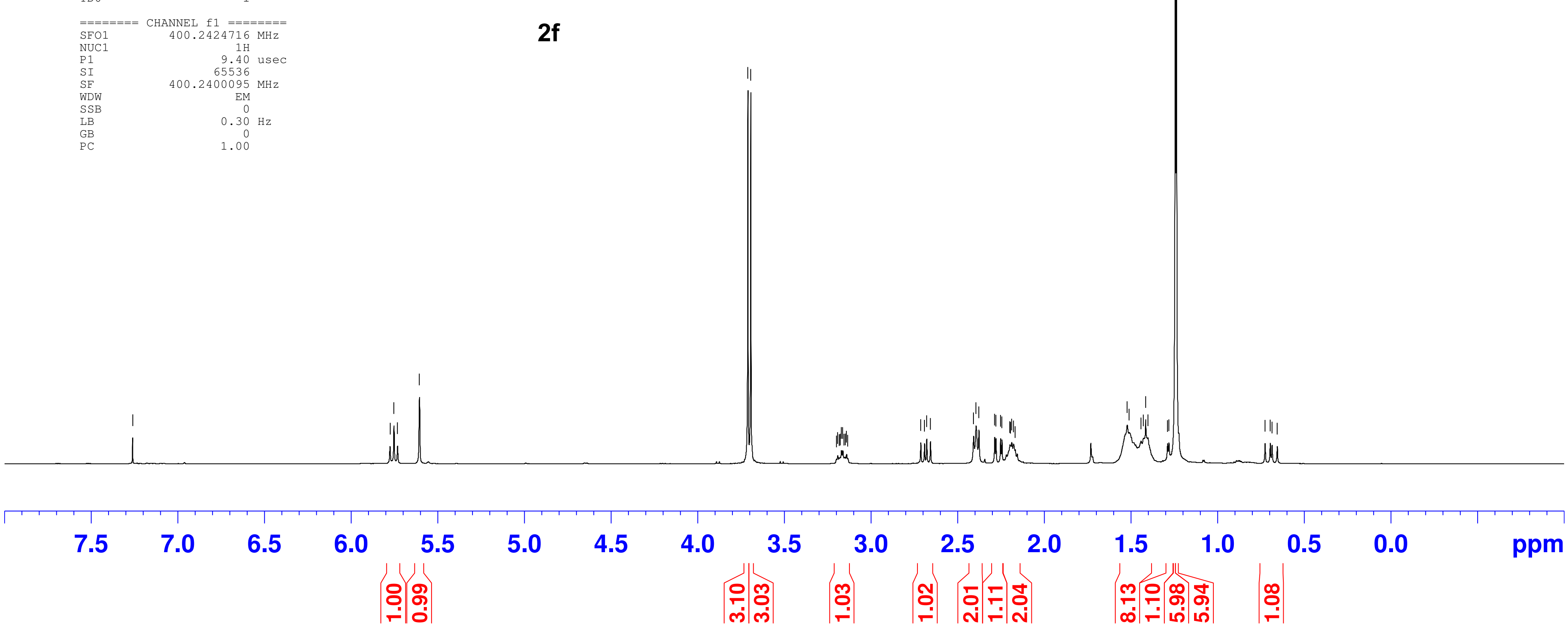

```
NAME      TJ-20140902-E-1015-co-B-rac-prod
EXPNO                   12
PROCNO                   1
Date_             20140903
Time                  0.54
INSTRUM              spect
PROBHD    5 mm PABBO BB/
PULPROG            zgpg30
TD                   65536
SOLVENT              CDCl3
NS                     876
DS                       4
SWH              24038.461 Hz
FIDRES            0.366798 Hz
AQ               1.3631988 sec
RG                   191.9
DW                  20.800 usec
DE                    6.50 usec
TE                   298.2 K
D1              2.00000000 sec
D11             0.03000000 sec
TD0                      1

======== CHANNEL f1 ========
SFO1         100.6504912 MHz
NUC1                   13C
P1                    9.20 usec
SI                   32768
SF           100.6404162 MHz
WDW                     EM
SSB                      0
LB                    1.00 Hz
GB                       0
PC                    1.40
```

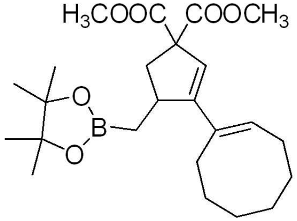

2f

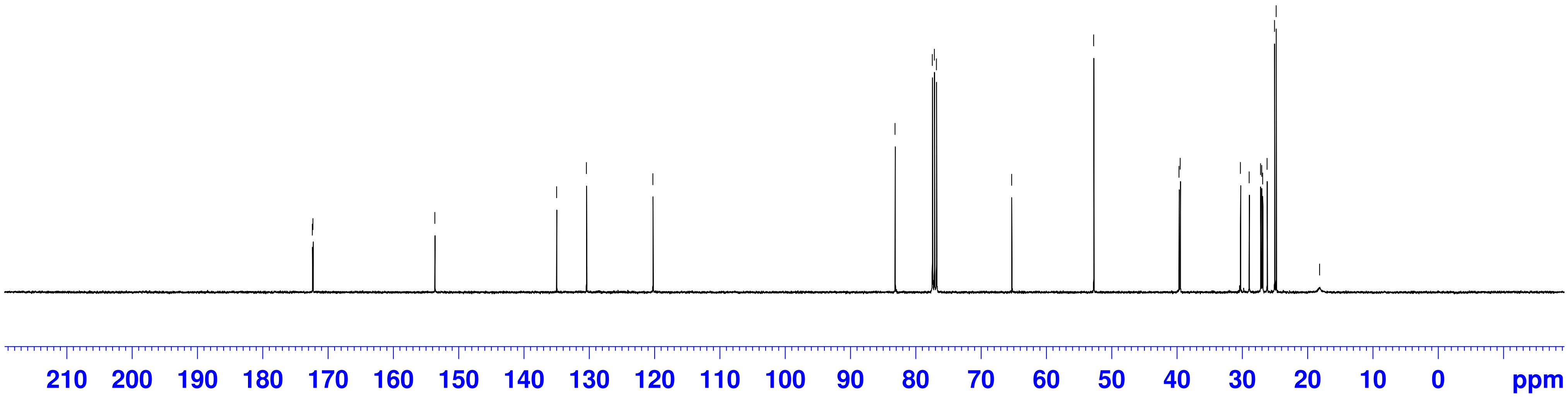

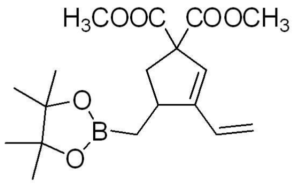

2g

NAME TJ-20140718-E-947-HMe-B-prod-re2
EXPNO 10
PROCNO 1
Date_ 20140718
Time 17.31
INSTRUM spect
PROBHD 5 mm PABBO BB-
PULPROG zg30
TD 65536
SOLVENT CDCl3
NS 16
DS 2
SWH 8223.685 Hz
FIDRES 0.125483 Hz
AQ 3.9846387 sec
RG 203
DW 60.800 usec
DE 6.50 usec
TE 298.2 K
D1 1.00000000 sec
TD0 1

======== CHANNEL f1 ========
NUC1 1H
P1 8.90 usec
PL1 -4.00 dB
PL1W 24.73352814 W
SFO1 400.1324710 MHz
SI 32768
SF 400.1300095 MHz
WDW EM
SSB 0
LB 0.30 Hz
GB 0
PC 1.00

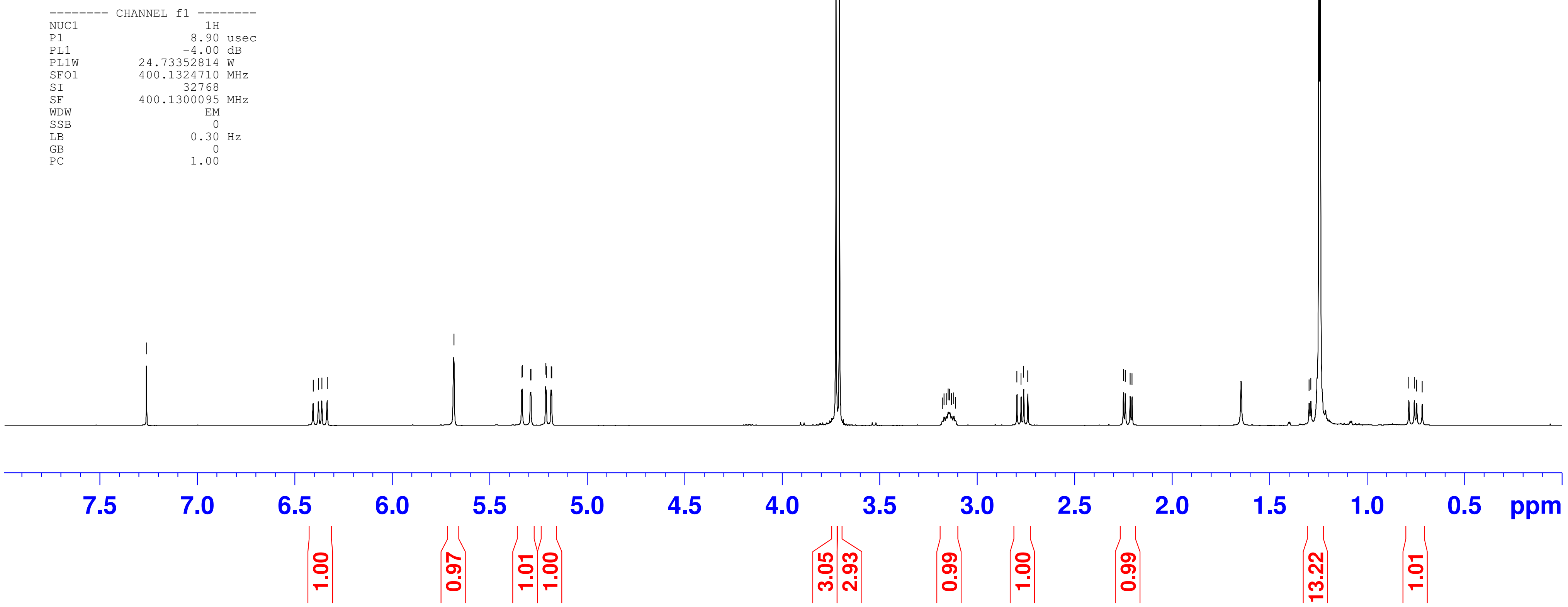

```
NAME     TJ-20140718-E-947-HMe-B-prod-re2
EXPNO                 14
PROCNO                 1
Date_           20140719
Time               12.56
INSTRUM            spect
PROBHD   5 mm PABBO BB-
PULPROG          zgpg30
TD                 65536
SOLVENT            CDCl3
NS                   384
DS                     4
SWH            24038.461 Hz
FIDRES          0.366798 Hz
AQ             1.3631988 sec
RG                  2050
DW                20.800 usec
DE                 51.93 usec
TE                 298.2 K
D1            2.00000000 sec
D11           0.03000000 sec
TD0                    1

======== CHANNEL f1 ========
NUC1                 13C
P1                  6.43 usec
PL1                -3.00 dB
PL1W         69.66502380 W
SFO1         100.6228298 MHz

======== CHANNEL f2 ========
CPDPRG2          waltz16
NUC2                  1H
PCPD2              80.00 usec
PL2                16.00 dB
PL12               15.00 dB
PL13               15.00 dB
PL2W          0.24733528 W
PL12W         0.31137666 W
PL13W         0.31137666 W
SFO2         400.1316005 MHz
SI                 32768
SF           100.6127553 MHz
WDW                   EM
SSB                    0
LB                  1.00 Hz
GB                     0
PC                  1.40
```

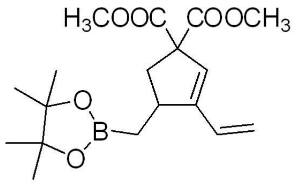

2g

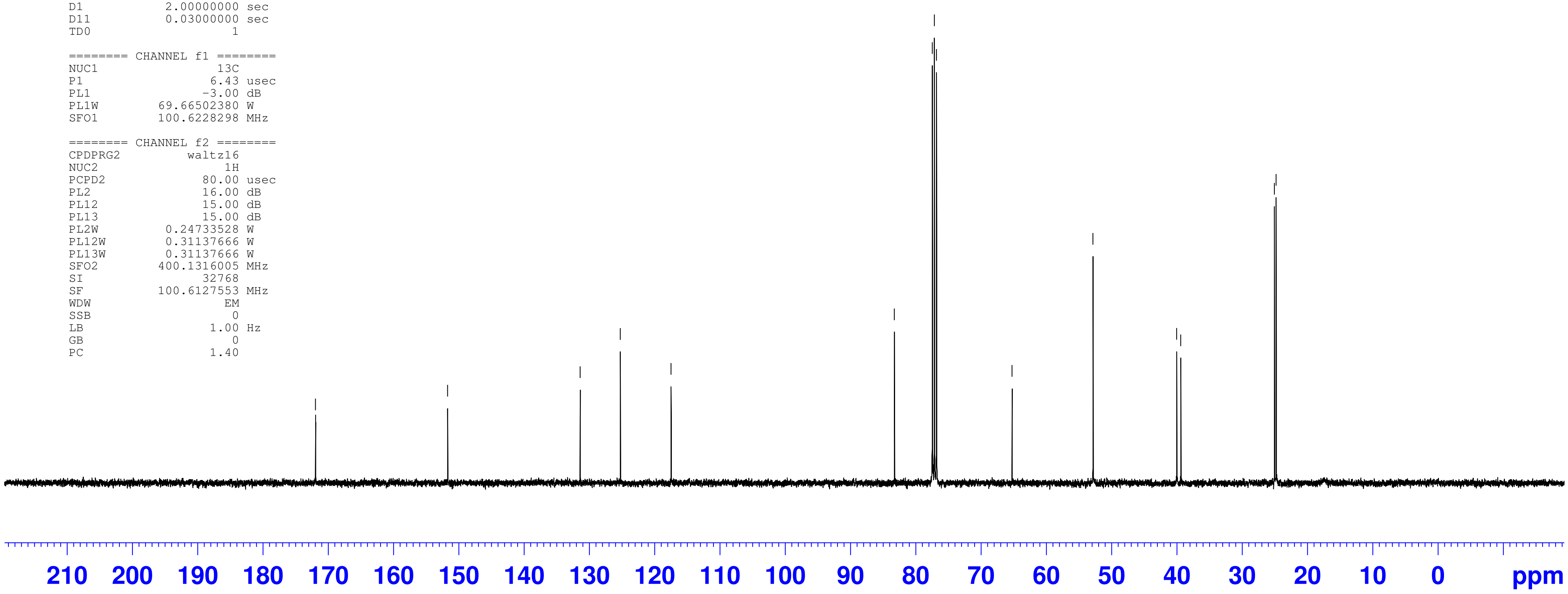

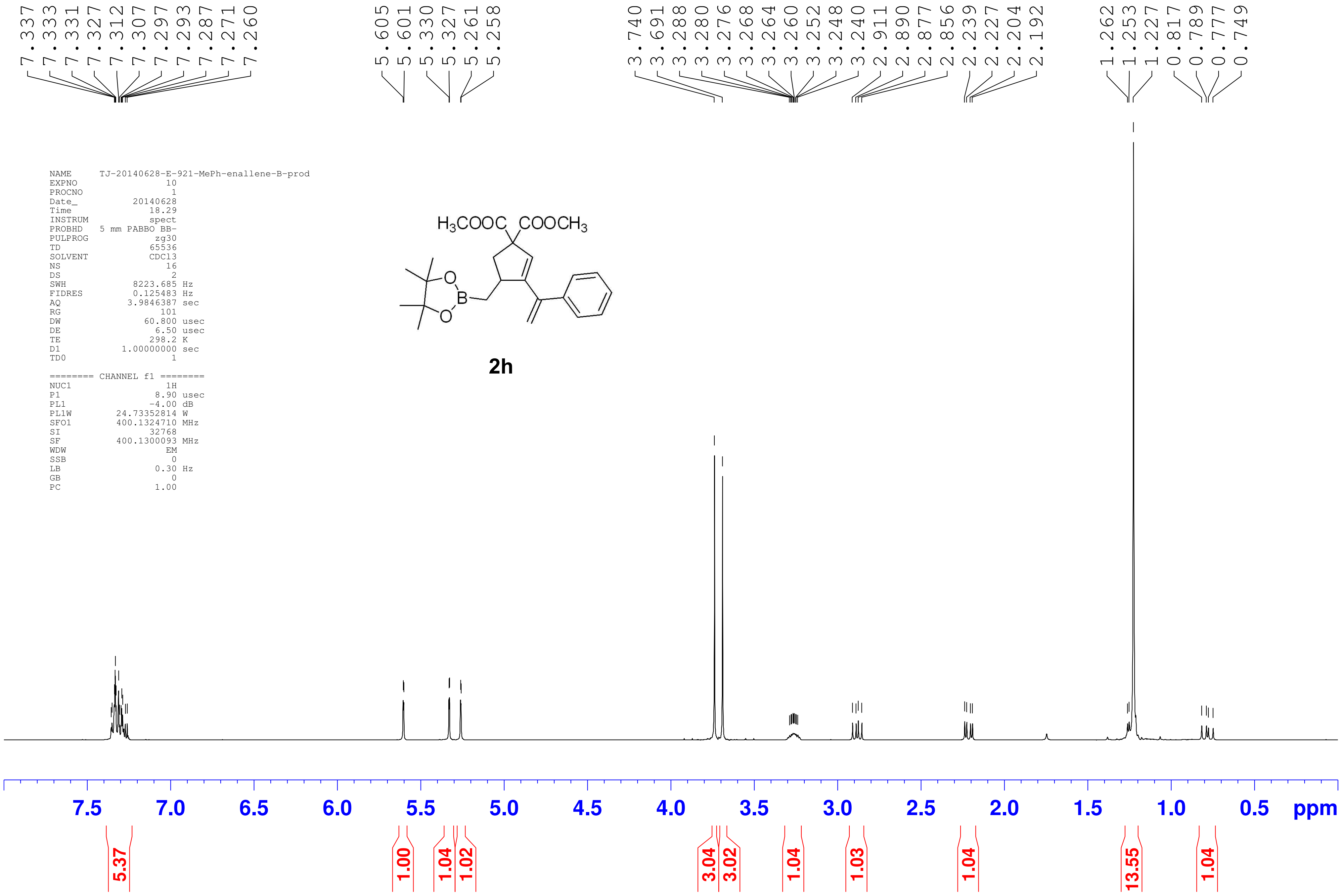
7.337 7.333 7.331 7.327 7.312 7.307 7.297 7.293 7.287 7.271 7.260
5.605 5.601 5.330 5.327 5.261 5.258
3.740 3.691 3.288 3.280 3.276 3.268 3.264 3.260 3.252 3.248 3.240 2.911 2.890 2.877 2.856 2.239 2.227 2.204 2.192
1.262 1.253 1.227 0.817 0.789 0.777 0.749
NAME TJ-20140628-E-921-MePh-enallene-B-prod
EXPNO 10
PROCNO 1
Date_ 20140628
Time 18.29
INSTRUM spect
PROBHD 5 mm PABBO BB-
PULPROG zg30
TD 65536
SOLVENT CDCl3
NS 16
DS 2
SWH 8223.685 Hz
FIDRES 0.125483 Hz
AQ 3.9846387 sec
RG 101
DW 60.800 usec
DE 6.50 usec
TE 298.2 K
D1 1.00000000 sec
TD0 1
======== CHANNEL f1 ========
NUC1 1H
P1 8.90 usec
PL1 -4.00 dB
PL1W 24.73352814 W
SFO1 400.1324710 MHz
SI 32768
SF 400.1300093 MHz
WDW EM
SSB 0
LB 0.30 Hz
GB 0
PC 1.00
H3COOC COOCH3
O
B
O
2h
7.5 7.0 6.5 6.0 5.5 5.0 4.5 4.0 3.5 3.0 2.5 2.0 1.5 1.0 0.5 ppm
5.37 1.00 1.04 1.02 3.04 3.02 1.04 1.03 1.04 13.55 1.04

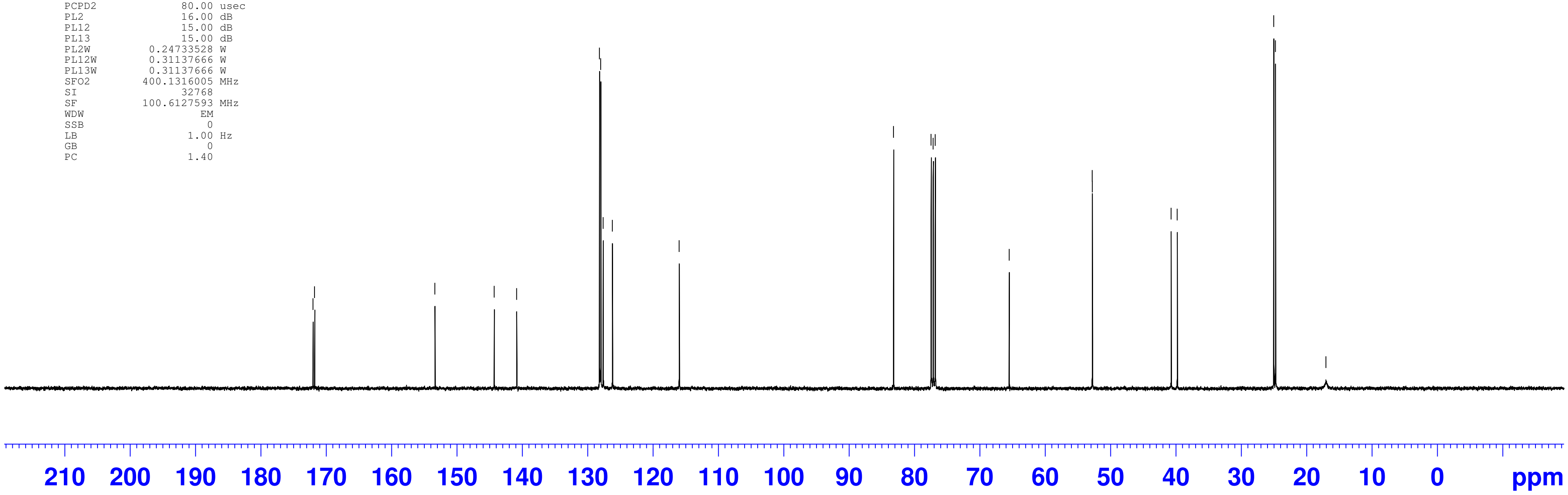


```
NAME     TJ-20140628-E-921-MePh-enallene-B-prod
EXPNO               12
PROCNO               1
Date_         20140629
Time              2.30
INSTRUM          spect
PROBHD   5 mm PABBO BB-
PULPROG         zgpg30
TD               65536
SOLVENT          CDCl3
NS                 384
DS                   4
SWH          24038.461 Hz
FIDRES        0.366798 Hz
AQ           1.3631988 sec
RG                2050
DW              20.800 usec
DE               51.93 usec
TE               298.2 K
D1          2.00000000 sec
D11         0.03000000 sec
TD0                  1

======== CHANNEL f1 ========
NUC1               13C
P1                6.43 usec
PL1              -3.00 dB
PL1W       69.66502380 W
SFO1       100.6228298 MHz

======== CHANNEL f2 ========
CPDPRG2        waltz16
NUC2                1H
PCPD2            80.00 usec
PL2              16.00 dB
PL12             15.00 dB
PL13             15.00 dB
PL2W        0.24733528 W
PL12W       0.31137666 W
PL13W       0.31137666 W
SFO2       400.1316005 MHz
SI               32768
SF         100.6127593 MHz
WDW                 EM
SSB                  0
LB                1.00 Hz
GB                   0
PC                1.40
```

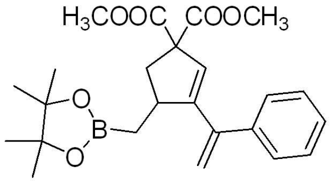

2h

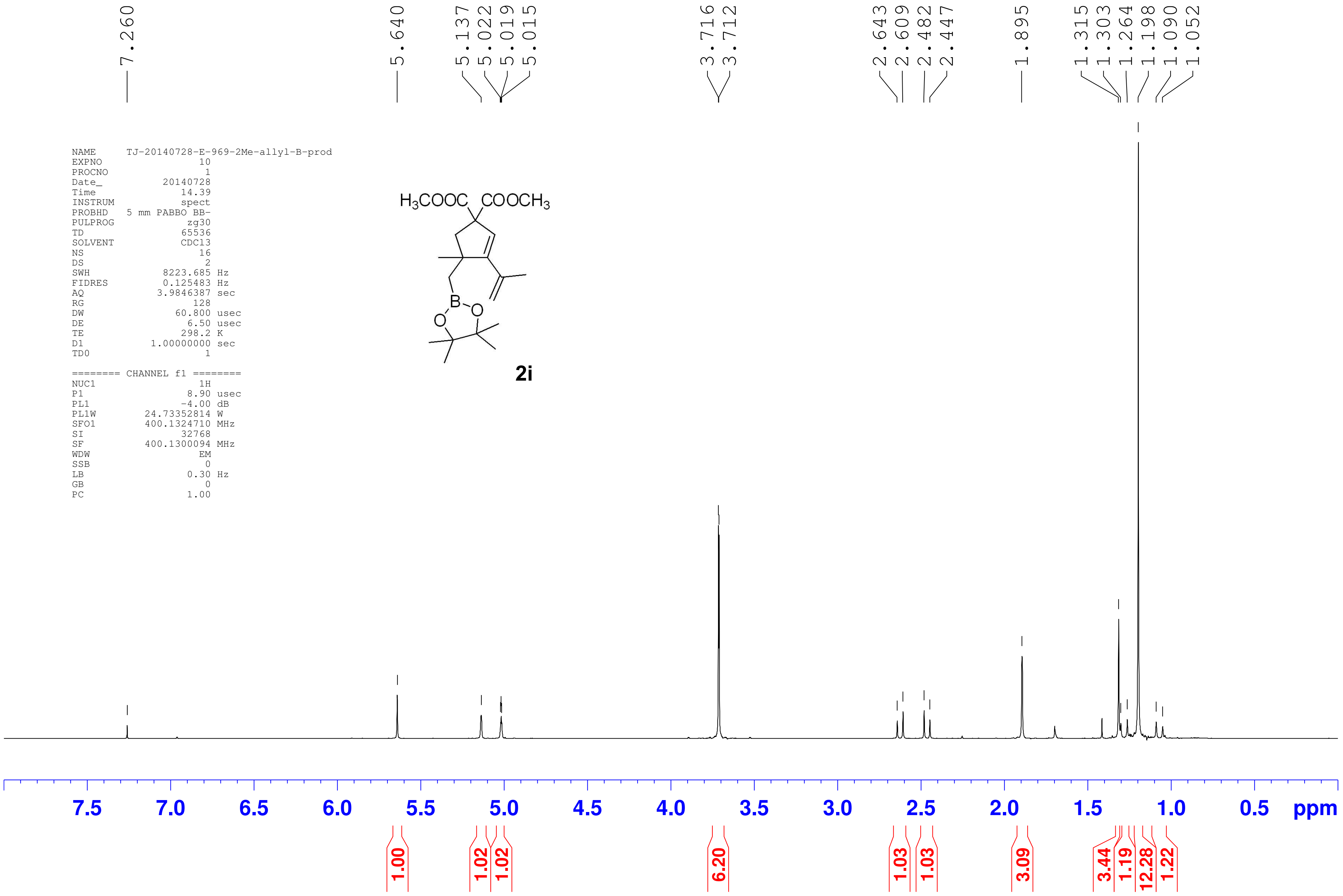
7.260
5.640
5.137
5.022
5.019
5.015
3.716
3.712
2.643
2.609
2.482
2.447
1.895
1.315
1.303
1.264
1.198
1.090
1.052
NAME TJ-20140728-E-969-2Me-allyl-B-prod
EXPNO 10
PROCNO 1
Date_ 20140728
Time 14.39
INSTRUM spect
PROBHD 5 mm PABBO BB-
PULPROG zg30
TD 65536
SOLVENT CDCl3
NS 16
DS 2
SWH 8223.685 Hz
FIDRES 0.125483 Hz
AQ 3.9846387 sec
RG 128
DW 60.800 usec
DE 6.50 usec
TE 298.2 K
D1 1.00000000 sec
TD0 1
======== CHANNEL f1 ========
NUC1 1H
P1 8.90 usec
PL1 -4.00 dB
PL1W 24.73352814 W
SFO1 400.1324710 MHz
SI 32768
SF 400.1300094 MHz
WDW EM
SSB 0
LB 0.30 Hz
GB 0
PC 1.00
7.5 7.0 6.5 6.0 5.5 5.0 4.5 4.0 3.5 3.0 2.5 2.0 1.5 1.0 0.5 ppm
1.00
1.02
1.02
6.20
1.03
1.03
3.09
3.44
1.19
12.28
1.22
H3COOC
COOCH3
B
O
O

2i

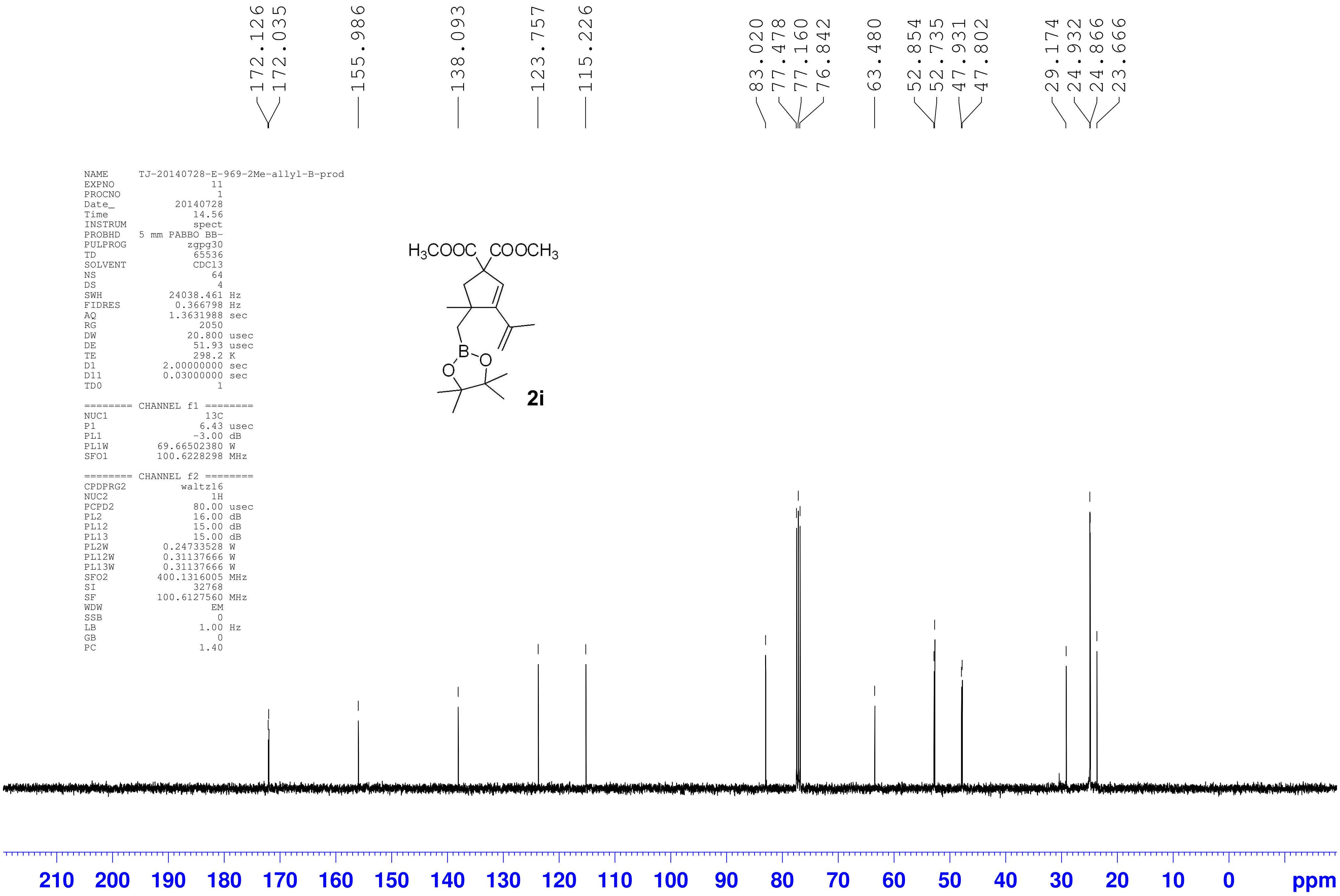


```
NAME      TJ-20140728-E-969-2Me-allyl-B-prod
EXPNO              11
PROCNO              1
Date_        20140728
Time            14.56
INSTRUM         spect
PROBHD   5 mm PABBO BB-
PULPROG        zgpg30
TD              65536
SOLVENT         CDCl3
NS                 64
DS                  4
SWH         24038.461 Hz
FIDRES       0.366798 Hz
AQ          1.3631988 sec
RG               2050
DW             20.800 usec
DE              51.93 usec
TE              298.2 K
D1         2.00000000 sec
D11        0.03000000 sec
TD0                 1

======== CHANNEL f1 ========
NUC1              13C
P1               6.43 usec
PL1             -3.00 dB
PL1W      69.66502380 W
SFO1      100.6228298 MHz

======== CHANNEL f2 ========
CPDPRG2       waltz16
NUC2               1H
PCPD2           80.00 usec
PL2             16.00 dB
PL12            15.00 dB
PL13            15.00 dB
PL2W       0.24733528 W
PL12W      0.31137666 W
PL13W      0.31137666 W
SFO2      400.1316005 MHz
SI              32768
SF        100.6127560 MHz
WDW                EM
SSB                 0
LB               1.00 Hz
GB                  0
PC               1.40
```

2i

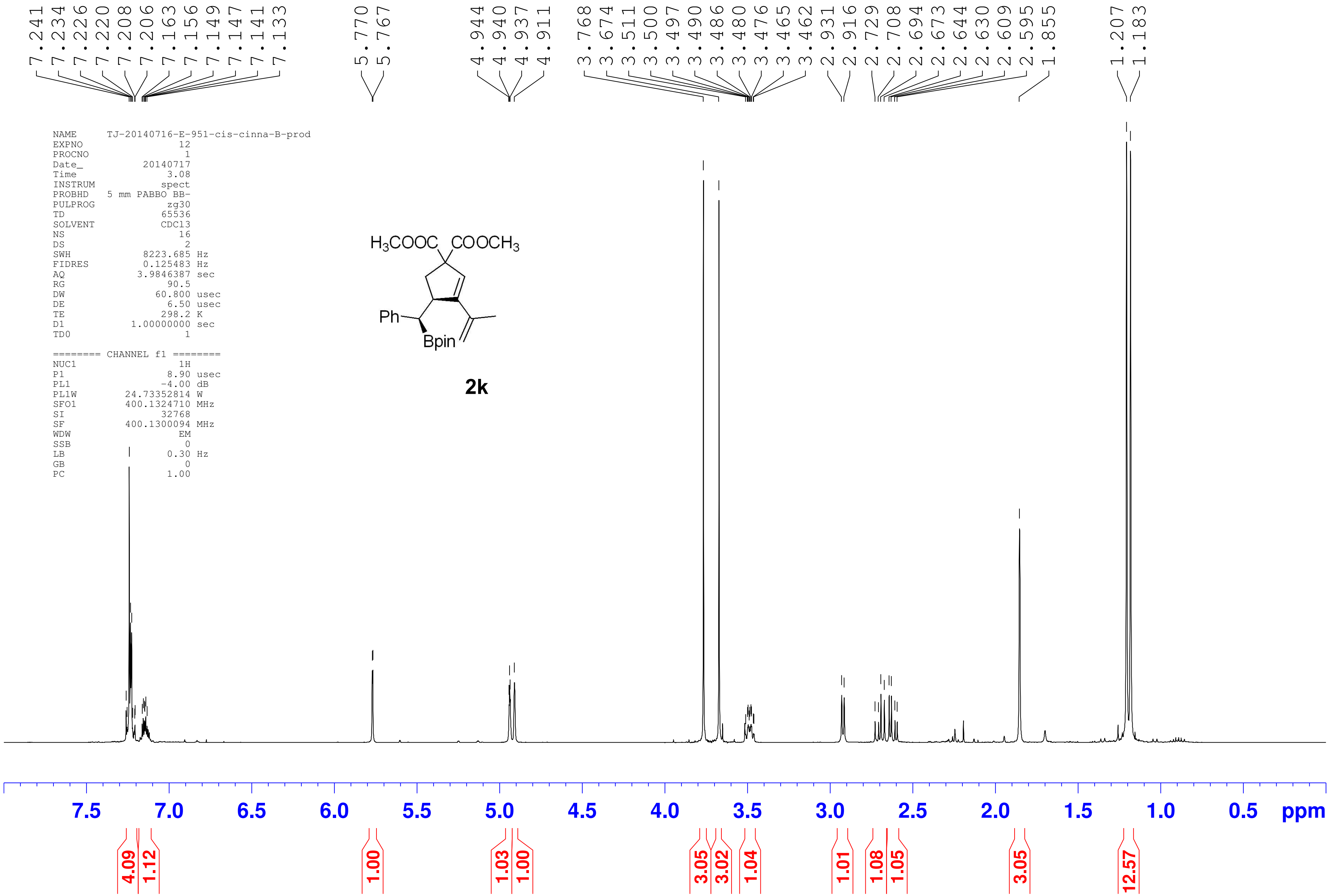
7.241 7.234 7.226 7.220 7.208 7.206 7.163 7.156 7.149 7.147 7.141 7.133
5.770 5.767
4.944 4.940 4.937 4.911
3.768 3.674 3.511 3.500 3.497 3.490 3.486 3.480 3.476 3.465 3.462
2.931 2.916 2.729 2.708 2.694 2.673 2.644 2.630 2.609 2.595 1.855
1.207 1.183
NAME TJ-20140716-E-951-cis-cinna-B-prod
EXPNO 12
PROCNO 1
Date_ 20140717
Time 3.08
INSTRUM spect
PROBHD 5 mm PABBO BB-
PULPROG zg30
TD 65536
SOLVENT CDCl3
NS 16
DS 2
SWH 8223.685 Hz
FIDRES 0.125483 Hz
AQ 3.9846387 sec
RG 90.5
DW 60.800 usec
DE 6.50 usec
TE 298.2 K
D1 1.00000000 sec
TD0 1
======== CHANNEL f1 ========
NUC1 1H
P1 8.90 usec
PL1 -4.00 dB
PL1W 24.73352814 W
SFO1 400.1324710 MHz
SI 32768
SF 400.1300094 MHz
WDW EM
SSB 0
LB 0.30 Hz
GB 0
PC 1.00
H3COOC COOCH3
Ph
Bpin
7.5 7.0 6.5 6.0 5.5 5.0 4.5 4.0 3.5 3.0 2.5 2.0 1.5 1.0 0.5 ppm
4.09 1.12 1.00 1.03 1.00 3.05 3.02 1.04 1.01 1.08 1.05 3.05 12.57

2k

NAME TJ-20140716-E-951-cis-cinna-B-prod
EXPNO 11
PROCNO 1
Date_ 20140716
Time 19.57
INSTRUM spect
PROBHD 5 mm PABBO BB-
PULPROG zgpg30
TD 65536
SOLVENT CDCl3
NS 172
DS 4
SWH 24038.461 Hz
FIDRES 0.366798 Hz
AQ 1.3631988 sec
RG 2050
DW 20.800 usec
DE 51.93 usec
TE 298.2 K
D1 2.00000000 sec
D11 0.03000000 sec
TD0 1

======== CHANNEL f1 ========
NUC1 13C
P1 6.43 usec
PL1 -3.00 dB
PL1W 69.66502380 W
SFO1 100.6228298 MHz

======== CHANNEL f2 ========
CPDPRG2 waltz16
NUC2 1H
PCPD2 80.00 usec
PL2 16.00 dB
PL12 15.00 dB
PL13 15.00 dB
PL2W 0.24733528 W
PL12W 0.31137666 W
PL13W 0.31137666 W
SFO2 400.1316005 MHz
SI 32768
SF 100.6127600 MHz
WDW EM
SSB 0
LB 1.00 Hz
GB 0
PC 1.40

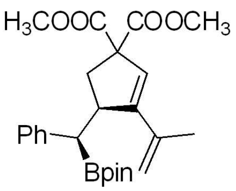

2k

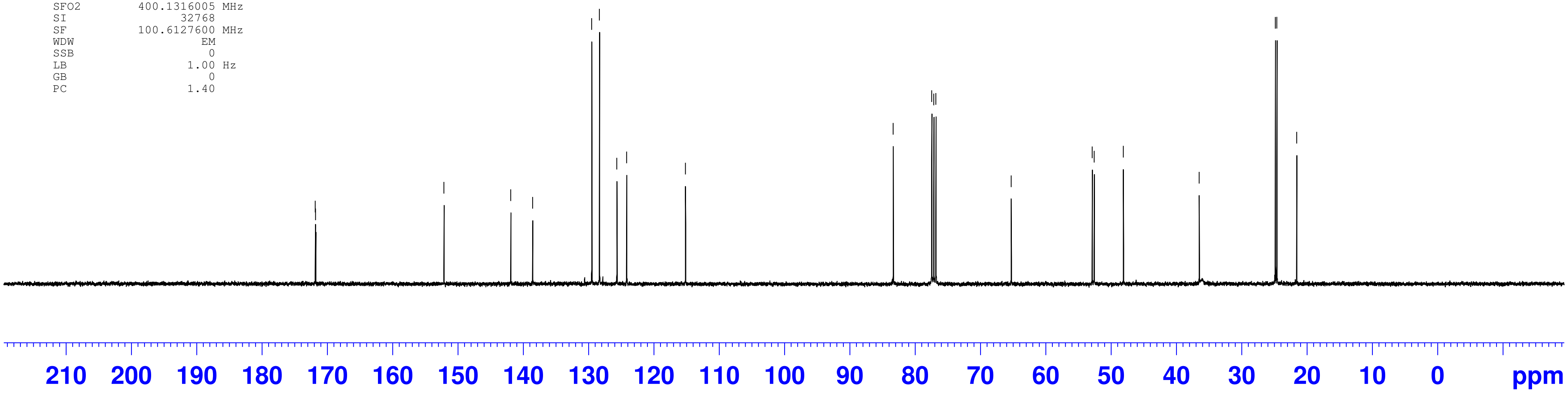

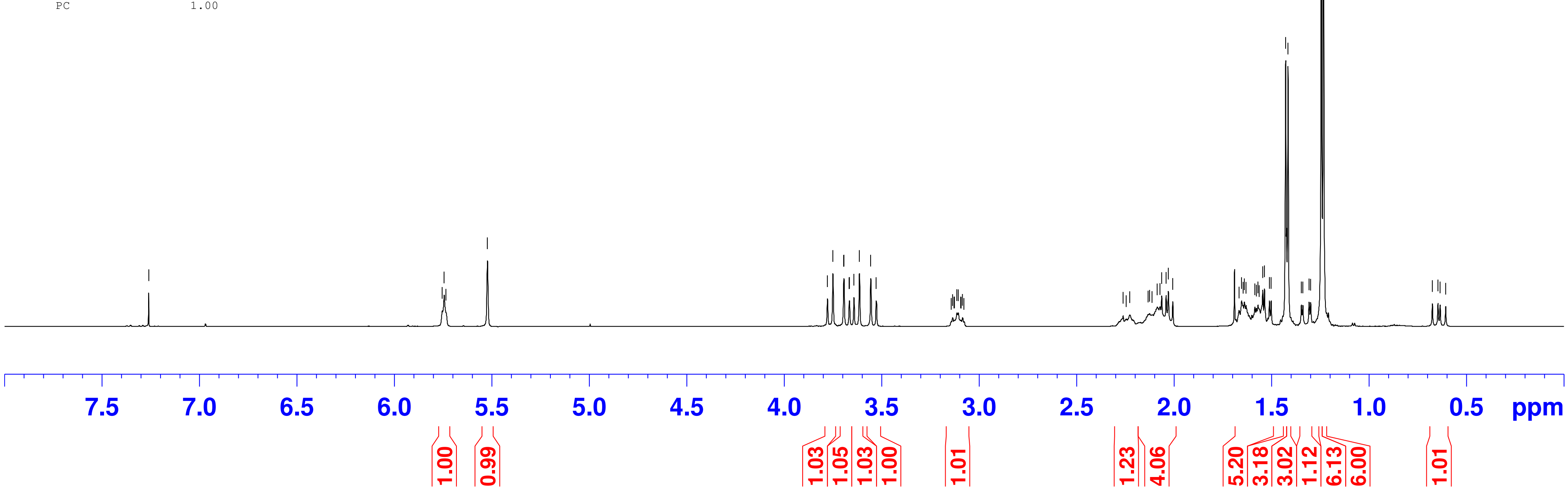


```
NAME      TJ-20141208-F-1042-X-cy-B-rac-prod
EXPNO                 10
PROCNO                 1
Date_           20141209
Time                5.14
INSTRUM            spect
PROBHD   5 mm PABBO BB-
PULPROG             zg30
TD                 65536
SOLVENT            CDCl3
NS                    16
DS                     2
SWH             8223.685 Hz
FIDRES          0.125483 Hz
AQ             3.9846387 sec
RG                   144
DW                60.800 usec
DE                  6.50 usec
TE                 298.2 K
D1            1.00000000 sec
TD0                    1

======== CHANNEL f1 ========
NUC1                  1H
P1                  8.90 usec
PL1                -4.00 dB
PL1W         24.73352814 W
SFO1         400.1324710 MHz
SI                 32768
SF           400.1300094 MHz
WDW                   EM
SSB                    0
LB                  0.30 Hz
GB                     0
PC                  1.00
```

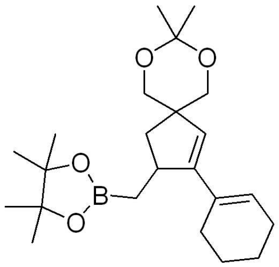

2m

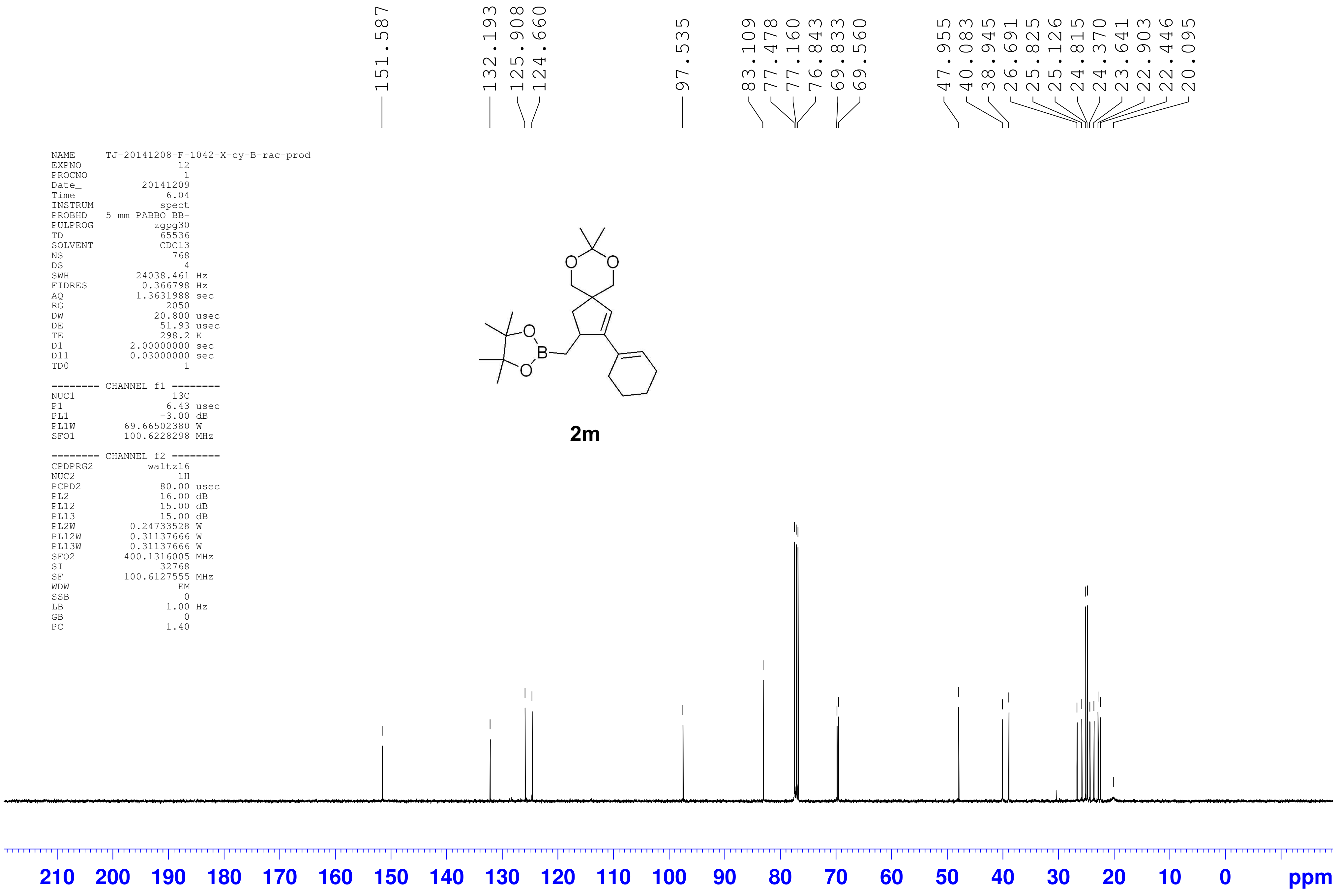
151.587
132.193
125.908
124.660
97.535
83.109
77.478
77.160
76.843
69.833
69.560
47.955
40.083
38.945
26.691
25.825
25.126
24.815
24.370
23.641
22.903
22.446
20.095
NAME TJ-20141208-F-1042-X-cy-B-rac-prod
EXPNO 12
PROCNO 1
Date_ 20141209
Time 6.04
INSTRUM spect
PROBHD 5 mm PABBO BB-
PULPROG zgpg30
TD 65536
SOLVENT CDCl3
NS 768
DS 4
SWH 24038.461 Hz
FIDRES 0.366798 Hz
AQ 1.3631988 sec
RG 2050
DW 20.800 usec
DE 51.93 usec
TE 298.2 K
D1 2.00000000 sec
D11 0.03000000 sec
TD0 1
======== CHANNEL f1 ========
NUC1 13C
P1 6.43 usec
PL1 -3.00 dB
PL1W 69.66502380 W
SFO1 100.6228298 MHz
======== CHANNEL f2 ========
CPDPRG2 waltz16
NUC2 1H
PCPD2 80.00 usec
PL2 16.00 dB
PL12 15.00 dB
PL13 15.00 dB
PL2W 0.24733528 W
PL12W 0.31137666 W
PL13W 0.31137666 W
SFO2 400.1316005 MHz
SI 32768
SF 100.6127555 MHz
WDW EM
SSB 0
LB 1.00 Hz
GB 0
PC 1.40
210 200 190 180 170 160 150 140 130 120 110 100 90 80 70 60 50 40 30 20 10 0 ppm
2m
